# Recruiting Rural Healthcare Providers Today: a Systematic Review of Training Program Success and Determinants of Geographic Choices

**DOI:** 10.1007/s11606-017-4210-z

**Published:** 2017-11-27

**Authors:** Ian T. MacQueen, Melinda Maggard-Gibbons, Gina Capra, Laura Raaen, Jesus G. Ulloa, Paul G. Shekelle, Isomi Miake-Lye, Jessica M. Beroes, Susanne Hempel

**Affiliations:** 1 Department of Surgery, David Geffen School of Medicine at UCLA, Los Angeles, CA USA; 2 Veterans Affairs Greater Los Angeles Healthcare System, Los Angeles, CA USA; 3 Veterans Affairs/Robert Wood Johnson Clinical Scholars Program, UCLA, Los Angeles, CA USA; 4 National Association of Community Health Centers, Bethesda, MD USA; 5 Evidence-Based Practice Center, RAND Corporation, Santa Monica, CA 90407 USA; 6 Department of Surgery, UCSF Medical School, San Francisco, CA USA; 7 Department of Health Policy and Management, UCLA Fielding School of Public Health, Los Angeles, CA USA

**Keywords:** rural health, provider shortages, provider recruitment, student training

## Abstract

**Background:**

Rural areas have historically struggled with shortages of healthcare providers; however, advanced communication technologies have transformed rural healthcare, and practice in underserved areas has been recognized as a policy priority. This systematic review aims to assess reasons for current providers’ geographic choices and the success of training programs aimed at increasing rural provider recruitment.

**Methods:**

This systematic review (PROSPERO: CRD42015025403) searched seven databases for published and gray literature on the current cohort of US rural healthcare practitioners (2005 to March 2017). Two reviewers independently screened citations for inclusion; one reviewer extracted data and assessed risk of bias, with a senior systematic reviewer checking the data; quality of evidence was assessed using the GRADE approach.

**Results:**

Of 7276 screened citations, we identified 31 studies exploring reasons for geographic choices and 24 studies documenting the impact of training programs. Growing up in a rural community is a key determinant and is consistently associated with choosing rural practice. Most existing studies assess physicians, and only a few are based on multivariate analyses that take competing and potentially correlated predictors into account. The success rate of placing providers-in-training in rural practice after graduation, on average, is 44% (range 20–84%; *N* = 31 programs). We did not identify program characteristics that are consistently associated with program success. Data are primarily based on rural tracks for medical residents.

**Discussion:**

The review provides insight into the relative importance of demographic characteristics and motivational factors in determining which providers should be targeted to maximize return on recruitment efforts. Existing programs exposing students to rural practice during their training are promising but require further refining. Public policy must include a specific focus on the trajectory of the healthcare workforce and must consider alternative models of healthcare delivery that promote a more diverse, interdisciplinary combination of providers.

**Electronic supplementary material:**

The online version of this article (10.1007/s11606-017-4210-z) contains supplementary material, which is available to authorized users.

## INTRODUCTION

One fifth of the United States (US) population resides outside metropolitan areas. Patients in these geographically dispersed areas often have to travel great distances to access healthcare and can experience delays in treatment. Rural communities struggle with recruiting as well as retaining healthcare providers, and report provider shortages with ongoing, long-term vacancies.^1^^–^ 4 While estimates differ by provider group, researchers have found that less than 12% of US physicians practice in rural areas.^5^ Hence, while one fifth of the nation’s population resides outside metropolitan areas, only about a tenth of the nation’s physicians are found there.

Rural healthcare undoubtedly requires a particular skill set; for example, healthcare providers are asked to treat a diversity of illnesses within their communities and to perform a wide variety of procedures, often without specialized training.^6^^–^ 11 However, in recent years, strategies have been implemented at the regional, state, and federal levels to increase the number of providers practicing in rural healthcare.^12^^–^ 15 In addition, the care environment has changed in the last decade as a result of the increased use of internet applications and advanced communication technologies.^16^ Telehealth has expanded patient access to care and offers new possibilities for supporting rural healthcare providers, such as access to specialist input through online real-time exchanges and high-quality videoconferencing technology. A comprehensive analysis of 1988 to 1997 graduates suggested that a physician’s hometown was a significant predictor of practice in rural settings.^17^ However, insight is needed into why providers are currently choosing to practice in remote areas, particularly as older analyses may be outdated with respect to the care environment, and research has not been summarized in a comprehensive review across existing studies.^18^ Information on the relative importance of demographic characteristics and motivational factors may provide guidance as to which groups of providers should be targeted to maximize return on recruitment efforts to ameliorate shortages.

Finding ways to encourage physicians to practice in underserved areas has been an ongoing priority for organizations such as the Association of American Medical Colleges (AAMC). In 2006, AAMC called for a 30% increase in MD-granting medical school enrollment by 2015.^19^ A systematic review with literature searches to 2006 highlighted efforts to target healthcare providers in training—for example, by adding rural tracks to medical school programs.^20^ Since publication of the review, a substantial number of new studies have been published. In addition, the impact of programs and reforms implemented to address shortcomings in rural healthcare provision, such as service-requiring scholarships or loan repayment programs, should have become apparent by now.^12^^,^ 14 ^,^ 15

The purpose of this review is to assess and synthesize the evidence for geographic practice choice and effects of training programs on healthcare providers practicing in rural healthcare settings in the US.

## METHODS

This work is part of a larger project commissioned by the U.S. Department of Veterans Affairs (VA) for use in developing policy recommendations regarding rural VA care.^21^ The systematic review was supported by a technical expert panel (TEP) and is registered in PROSPERO (CRD42015025403) and followed PRISMA guidelines.^22^ Two independent reviewers assessed citations for inclusion; discrepancies were reconciled through discussion.

### Search Strategy

We searched PubMed, CINAHL, Web of Science, PsycINFO, ERIC [Education Resources Information Center], WorldCat, and the Grey Literature Report for English-language articles published from 2005 to March 2017. Search terms included “rural” and synonyms (e.g. “non-urban”), healthcare personnel terms, and factors related to “choice” and “training” (see Appendix). We supplemented the search with references mined from pertinent reviews, targeted online resources, and consulted with experts.

### Inclusion Criteria

#### Participants

Based on TEP input, we concentrated on healthcare providers with long training periods that require workforce planning and that are critical for rural community-based outpatient clinics, rural health clinics, and Critical Access Hospitals (family, internal, and emergency medicine physicians; obstetrician/gynecologists, general surgeons, pediatricians, geriatricians, psychiatrists, nurse practitioners, and physician assistants). **Exposure/Training:** We included studies of provider-reported or analytically derived factors potentially associated with geographic choices for practicing in rural care. To assess the success of training, we included studies evaluating the educational programs specific to rural healthcare and programs explicitly aimed at increasing provider recruitment for rural areas. **Study design**: Studies with or without concurrent or historic comparators were eligible. Provider choice studies could report qualitative or quantitative data; training program evaluations had to report numerical data (i.e., a rate). **Outcome:** Studies had to report on practicing in rural care to be eligible. Studies assessing only the intent to practice in rural care were not eligible. **Timing:** Studies reporting on practicing in rural care from 2005 onward were eligible, regardless of the timing of the predictor variables (e.g., growing up in a rural area), evaluation period, exposure duration, or length of follow-up. **Setting:** Studies had to report on rural (as defined by the author) US healthcare settings to be eligible.

### Data Abstraction and Risk of Bias Assessment

One reviewer extracted data, and an experienced systematic reviewer checked the entries. With the help of the TEP, we selected the categories provider characteristics, training, financial aspects, and setting characteristics to differentiate factors potentially influencing geographic practice location. For training program evaluations, we abstracted recruitment success and retention. Publications reporting on the same participants were summarized as one study and counted only once.

We assessed risk of bias for the outcome of interest. Critical appraisal concentrated on the representativeness of the sample (selection bias), the response or follow-up rate (attrition bias), the role of confounding variables (e.g., lack of multivariate analyses), and the data source reporting and reliability (detection bias).^4^

### Synthesis and Quality of Evidence Assessment

For practice choice location data, we presented the presence as well as the absence of associations for all variables of interest that had been analyzed in included studies. We calculated mean, median, mode, and the range of the proportion of providers in training that practiced in rural environments.

The assessment of evidence quality across all identified studies included study limitations, inconsistency in results across studies, imprecision (e.g., due to lack of reported effect sizes), and the magnitude of effect.^21^ Since provider choice factors cannot be analyzed in experimental studies, we did not set the GRADE starting point at low quality of evidence, but considered multivariate analyses to be moderate quality of evidence. We differentiated high, moderate, low, and very low quality of evidence to describe our confidence in the findings among studies.

## RESULTS

Our literature searches identified 7276 citations. Of these, 510 were obtained as full text. The Appendix shows the literature flow diagram and the reasons for exclusion. In total, 50 studies (reported in 64 publications) met the inclusion criteria. Of these, 31 reported predictors of providers’ geographic choices, and 24 studies reported on the success of healthcare provider training programs. Risk of selection bias was rated high in 34% of studies due to the lack of evaluating a representative sample for the population of interest. Risk of attrition bias was rated high in 22% of studies, usually due to a low response rate across eligible participants. Risk of bias due to confounding variables (e.g., through lack of control of competing or correlated variables) was rated high in 36% of studies. All included studies are documented in detail in the evidence table and the risk of bias table in the Appendix.

### Factors Influencing Healthcare Providers’ Geographic Choices for Practice

The 31 identified studies used surveys, qualitative analyses of interviews, or existing data sets to identify predictors for practicing in rural healthcare. Physicians were the focus in most studies, with the remaining addressing physician assistants or a range of healthcare providers. The study samples ranged in number from eight participants, to data sets comprising 322,131^23^ healthcare providers. Most studies defined rurality based on existing coding schemes such as the Rural–Urban Continuum Codes (RUCC).

Table 1 documents factors associated with the choice of practice location identified across studies and the quality of evidence for each finding.

**Table 1:** Factors Influencing Providers’ Geographic Choice of Practice Location

Predictor variable No. of studies GRADE	Effect and direction Results and supporting statement
	*Provider aspects*
Rural background 22 studies GRADE: High*	Positive association • Rural hometown was a predictor in a multivariate analysis of West Virginia medical student graduates (*N* = 1517; OR 4.02; CI 2.17–7.74)^24^ • Significant association with being raised in rural area in multivariate model of Oklahoma State University graduates (*N* = 190, *p* < 0.05) and graduates of the University of Minnesota (*N* = 3365; OR 2.82; CI 2.1–3.79)^25^^,^ 26 • Rural origin was a significant predictor in a multivariate analysis of Michigan State University College of Human Medicine graduates (*N* = 2382; OR 2.80; CI 2.09–3.74)^27^ • Significant association with rural high school in multivariate analysis of West Virginia physician assistants (*N* = 168; *p* < 0.01)^28^ • Being raised in a rural area was associated with practicing in a less populated county in a multivariate analysis (*N* = 683; *p* < 0.05)^29^ • Significant correlation with non-urban high school or college^30^ • Respondents who graduated from a rural high school were significantly more likely to practice in rural settings^31^ • Significant association with population of hometown^32^ • Qualitative analysis suggested rural exposure via upbringing^33^ • Significant difference due to rural childhood^34^^,^ 35 • 70% of rural providers had a rural background^36^ • 60% of rural providers had lived in a rural community^44^ • Birthplace in rural county increased odds^23^ • A combination of growing up in a rural area, plans to practice in rural area, and plans for family medicine showed a positive association^37^ • Higher proportion attending rural high school in rural vs. urban providers^38^ • Significant association with having a rural upbringing39, 47 • Significant relationship with rural background^40^ No association • Majority of rural providers did not grow up in small town^41^^,^ 42
Family 12 studies GRADE: Very low^†‡^	Association • Family ties reported as major reason^43^ • Family/spouse reported to be a very important factor^34^ • Significant association with location partner grew up in^30^ • Proximity to family listed as motivation^36^ • Significant association with having a child during or before medical school^30^ • Conclusion that support of and for significant other was most important factor^31^ • Many interviewees had sought out life partners who were willing to live in a rural community^44^ No association • Having children was not associated with practice location^30^ • Family obligation did not influence decision^36^ • Job of spouse was rated as very important by only 28% of participants^38^ • Spouse’s job location was cited by only 30%^36^ • Proximity to relatives was not a particularly influential factor^40^
Gender 11 studies GRADE: Very low^‡^	Association • Male gender was a significant predictor in a multivariate analysis of Michigan State University College of Human Medicine graduates (*N* = 2382; OR 1.39; CI 1.10–1.75)^27^ • Being male increased odds^23^ • Slightly smaller number of female rural practitioners than in overall population^45^ • Female physicians were less likely to practice in rural areas^46^ No association • Gender was not associated in a multivariate analysis of 1120 University of Louisville medical school graduates^47^ • No significant association with gender in multivariate analysis^28^^,^ 29 ^,^ 47, 48 • No difference by gender group^30^^,^ 31 ^,^ 34
Age 4 studies GRADE: Moderate	No association • Age was not associated with rural practice location in multivariate analysis of 1120 University of Louisville medical school graduates^47^ • Age was not associated with practicing in small town^48^ • Age at graduation was not associated with rural setting for first practice^31^ • Age at graduation, OR 1.03^23^
Marital status 4 studies GRADE: Very low^†‡^	Positive association • Being married increased odds (OR 1.47)^23^ Negative association • Those who were single were significantly more likely to practice in a rural setting as first employment^31^ No association • Being married was not associated^30^^,^ 48
International medical graduate (IMG) 4 studies GRADE: Very low^‡^	Positive association • Odds of South Asian IMGs working in a rural community 1.6 times the odds of US medical graduates in a multivariate analysis (*N* = 3862)^49^ (Slight) negative association • IMGs constituted 22% of the clinically active workforce but 19% of rural PCP workforce^50^ • 15.1% of IMGs work in rural areas compared to 17% of non-IMGs (*p* < 0.001)^51^ No association • 13% of IMGs compared to 18% DOs and 11% MDs were practicing in a rural location^45^
Race, ethnicity 3 studies GRADE: Moderate	No association • Race was not associated with rural practice location in a multivariate analysis of 1120 University of Louisville medical school graduates^47^ • Practicing in small town not associated with race^48^ • Rural setting for first practice not associated with race^31^
Exposure 2 studies GRADE: Low^†^	Positive association • Qualitative analysis suggested exposure via recreation facilitated future rural practice^33^ • Previous time spent in similar area was an important factor^34^
	*Training*
Rural rotation in training or residency 15 studies GRADE: Moderate*^‡^	Positive association • A rural campus was a significant predictor in a multivariate analysis of Michigan State University College of Human Medicine graduates (*N* = 2382; OR 2.80; CI 2.09–3.74)^27^ • Graduates from the University of Louisville medical rural campus were more likely to choose a rural practice location according to a multivariate analysis (*N* = 1120; OR 5.46)^47^ • Rural programs increased odds in addition to being raised in a rural community in a multivariate analysis (*N* = 3365; OR 4.62; CI 3.01–7.07)^25^ • Difference in rural practice between rural- and traditional-track graduates remained significant in a multivariate analysis (*N* = 106; OR 7.54; CI 1.5–37.9)^52^ • Rural residency trainees were 3 times as likely to practice in rural areas^45^ • Interviews suggested that exposure via education facilitated rural practice^33^ • Rural clerkship and rural residency training were associated with rural practice^30^ • Optional summer rural externship increased probability^26^ • Association with medical school in rural area, (OR 2.65); rural elective, (RR 1.53–1.93)^23^ • Significant relationship with rural clerkship^40^ • Many interviewees had developed an interest in rural medicine before or during medical school^44^ No association • University of Mississippi graduates were not more likely to practice in rural areas than physicians who graduated elsewhere^48^ • Medical school had discouraged rural practice for 40% of practitioners^36^ • No association with medical school location^29^ • No difference in rural rotation between rural and urban practitioners^34^ • Study showing a significant relationship with rural clerkship also reported that respondents indicated that participation in rural training was not particularly influential^40^
Primary care and family medicine focus 7 studies GRADE: Moderate	Positive association • Choosing a family medicine residency increased the odds in a multivariate analysis of University of Louisville medical school graduates (*N* = 1120; OR 5.46)^47^ • Primary care specialty was a significant predictor in a multivariate analysis in Michigan State University College of Human Medicine graduates (*N* = 2382; OR 1.65; CI 1.31–2.08)^27^ • Primary care physicians were 2.4 times as likely as specialists to practice in small towns in a multivariate analysis (*N* = 927; *p* < 0.001)^48^ • Rural family medicine residency graduates were 3 times as likely to practice in rural care^45^ • Specialty distribution (primary care, specialty) was significantly different between rural and urban groups^31^ • Association with career in family medicine, (OR 2.65); family medicine clerkship, (RR 1.26–1.44)^23^ • Association with primary care residency (RR 1.22–1.79)^23^ No association No significant association with primary care specialty^35^ Career in primary care OR 1.06^23^
Osteopathic medicine degree 2 studies GRADE: Low^†^	Positive association • 6% of workforce were DOs but 18% practiced in rural care^45^ • 4.9% of the workforce but contributed 10.4% to rural primary care^50^
	*Financial aspects*
Student loan or scholarship 9 studies GRADE: Very low^†‡^	Positive association • Second major reason was a loan or scholarship obligation^43^ • Medical school loan repayment correlated with rural practice^32^ • NHSC loan repayment, NHSC scholarship, and debt increased odds^23^ • Loan repayment program had an important influence on community providers’ choice to practice for 42%^38^ No association • Student loan debt was not a predictor of practicing in small towns^48^^,^ 53 • The amount of loan debt was a less important factor^38^ • For 71%, education debt had no influence on location of initial job^54^ • A loan forgiveness/repayment program was not rated as a particularly influential factor^40^ • Loan repayment was rated an important factor by only 11%^34^
Salary 5 studies GRADE: Very low^†‡^	Association • Importance of income as a factor in practice location differed between rural and urban groups^55^ • 58% found salary to be an important factor^38^ • Pay correlated with selecting rural care^32^ No association • Salary was not a predictor of practicing in small towns in a multivariate analysis^48^ • Salary/signing bonus was rated as very important by only 24–28%^34^
	*Setting*
Scope of practice 6 studies GRADE: Very low^†‡^	Positive association • Broad scope of practice was cited as an important reason of general surgeons^30^ • Scope of practice was important to 71% for healthcare providers^38^ • Most participants had chosen to practice in a rural community, in part, because they could maintain a broad scope of practice^44^ • High agreement with serving the health needs of the community, type of practice, supervising physician characteristics^40^ No association • Scope of practice was rated very important only by 30% of emergency department physicians^34^ • Full scope of practice was important to only 10% of female physicians^36^
Recreational activities 4 studies GRADE: Very low^†‡^	Positive association • Access to amenities/recreation was rated as important for choosing practice location^34^ • Recreational activities were rated as important by 58%^38^ • Hunting of birds and large game was associated with rural practice^30^ No association • Currently hunting or fishing, fishing, and hunting of small game showed no difference^30^ • Cultural and recreational activities, educational facilities in the community, and community recruitment efforts were not a particularly influential factor^40^
Lifestyle, small town life 2 studies GRADE: Low^†^	Positive association • Lifestyle was rated as very important^34^ • Qualitative interviews identified desire for small town life as important^41^

*Upgraded due to size of effect (see text). ^†^Downgraded due to study limitations. ^‡^Downgraded due to inconsistency; for full study details see evidence table in the Appendix

CI confidence interval; IMG international medical graduate; OR odds ratio; NHSC, National Health Service Corps; PCP, primary care physician

### Provider Characteristics

Twenty-two studies were identified that addressed the rural background of providers, and overwhelmingly showed a positive association with the choice to practice in a rural area. This association was also demonstrated in multivariate analyses controlling for competing variables such as rural health track exposure. Effect estimates ranged from an odds ratio (OR) of 4.02 (CI 2.17–7.74)^24^ in a sample of West Virginia medical graduates to an OR of 2.80 (CI 2.09–3.74)^27^ based on an analysis of 30 years of training rural physicians in Michigan. Given the substantial amount of research, confirmation in multiple multivariate analyses by independent author groups, and the large effect, we determined the quality of evidence to be high. We found moderate-quality evidence for a lack of association with age or race: studies that assessed this feature consistency found no association, including multivariate analyses controlling for confounders. Two studies reported on the effect of exposure to rural areas not specific to childhood experiences or provider training (*N* = 22, qualitative interviews; *N* = 197, survey response rate = 67%),^34^^,^ 56 and both suggested a positive association (low quality of evidence due to study limitations and the absence of effect size estimates). Other provider characteristics such as gender, the role of family, marital status, and international medical graduate status were also addressed in several publications, but the results reported were conflicting.

### Training

We identified 15 studies that assessed the effect of rural experience, tracks, or rotations as part of healthcare provider education or training. The largest effect size in a multivariate analysis with a substantial sample size was noted for physicians attending a rural campus in Kentucky (*N* = 31,120, adjusted OR 5.46, CI 2.32–3.69).^47^ An association was confirmed in other multivariate analyses,^27^^,^ 52 including a study reporting an association after adjusting for rural upbringing,^25^ lending support for the importance of training. However, other analytical studies and interview data indicated that rural training was not particularly influential in provider practice choice.^40^ Primary care and family medicine focus were addressed in three studies and were associated with greater odds of practicing in rural care. The largest study (*N* = 322,131; follow-up rate = 98%) reported that a career in family medicine (adjusted OR 2.65; 95% CI 2.51–2.79) or primary care (adjusted OR 1.06; 95% CI 1.01–1.11) was associated with rural practice.^23^ We determined the quality of evidence supporting the effect of training efforts and the predictive value of primary care focus to be moderate. Two studies (*N* = 175,649; *N* = 231,660) demonstrated that osteopathic (vs. allopathic) physicians contribute proportionally more to rural healthcare, but the quality of evidence was rated low because the statistical significance of this difference was not reported and confounding factors were not addressed.^45^^,^ 50

### Financial Aspects

As documented in Table 1, results were mixed regarding the influence of student loans, student debt amount, or participation in scholarship programs (addressed in nine studies) and the importance of salary (addressed in five studies). Both areas were rated as very low quality, because it was not possible to determine whether these aspects were important determinants of practicing in rural healthcare.

### Setting Characteristics

Six studies addressed the scope of practice, and while some studies highlighted the broad scope as the reason for choosing rural practice, others indicated that this was important for only a small proportion of providers. Four studies assessed the influence of recreational activities that rural areas offered, but results were inconsistent within and across studies, depending on the individual predictor. Both areas were rated as having very low-quality evidence, because it cannot be determined whether these characteristics are a significant factor for geographic practice choice. Finally, lifestyle in rural communities was investigated in two studies (*N* = 8, qualitative interviews; *N* = 197, survey response rate = 67%), both reporting an influence on choice of practice location.^34^^,^ 41 The quality of evidence was rated low due to study limitations.

### Training-Based Interventions to Increase Rural Healthcare Provider Recruitment and Retention

We identified 24 studies evaluating rural healthcare professional student and resident training. Most studies evaluated training programs at a single institution (71%), with reported capacity of two to four trainees per year. Seven studies reported on data across multiple training institutions, including an evaluation of 18 medical school rural track programs that were able to document their students’ practice locations (follow-up rate = 60%).^57^ Forty-six percent of the included studies evaluated medical students, and another 46% evaluated medical resident training, most frequently for family medicine residents. One study each assessed nurse practitioners and physician assistants. Study sample size varied widely, with some reporting on a handful of recent graduates and others with data sets evaluating decades of experience with rural training.^27^ The studies utilized internal records, surveys, or the American Medical Association Physician Masterfile for determining practice locations after graduation. Twenty-nine percent of identified studies did not report how “rural” was defined. The majority of training programs consisted of embedding a student or trainee in a rural community for part (or all) of their clinical training, ranging in duration from 4 weeks to 5 years.^58^ In some cases, programs sought to preferentially enroll trainees with an established interest in rural care.^59^

**Figure 1:**
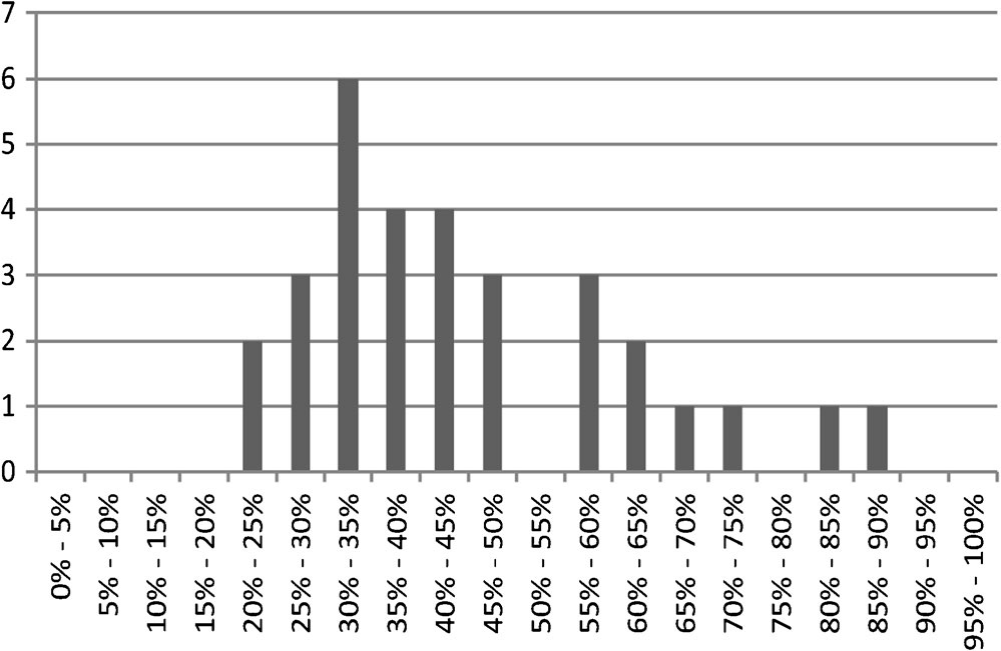
Proportion of students going into rural healthcare after rural-focused training. Note: the figure shows the percentage of all students who completed a rural-focused training program who practiced in rural healthcare as their current or initial job after graduation.

Across studies reporting the proportion of trainees choosing rural practice, success rates varied widely, ranging from 20 to 86%.^57^^,^ 60 However, as Figure 1 demonstrates, the large majority of results were in the range of 30 to 65%; the mean was 44% (median 41%). The figure incorporates data from 31 programs reported across 18 studies.

Given the substantial variation across programs, we explored potential sources of heterogeneity. The rural track programs aimed at medical residents reported a mean success rate of 44% for recruitment into rural practice and did not differ from other programs. Only a small proportion of studies explicitly reported a targeted effort by the school in selecting students with an affinity for rural practice (e.g., preferred access for rural-based students). There was a trend toward higher success rates for programs with longer rural exposure, but we did not detect a systematic effect. Studies that reported on retention of trainees in rural areas indicated that more than half of study participants practiced in the rural areas where they were trained, but data were sparse.^61^^,^ 62

Based on the variation in estimates and the frequent presence of selection bias, we conclude that there is moderate-quality evidence that the success rate of rural training programs ranges from 30 to 65%, and, on average, only one in two trainees is likely to enter rural care.

## DISCUSSION

The principal findings of our review are that growing up in a rural location remains the strongest predictor of choosing a rural practice location, but evidence determining the relative importance of factors is often lacking, and while rural-specific training tracks have some success in rural practice recruitment, the factors contributing to effectiveness are poorly understood.

Understanding the factors influencing providers to practice in a rural location is critical for informing strategies to increase the number of rural providers. Growing up in a rural area remains the strongest predictor and is consistently associated with practicing in a rural community. This aspect has been reported in prior decades, and it appears that it remains a critical factor in today’s rural healthcare environment.^17^ The fact that 30 to 52% of providers from rural backgrounds reported entering rural practice is promising.^25^^,^ 30 Rural recruitment will benefit from further investigation into the specific experiences and characteristics of rural upbringing that increase the likelihood of choosing rural practice. Other factors associated with rural practice were the effect of rural training programs and a primary care and family medicine focus. We did not replicate associations with provider race or the importance of loan forgiveness programs, as suggested in previous predictions for underserved areas in the US.^63^ Multivariate analyses did not detect an effect of race on choosing rural healthcare, and while some of the studies found a correlation with medical school loan repayment,^32^ self-reports from practicing providers did not indicate particular importance; for example, some participants noted that they were already practicing in rural areas when they were made aware of loan forgiveness programs.^40^ The result could be due in part to social desirability effects; however, the relative importance across all considerations influencing the decision to practice in a rural community is simply not known. We note that despite the large number of studies providing data on associations, studies evaluating the relative importance of individual competing and potentially correlated factors, i.e. through multivariate analyses, are scarce.

We identified a substantial number of rural training program evaluations; however, these also mostly addressed medical students and residents. Across all approaches, about 30 to 65% of students exposed to rural-focused training enter these settings. Success rates have not increased based on comparison to a prior review with data to 2006 that reported a success rate of 53 to 64%.^64^ Individual training programs varied widely in format and duration. However, we did not identify factors that systematically and statistically significantly affected success rates, and conclude that the specific aspects of the training experience that influence success need further investigation.

We showed that rural upbringing continues to be a critical aspect, and some training programs specifically select applicants based on their affinity with rural areas. That these providers may constitute less than 1% of the total workforce^23^ has policy implications for the preferential recruitment of students and providers-in-training from rural backgrounds. A recent overview addressing determinants of an urban-origin student choosing rural practice highlighted that barriers to a rural career included lack of opportunities for spouses/partners, children, and continuing medical education.^65^ Accordingly, Deutsch et al. advised: “don’t select medical students—convince them.”^66^ In addition, greater efforts are needed to retain healthcare providers in rural care.^21^^,^ 67

Our review has several limitations. Among included providers, non-physicians were underrepresented, and we did not address provider satisfaction with rural training programs.^68^^,^ 69 There were study limitations such as social desirability effects in interviews determining choice of practice, potential for publication bias and inherent confounding, with training institutions being more motivated to highlight program successes and to publish data on rural placement. Furthermore, the selection of a rural training track is likely to be influenced by an affinity with rural regions, and the success of rural training programs cannot be attributed exclusively to the educational program.^70^ In addition, the definition of rural was operationalized differently across studies, adding heterogeneity. Overall, the conclusions of our review are limited by a relative absence of evidence for many provider types and by limited quality of evidence.

### Conclusions and Policy Implications

This review provides insight into the relative importance of demographic characteristics and motivational factors in determining which groups of providers should be targeted to maximize return on recruitment efforts. Growing up in a rural area remains the strongest predictor of future practice in a rural area, but the number of such students is currently too small for this to be the primary policy option for alleviating rural practice shortages, and studies investigating confounding factors are sparse. Exposing medical students and residents to rural practice during their training is a promising approach for increasing rural healthcare recruiting, but requires further refining and may need to be augmented by recruiting a greater number of prospective students from rural areas. Introducing more healthcare provider students to rural practice is a potentially promising intervention; however, existing programs report limited success, given that half of the exposed students do not chose rural care, and we lack information on variables associated with more successful programs.

Public policy must include a specific focus on the trajectory of the healthcare workforce under continued traditional approaches. It is well established that an adequate supply of healthcare workforce does not, and will not, meet the demand to ensure a healthy population in current or future rural settings. Alternative models of healthcare delivery that promote a more diverse, interdisciplinary combination of providers, educated through a variety of modes, must be seriously considered. Debate should include the financing model for health professional education and health workforce programs, as well as the ability to demonstrate longitudinal value or return on investment on the patchwork of health workforce programs. A national framework for focusing health workforce development at all levels of the educational system and aligning academic, public, and private health workforce investment is critical. A closer, more realistic analysis of the effect of immigration policies, curriculum and training modes for all levels of healthcare staff, and the integration of healthcare technology into the training, education, and support of rural practitioners offers a greater opportunity to mount an adequate response to the current and future crisis.

## Electronic supplementary material

ESM 1 (DOCX 311 kb)
