# Supplementary material for: Recruiting Rural Healthcare Providers Today: a Systematic Review of Training Program Success and Determinants of Geographic Choices
Source: J Gen Intern Med. 2017 Nov 27;33(2):191–9. doi: 10.1007/s11606-017-4210-z (PMC5789104; doi:10.1007/s11606-017-4210-z)
Supplement: Supplementary file 1 — (DOCX 311 kb) [file 11606_2017_4210_MOESM1_ESM.docx]

# Online Appendix

Flow diagram figure

Search strategy

Reasons for exclusion

Evidence tables

Risk of bias assessment

References of included studies

## Figure: Literature Flow Diagram

Studies included in the synthesis
(n = 50)

Full-text articles assessed for eligibility
(n = 510)

Citations screened
(n = 7,276)

Additional citations identified through other sources
(n = 103)

Citations identified through database searching
(n = 7,173)

Full-text articles excluded, with reasons
(n = 339)

•Exclude-Participants: 32

•Exclude-Intervention: 22

•Exclude-Design: 87

•Exclude-Outcome: 149

•Exclude-Timing: 20

•Exclude: Setting: 29

Background (systematic reviews, companion papers)
(n = 121)

Records excluded (not relevant, not US, not provider group of interest)
(n = 6,766)

Studies reporting on geographic choice
(n = 31)

Studies reporting on training program success
(n = 24)

## Search Strategy

### Update search

#### Provider geographic location choice

**DATABASE SEARCHED & TIME PERIOD COVERED:**

**PubMed – 1/1/2015-3/7/2017**

**LANGUAGE:**

English

**SEARCH STRATEGY:**

((Rural*[tiab] OR agricultur*[tiab] OR wilderness* OR frontier*[tiab] OR (native AND reservation*) OR farmers OR farming OR nonurban* OR "non-urban" OR remote*[tiab] OR isolated[tiab] OR "small town" OR "small towns" OR village*[tiab] OR settlement*[tiab] OR "Rural Population"[Mesh]) AND ((practice* OR health care OR rural care) OR ("Rural Health Services"[Mesh] OR "Rural Health"[Mesh] OR "Hospitals, Rural"[Mesh]))) OR “country side” OR “countryside” OR “pastoral setting”

AND

("Health Personnel"[Mesh] OR physician*[tiab] OR nurse provider[tiab] OR nurse providers[tiab] OR hospitalist*[tiab] OR hospital staff*[tiab] OR healthcare professional*[tiab] OR health care professional*[tiab] OR doctor[tiab] OR doctors[tiab] OR health manpower[mh] OR medical staff, hospital[mh] OR obstetrician* OR gynecologist* OR general surgeon* OR pediatrician* OR geriatrician* OR psychiatrist*) OR clinician*

AND

(choice* OR choos* OR decision* OR decid* OR incentive* OR attract* OR influen* OR select* OR predict*)

AND

(usa[Affiliation] OR AL[Affiliation] OR AK[Affiliation] OR AZ[Affiliation] OR AR[Affiliation] OR CA[Affiliation] OR CO[Affiliation] OR CT[Affiliation] OR DE[Affiliation] OR FL[Affiliation] OR GA[Affiliation] OR HI[Affiliation] OR ID[Affiliation] OR IL[Affiliation] OR IN[Affiliation] OR IA[Affiliation] OR KS[Affiliation] OR KY[Affiliation] OR LA[Affiliation] OR ME[Affiliation] OR MD[Affiliation] OR MA[Affiliation] OR MI[Affiliation] OR MN[Affiliation] OR MS[Affiliation] OR MO[Affiliation] OR MT[Affiliation] OR NE[Affiliation] OR NV[Affiliation] OR NH[Affiliation] OR NJ[Affiliation] OR NM[Affiliation] OR NY[Affiliation] OR NC[Affiliation] OR ND[Affiliation] OR OH[Affiliation] OR OK[Affiliation] OR OR[Affiliation] OR PA[Affiliation] OR RI[Affiliation] OR SC[Affiliation] OR SD[Affiliation] OR TN[Affiliation] OR TX[Affiliation] OR UT[Affiliation] OR VA[Affiliation] OR VT[Affiliation] OR WA[Affiliation] OR WV[Affiliation] OR WI[Affiliation] OR WY[Affiliation] OR Alabama[Affiliation] OR Alaska[Affiliation] OR Arizona[Affiliation] OR Arkansas[Affiliation] OR California[Affiliation] OR Colorado[Affiliation] OR Connecticut[Affiliation] OR Delaware[Affiliation] OR Florida[Affiliation] OR Georgia[Affiliation] OR Hawaii[Affiliation] OR Idaho[Affiliation] OR Illinois[Affiliation] OR Indiana[Affiliation] OR Iowa[Affiliation] OR Kansas[Affiliation] OR Kentucky[Affiliation] OR Louisiana[Affiliation] OR Maine[Affiliation] OR Maryland[Affiliation] OR Massachusetts[Affiliation] OR Michigan[Affiliation] OR Minnesota[Affiliation] OR Mississippi[Affiliation] OR Missouri[Affiliation] OR Montana[Affiliation] OR Nebraska[Affiliation] OR Nevada[Affiliation] OR New Hampshire[Affiliation] OR New Jersey[Affiliation] OR New Mexico[Affiliation] OR New York[Affiliation] OR North Carolina[Affiliation] OR North Dakota[Affiliation] OR Ohio[Affiliation] OR Oklahoma[Affiliation] OR Oregon[Affiliation] OR Pennsylvania[Affiliation] OR Rhode Island[Affiliation] OR South Carolina[Affiliation] OR South Dakota[Affiliation] OR Tennessee[Affiliation] OR Texas[Affiliation] OR Utah[Affiliation] OR Vermont[Affiliation] OR Virginia[Affiliation] OR Washington[Affiliation] OR West Virginia[Affiliation] OR Wisconsin[Affiliation] OR Wyoming[Affiliation] OR united states[Affiliation])

**Results: 485 (489, but 4 were duplicates)**

**DATABASE SEARCHED & TIME PERIOD COVERED:**

**CINAHL – 1/1/2015-3/7/2017**

**LANGUAGE:**

English

**Geographic Subset:**
USA

**SEARCH STRATEGY #1:**

((Rural*[tiab] OR agricultur*[tiab] OR wilderness* OR frontier*[tiab] OR (native AND reservation*) OR farmers OR farming OR nonurban* OR "non-urban" OR remote*[tiab] OR isolated[tiab] OR "small town" OR "small towns" OR village*[tiab] OR settlement*[tiab] OR "country side" OR countryside OR "pastoral setting" OR (MH "Rural Population") OR (MH "Rural Health Centers") OR (MH "Hospitals, Rural") OR (MH "Rural Health Services")

AND

"Health Personnel" OR physician* OR "nurse provider" OR "nurse providers" OR hospitalist* OR hospital staff* OR "healthcare professional*" OR "health care professional*" OR doctor OR doctors OR "health manpower" OR clinician* ) OR (MH "Rural Health Personnel"))

AND

choice* OR choos* OR decision* OR decid* OR incentive* OR attract* OR influen* OR select* OR predict*

(used USA geographic subset and there really isn’t a filter for empirical only)

**Results: 27 – duplicates = 12**

**DATABASE SEARCHED & TIME PERIOD COVERED:**

**PsycINFO – 1/1/2015-3/7/2017**

**LANGUAGE:**

English
**Source**:
Academic Journals

**SEARCH STRATEGY:**

(rural* OR agricultur* OR wilderness* OR TI frontier* OR AB frontier* OR (native AND reservation*) OR farmer* OR farming OR nonurban* OR "non-urban" OR remote* OR isolated OR "small town" OR "small towns" OR village* OR settlement* OR "Rural Population" OR "Rural Health Services" OR "Rural Health" OR "Hospitals, Rural" OR "Rural Hospital*" OR "country side" OR countryside OR "pastoral setting"

AND

"Health Personnel" OR physician* OR hospitalist* OR "hospital staff*" OR "healthcare professional*" OR "health care professional*" OR doctor OR doctors OR manpower OR workforce OR "hospital medical staff" OR "family medicine" OR "internal medicine" OR pediatrician* OR pediatric* OR general surgeon* OR psychiatrist* OR geriatrician* OR obstetrician* OR gynecologist* OR "nurse practitioner*" OR "physician assistant*" OR clinician*

AND

choice* OR choos* OR decision* OR decid* OR incentive* OR attract* OR influen* OR select* OR predict* )

NOT

PL (Tanzania OR Ukraine OR Lebanon OR Malawi OR Uganda OR nigeria OR japan OR China OR sweden OR switzerland OR england OR great britain OR united kingdom OR spain OR germany OR france OR italy OR poland OR ireland OR wales OR australia OR new zealand OR mexico OR canada OR portugal OR egypt OR jordan OR iran OR singapore OR russia OR belgium OR africa OR brazil OR noway OR finland OR netherlands OR zimbabwe OR jordan OR iran OR Iraq OR turkey OR peru OR belize OR chile OR Scotland OR Iceland OR Greenland OR Mongolia OR Algeria OR Botswana OR cameroon OR Ghana OR Ethiopia OR kenya OR Libya OR Korea OR morocco OR Namibia OR Rwanda OR Senegal OR sierra leone OR Somalia OR Swaziland OR sudan OR Tunisia OR Zambia OR Israel OR Greece OR hungary OR Austria OR Denmark OR Bulgaria OR Croatia OR Slovenia OR Lithuania OR Kosovo OR Cyprus OR Czech Republic OR Serbia OR Philippines OR Fiji OR India OR Vietnam OR Nepal OR Sri Lanka OR North Korea OR South Korea OR Malaysia OR gaza)

**Results: 207 – duplicates = 150**

**DATABASE SEARCHED & TIME PERIOD COVERED:**

Web of Science– 1/1/2015-3/7/2017

**LANGUAGE:**

English

**Countries/Territories**USA

**Document Types:**Article

**SEARCH STRATEGY:**

ts=(Rural* OR agricultur* OR wilderness* OR frontier* OR (native AND reservation*) OR farm OR farmer* OR farmers OR farming OR nonurban* OR "non-urban" OR remote* OR isolated OR "small town" OR "small towns" OR village* OR settlement* OR "country side" OR countryside OR "pastoral setting")

AND

ts=("Health Personnel" OR physician* OR hospitalist* OR "hospital staff" OR healthcare professional* OR "health care professional*" OR doctor OR doctors OR "health manpower" OR "medical staff" OR "family medicine" OR "internal medicine" OR pediatrician* OR "general surgeon*" OR psychiatrist* OR geriatrician* OR obstetrician* OR gynecologist* OR "nurse practitioner*" OR "physician assistant*" OR clinician*)

AND

ts=(choice* OR choos* OR decision* OR decid* OR incentive* OR attract* OR influen* OR select* OR predict*)

**Results: 386 – duplicates = 197**

**DATABASE SEARCHED & TIME PERIOD COVERED:**

WorldCat– 2015-2017
Limit to file: Internet

**LANGUAGE:**

  English

**SEARCH STRATEGY:**

kw: Rural* OR kw: agricultur* OR kw: wilderness* OR kw: frontier* OR (kw: native AND kw: reservation*) OR kw: farmer OR kw: farmers OR kw: farming OR kw: farm OR kw: farms OR kw: nonurban* OR kw: non-urban OR kw: remote* OR kw: isolate* OR kw: small w town OR kw: small w towns OR kw: village* OR kw: settlement* OR kw: countryside OR kw: country side OR (kw: pastoral and kw: setting)

AND

kw: Health w Personnel OR kw: physician* OR kw: nurses OR kw: nursing OR kw: hospitalist* OR (kw: hospital and kw: staff) OR (kw: healthcare and kw: professional*) OR ((kw: health and kw: care and kw: professional*) OR kw: doctor) OR kw: doctors OR kw: family w medicine OR kw: internal w medicine OR kw: pediatrician* OR kw: pediatric* OR (kw: general and kw: surgeon*) OR kw: psychiatrist* OR kw: geriatric* OR kw: obstetric* OR kw: gynecologis* OR (kw: nurse and kw: practitioner*) OR (kw: physician and kw: assistant*) OR kw: clinician

AND

kw: choice* OR kw: choos* OR kw: decision* OR kw: decid* OR kw: select* OR kw: predict*

AND

kw: incentive* OR kw: attract* OR kw: pecuniary OR kw: non-pecuniary OR kw: income OR kw: monetary OR kw: economic* OR kw: financial OR kw: opportunit* OR kw: debt* OR kw: influen*

FILTERED TO NON-JUVENILE, NON-FICTION

Results: 374 records – duplicates within database = 329

**======**

#### Training and Education

**DATABASE SEARCHED & TIME PERIOD COVERED:**

PUBMED - 1/1/2015-3/7/2017

**LANGUAGE:**

English

**SEARCH STRATEGY:**

((Rural*[tiab] OR agricultur*[tiab] OR wilderness* OR frontier*[tiab] OR (native AND reservation*) OR farmers OR farming OR nonurban* OR "non-urban" OR remote*[tiab] OR isolated[tiab] OR "small town" OR "small towns" OR village*[tiab] OR settlement*[tiab] OR "Rural Population"[Mesh]) AND ((practice* OR health care OR rural care) OR ("Rural Health Services"[Mesh] OR "Rural Health"[Mesh] OR "Hospitals, Rural"[Mesh]))) OR “country side” OR “countryside” OR “pastoral setting”

AND

("Schools, Health Occupations"[Mesh] OR "Students, Health Occupations"[Mesh] OR internship and residency[mh] OR graduate[tiab] OR post-graduate[tiab] OR postgraduate[tiab] OR college[tiab] OR medical school[tiab])

AND

(training OR train[ti] OR educat*[tiab] OR medical education OR education, professional[MeSH)

AND

(Medical OR medicine OR "Health Personnel" OR physician* OR hospitalist* OR hospital staff* OR healthcare professional* OR health care professional* OR doctor OR doctors OR manpower OR workforce hospital OR "family medicine" OR "internal medicine" OR pediatrician* OR pediatric* OR general surgeon* OR psychiatrist* OR geriatrician* OR obstetrician* OR gynecologist* OR nurse practitioner* OR physician assistant* OR resident[tiab]) OR clinician*

AND

(Program[tiab] OR programs[tiab] OR rural track[tiab] OR effort[tiab] OR efforts[tiab] OR evaluat*[tiab] OR improv*[tiab] OR success*[tiab] OR effectiveness[tiab] OR efficacy[tiab] OR survey*[tiab] OR questionnaire*[tiab])

AND

(usa[Affiliation] OR AL[Affiliation] OR AK[Affiliation] OR AZ[Affiliation] OR AR[Affiliation] OR CA[Affiliation] OR CO[Affiliation] OR CT[Affiliation] OR DE[Affiliation] OR FL[Affiliation] OR GA[Affiliation] OR HI[Affiliation] OR ID[Affiliation] OR IL[Affiliation] OR IN[Affiliation] OR IA[Affiliation] OR KS[Affiliation] OR KY[Affiliation] OR LA[Affiliation] OR ME[Affiliation] OR MD[Affiliation] OR MA[Affiliation] OR MI[Affiliation] OR MN[Affiliation] OR MS[Affiliation] OR MO[Affiliation] OR MT[Affiliation] OR NE[Affiliation] OR NV[Affiliation] OR NH[Affiliation] OR NJ[Affiliation] OR NM[Affiliation] OR NY[Affiliation] OR NC[Affiliation] OR ND[Affiliation] OR OH[Affiliation] OR OK[Affiliation] OR OR[Affiliation] OR PA[Affiliation] OR RI[Affiliation] OR SC[Affiliation] OR SD[Affiliation] OR TN[Affiliation] OR TX[Affiliation] OR UT[Affiliation] OR VA[Affiliation] OR VT[Affiliation] OR WA[Affiliation] OR WV[Affiliation] OR WI[Affiliation] OR WY[Affiliation] OR Alabama[Affiliation] OR Alaska[Affiliation] OR Arizona[Affiliation] OR Arkansas[Affiliation] OR California[Affiliation] OR Colorado[Affiliation] OR Connecticut[Affiliation] OR Delaware[Affiliation] OR Florida[Affiliation] OR Georgia[Affiliation] OR Hawaii[Affiliation] OR Idaho[Affiliation] OR Illinois[Affiliation] OR Indiana[Affiliation] OR Iowa[Affiliation] OR Kansas[Affiliation] OR Kentucky[Affiliation] OR Louisiana[Affiliation] OR Maine[Affiliation] OR Maryland[Affiliation] OR Massachusetts[Affiliation] OR Michigan[Affiliation] OR Minnesota[Affiliation] OR Mississippi[Affiliation] OR Missouri[Affiliation] OR Montana[Affiliation] OR Nebraska[Affiliation] OR Nevada[Affiliation] OR New Hampshire[Affiliation] OR New Jersey[Affiliation] OR New Mexico[Affiliation] OR New York[Affiliation] OR North Carolina[Affiliation] OR North Dakota[Affiliation] OR Ohio[Affiliation] OR Oklahoma[Affiliation] OR Oregon[Affiliation] OR Pennsylvania[Affiliation] OR Rhode Island[Affiliation] OR South Carolina[Affiliation] OR South Dakota[Affiliation] OR Tennessee[Affiliation] OR Texas[Affiliation] OR Utah[Affiliation] OR Vermont[Affiliation] OR Virginia[Affiliation] OR Washington[Affiliation] OR West Virginia[Affiliation] OR Wisconsin[Affiliation] OR Wyoming[Affiliation] OR united states[Affiliation])

**Results: 122 – duplicates = 70**

**DATABASE SEARCHED & TIME PERIOD COVERED:**

CINAHL – 1/1/2015-3/7/2017

**LANGUAGE:**

English

**Geographic Subset:**
USA

**SEARCH STRATEGY:**

((Rural*[tiab] OR agricultur*[tiab] OR wilderness* OR frontier*[tiab] OR (native AND reservation*) OR farmers OR farming OR nonurban* OR "non-urban" OR remote*[tiab] OR isolated[tiab] OR "small town" OR "small towns" OR village*[tiab] OR settlement*[tiab] OR "country side" OR countryside OR "pastoral setting" OR (MH "Rural Population") OR (MH "Rural Health Centers") OR (MH "Hospitals, Rural") OR (MH "Rural Health Services")

AND

(Medical OR medicine OR "Health Personnel" OR physician* OR hospitalist* OR hospital staff* OR healthcare professional* OR health care professional* OR doctor OR doctors OR manpower OR workforce hospital OR "family medicine" OR "internal medicine" OR pediatrician* OR pediatric* OR general surgeon* OR psychiatrist* OR geriatrician* OR obstetrician* OR gynecologist* OR nurse practitioner* OR physician assistant* OR resident[tiab] OR clinician*)) OR (MH "Rural Health Personnel"))

AND

(MH "Schools, Health Occupations+") OR (MH "Internship and Residency") OR graduate[tiab] OR post-graduate[tiab] OR postgraduate[tiab] OR college[tiab] OR medical school[tiab]

AND

training OR train[ti] OR educat*[tiab] OR medical education OR Program[tiab] OR programs[tiab] OR rural track[tiab] OR effort[tiab] OR efforts[tiab] OR evaluat*[tiab] OR improv*[tiab] OR success*[tiab] OR effectiveness[tiab] OR efficacy[tiab] OR survey*[tiab] OR questionnaire*[tiab]

**Results: 1 – duplicate = 0**

**DATABASE SEARCHED & TIME PERIOD COVERED:**

ERIC– 1/1/2015-3/7/2017

**LANGUAGE:**

English

**Source Types:**
Academic Journals, ERIC Documents, Educational Reports

**SEARCH STRATEGY:**

Rural* OR agricultur* OR wilderness* OR frontier* OR (native AND reservation*) OR farmers OR farming OR nonurban* OR "non-urban" OR remote* OR "small town" OR "small towns" OR village* OR settlement* OR "Rural Population" OR "Rural Health Services" OR "Rural Health" OR "Hospitals, Rural" OR "country side" OR countryside OR "pastoral setting"

AND

"Health Personnel" OR physician* OR nurse practitioner OR nurse practitioners OR hospitalist* OR hospital staff* OR healthcare professional* OR health care professional* OR doctor OR doctors OR manpower OR workforce OR medical staff, hospital OR "family medicine" OR "internal medicine" OR pediatrician* OR general surgeon* OR psychiatrist* OR geriatrician* OR obstetrician* OR gynecologist* OR physician assistant* OR clinician*

AND

training OR train OR trained OR educat* OR graduat* OR post-graduate OR postgraduate OR college OR rural track OR program*

(no US Filter or Empirical filter)
**Results:** **51 – duplicates = 49**

**DATABASE SEARCHED & TIME PERIOD COVERED:**

Web of Science– 1/1/2015-3/7/2017

**LANGUAGE:**

English

**Countries/Territories**USA

**Document Types:**Article

**SEARCH STRATEGY:**

ts=(Rural* OR agricultur* OR wilderness* OR frontier* OR (native AND reservation*) OR farmer* OR farmers OR farming OR farm OR farms OR nonurban* OR "non-urban" OR remote* OR "small town" OR "small towns" OR village* OR settlement* OR "country side" OR countryside OR "pastoral setting")

AND

ts=("Health Personnel" OR physician* OR hospitalist* OR "hospital staff" OR "healthcare professional*" OR "health care professional*" OR doctor OR doctors OR "health manpower" OR "medical staff" OR "family medicine" OR "internal medicine" OR pediatrician* OR "general surgeon*" OR psychiatrist* OR geriatrician* OR obstetrician* OR gynecologist* OR "nurse practitioner*" OR "physician assistant*" OR clinician*)

AND

ts=(training OR train OR trained OR educat* OR graduat* OR post-graduate OR postgraduate OR college)

AND

ts=(program OR programs OR effort OR efforts OR evaluat* OR improv* OR success* OR efficacy OR survey* OR questionnaire*)

**Results: 259 – duplicates = 138**

**257 unique for Training and Education**

**TOTAL FOR BOTH SEARCHES (GC/TE): 1101**

#### Geographic Choice and Training and Education

**DATABASE SEARCHED & TIME PERIOD COVERED:**

GREY LITERATURE REPORT – 1/1/2015-2017

**SEARCH STRATEGY:**

Rural = 24 results

### Original search

#### Rural Health Provider Needs

**DATABASE SEARCHED & TIME PERIOD COVERED:**

PubMed – 1/1/2005-2/11/2015

**SEARCH STRATEGY:**

rural*[tiab] OR agricultur*[tiab] OR wilderness* OR frontier* OR (native AND reservation*) OR farmer OR farmers OR farming OR farm OR farms OR nonurban* OR "non-urban" OR remote*[tiab] OR outback* OR isolated[tiab] OR "small town" OR "small towns" OR village*[tiab] OR settlement* OR "Rural Population"[Mesh] OR "Rural Nursing"[Mesh] OR "Rural Health Services"[Mesh] OR "Rural Health"[Mesh] OR "Hospitals, Rural"[Mesh]

AND

"Health Personnel"[Mesh] OR physician*[tiab] OR nurses[tiab] OR nursing[tiab] OR hospitalist*[tiab] OR hospital staff*[tiab] OR healthcare professional*[tiab] OR health care professional*[tiab] OR doctor[tiab] OR doctors[tiab] OR health manpower[mh] OR manpower[tiab] OR workforce OR medical staff, hospital OR "family medicine" OR "internal medicine" OR pediatrician* OR pediatric* OR general surgeon* OR psychiatrist* OR geriatric* OR obstetric* OR gynecologis* OR nurse practitioner* OR physician assistant*

AND

need[tiab] OR needs[tiab] OR needed[tiab] OR needing[tiab] OR supply[tiab] OR demand[tiab] OR "supply and distribution" [Subheading] OR shortage

AND

predict* OR projected OR future OR trend* OR forecast*

**DATABASE SEARCHED & TIME PERIOD COVERED:**

CINAHL – 1/1/2005-2/11/2015

**SEARCH STRATEGY:**

rural* OR agricultur* OR wilderness* OR frontier* OR (native AND reservation*) OR farm OR farmer* OR farmers OR farming OR farm OR farms OR nonurban* OR "non-urban" OR remote* OR isolated OR "small town" OR "small towns" OR village* OR settlement* OR "Rural Population" OR "Rural Health Services" OR "Rural Health" OR "Hospitals, Rural"

AND

"Health Personnel" OR physician* OR nurses OR nursing OR hospitalist* OR hospital staff* OR healthcare professional* OR health care professional* OR doctor OR doctors OR manpower

Narrow by SubjectGeographic: - usa

**DATABASE SEARCHED & TIME PERIOD COVERED:**

WorldCat – 1/1/2005-2/11/2015

**LANGUAGE:**

English

**SEARCH STRATEGY:**

kw: (Rural* OR kw: agricultur* OR kw: wilderness* OR kw: frontier* OR (kw: native AND kw: reservation*) OR kw: farmer OR kw: farmers OR kw: farming OR kw: farm OR kw: farms OR kw: nonurban* OR kw: non-urban OR kw: remote* OR kw: isolate* OR kw: small w town OR kw: small w towns OR kw: village* OR kw: settlement*)

AND

kw: (Health w Personnel OR kw: physician* OR kw: nurses OR kw: nursing OR kw: hospitalist* OR (kw: hospital and kw: staff) OR (kw: healthcare and kw: professional*) OR ((kw: health and kw: care and kw: professional*) OR kw: doctor) OR kw: doctors OR kw: family w medicine OR kw: internal w medicine OR kw: pediatrician* OR kw: pediatric* OR (kw: general and kw: surgeon*) OR kw: psychiatrist* OR kw: geriatric* OR kw: obstetric* OR kw: gynecologis* OR (kw: nurse and kw: practitioner*) OR (kw: physician and kw: assistant*))

AND

kw: (need OR kw: needs OR kw: needed OR kw: needing OR kw: supply OR kw: demand OR kw: distribut*)

AND

kw: predict* OR kw: projected OR kw: future OR kw: trend* OR kw: forecast*

FILTERED TO BOOKS, NON-JUVENILE, NON-FICTION

**DATABASE SEARCHED & TIME PERIOD COVERED:**

Web of Science – 1/1/2005-2/12/2015

**LANGUAGE:**

English

**SEARCH STRATEGY:**

ts=(Rural* OR agricultur* OR wilderness* OR frontier* OR (native AND reservation*) OR farm OR farmer* OR farmers OR farming OR farm OR farms OR nonurban* OR "non-urban" OR remote* OR isolated OR "small town" OR "small towns" OR village* OR settlement*) AND ts=("Health Personnel" OR physician* OR nurses OR nursing OR hospitalist* OR "hospital staff" OR healthcare professional* OR health care professional* OR doctor OR doctors OR "health manpower" OR "medical staff" OR "family medicine" OR "internal medicine" OR pediatrician* OR pediatric* OR general surgeon* OR psychiatrist* OR geriatric* OR obstetric* OR gynecologis* OR nurse practitioner* OR physician assistant*)

AND

ts=(need OR needs OR needed OR needing OR supply OR demand OR distribut* OR shortage*)

AND

ts=(predict* OR projected OR future OR trend* or forecast*)

Refined by: COUNTRIES/TERRITORIES: ( USA )

**==========================================================================**

#### DECISION FACTORS:

**DATABASE SEARCHED & TIME PERIOD COVERED:**

PubMed – 1/1/2005-2/13/2015

**LANGUAGE:**

English

**SEARCH STRATEGY:**

Rural*[tiab] OR agricultur*[tiab] OR wilderness* OR frontier* OR (native AND reservation*) OR farmer OR farmers OR farming OR farm OR farms OR nonurban* OR "non-urban" OR remote*[tiab] OR outback* OR isolated[tiab] OR "small town" OR "small towns" OR village*[tiab] OR settlement* OR "Rural Population"[Mesh] OR "Rural Nursing"[Mesh] OR "Rural Health Services"[Mesh] OR "Rural Health"[Mesh] OR "Hospitals, Rural"[Mesh]

AND

"Health Personnel"[Mesh] OR physician*[tiab] OR nurses[tiab] OR nursing[tiab] OR hospitalist*[tiab] OR hospital staff*[tiab] OR healthcare professional*[tiab] OR health care professional*[tiab] OR doctor[tiab] OR doctors[tiab] OR health manpower[mh] OR manpower[tiab] OR workforce OR medical staff, hospital OR "family medicine" OR "internal medicine" OR pediatrician* OR pediatric* OR general surgeon* OR psychiatrist* OR geriatric* OR obstetric* OR gynecologis* OR nurse practitioner* OR physician assistant*

AND
choice* OR choos* OR decision* OR decid*

**DATABASE SEARCHED & TIME PERIOD COVERED:**

CINAHL – 1/1/2005-2/13/2015

**LANGUAGE:**

English

**SEARCH STRATEGY:**

Rural* OR agricultur* OR wilderness* OR frontier* OR (native AND reservation*) OR farm OR farmer* OR farmers OR farming OR farm OR farms OR nonurban* OR "non-urban" OR remote* OR isolated OR "small town" OR "small towns" OR village* OR settlement* OR "Rural Population" OR "Rural Health Services" OR "Rural Health" OR "Hospitals, Rural"

AND

"Health Personnel" OR physician* OR nurses OR nursing OR hospitalist* OR hospital staff* OR healthcare professional* OR health care professional* OR doctor OR doctors OR manpower OR workforce OR medical staff, hospital OR "family medicine" OR "internal medicine" OR pediatrician* OR pediatric* OR general surgeon* OR psychiatrist* OR geriatric* OR obstetric* OR gynecologis* OR nurse practitioner* OR physician assistant*

AND

choice* OR choos* OR decision* OR decid*

AND

incentive* OR attract* OR pecuniary OR non-pecuniary OR income OR monetary OR economic* OR financial OR opportunit* OR debt* OR influen*

**DATABASE SEARCHED & TIME PERIOD COVERED:**

PsycINFO – 1/1/2005-2/13/2015

**LANGUAGE:**

English

**SEARCH STRATEGY:**

rural* OR agricultur* OR wilderness* OR frontier* OR (native AND reservation*) OR farm OR farmer* OR farmers OR farming OR farm OR farms OR nonurban* OR "non-urban" OR remote* OR isolated OR "small town" OR "small towns" OR village* OR settlement* OR "Rural Population" OR "Rural Health Services" OR "Rural Health" OR "Hospitals, Rural"

AND

"Health Personnel" OR physician* OR nurses OR nursing OR hospitalist* OR hospital staff* OR healthcare professional* OR health care professional* OR doctor OR doctors OR manpower OR workforce OR medical staff, hospital OR "family medicine" OR "internal medicine" OR pediatrician* OR pediatric* OR general surgeon* OR psychiatrist* OR geriatric* OR obstetric* OR gynecologis* OR nurse practitioner* OR physician assistant*

AND

choice* OR choos* OR decision* OR decid*

AND

incentive* OR attract* OR pecuniary OR non-pecuniary OR income OR monetary OR economic* OR financial OR opportunit* OR debt* OR influen*

**DATABASE SEARCHED & TIME PERIOD COVERED:**

Web of Science– 1/1/2005-2/13/2015

**LANGUAGE:**

English

**SEARCH STRATEGY:**

ts=(Rural* OR agricultur* OR wilderness* OR frontier* OR (native AND reservation*) OR farm OR farmer* OR farmers OR farming OR farm OR farms OR nonurban* OR "non-urban" OR remote* OR isolated OR "small town" OR "small towns" OR village* OR settlement*) AND ts=("Health Personnel" OR physician* OR nurses OR nursing OR hospitalist* OR "hospital staff" OR healthcare professional* OR health care professional* OR doctor OR doctors OR "health manpower" OR "medical staff" OR "family medicine" OR "internal medicine" OR pediatrician* OR pediatric* OR general surgeon* OR psychiatrist* OR geriatric* OR obstetric* OR gynecologis* OR nurse practitioner* OR physician assistant*)

AND

ts=(choice* OR choos* OR decision* OR decid*)

AND

ts=(incentive* OR attract* OR pecuniary OR non-pecuniary OR income OR monetary OR economic* OR financial OR opportunit* OR debt* OR influen*)

**DATABASE SEARCHED & TIME PERIOD COVERED:**

WorldCat– 1/1/2005-2/13/2015

**LANGUAGE:**

English

**SEARCH STRATEGY:**

kw: Rural* OR kw: agricultur* OR kw: wilderness* OR kw: frontier* OR (kw: native AND kw: reservation*) OR kw: farmer OR kw: farmers OR kw: farming OR kw: farm OR kw: farms OR kw: nonurban* OR kw: non-urban OR kw: remote* OR kw: isolate* OR kw: small w town OR kw: small w towns OR kw: village* OR kw: settlement*

AND

kw: Health w Personnel OR kw: physician* OR kw: nurses OR kw: nursing OR kw: hospitalist* OR (kw: hospital and kw: staff) OR (kw: healthcare and kw: professional*) OR ((kw: health and kw: care and kw: professional*) OR kw: doctor) OR kw: doctors OR kw: family w medicine OR kw: internal w medicine OR kw: pediatrician* OR kw: pediatric* OR (kw: general and kw: surgeon*) OR kw: psychiatrist* OR kw: geriatric* OR kw: obstetric* OR kw: gynecologis* OR (kw: nurse and kw: practitioner*) OR (kw: physician and kw: assistant*)

AND

kw: choice* OR kw: choos* OR kw: decision* OR kw: decid*

AND

kw: incentive* OR kw: attract* OR kw: pecuniary OR kw: non-pecuniary OR kw: income OR kw: monetary OR kw: economic* OR kw: financial OR kw: opportunit* OR kw: debt* OR kw: influen*

FILTERED TO NON-JUVENILE, NON-FICTION

**==========================================================================**

#### RECRUITMENT AND RETENTION:

**DATABASE SEARCHED & TIME PERIOD COVERED:**

PubMed– 1/1/2005-2/16/2015

**LANGUAGE:**

English

**SEARCH STRATEGY:**

Rural*[tiab] OR agricultur*[tiab] OR wilderness* OR frontier* OR (native AND reservation*) OR farmer OR farmers OR farming OR farm OR farms OR nonurban* OR "non-urban" OR remote*[tiab] OR outback* OR isolated[tiab] OR "small town" OR "small towns" OR village*[tiab] OR settlement* OR "Rural Population"[Mesh] OR "Rural Nursing"[Mesh] OR "Rural Health Services"[Mesh] OR "Rural Health"[Mesh] OR "Hospitals, Rural"[Mesh]

AND

"Health Personnel"[Mesh] OR physician*[tiab] OR nurses[tiab] OR nursing[tiab] OR hospitalist*[tiab] OR hospital staff*[tiab] OR healthcare professional*[tiab] OR health care professional*[tiab] OR doctor[tiab] OR doctors[tiab] OR health manpower[mh] OR manpower[tiab] OR workforce OR medical staff, hospital OR "family medicine" OR "internal medicine" OR pediatrician* OR pediatric* OR general surgeon* OR psychiatrist* OR geriatric* OR obstetric* OR gynecologis* OR nurse practitioner* OR physician assistant* OR medical staff, hospital

AND

recruit*[ti] OR retention[ti] OR retain*[ti] OR "Personnel Selection"[Mesh] OR turnover[ti] OR turn over*[ti] OR burnout[tiab] OR "burned out"[tiab]

OR

rural health services/manpower

**DATABASE SEARCHED & TIME PERIOD COVERED:**

CINAHL– 1/1/2005-2/17/2015

**LANGUAGE:**

English

**SEARCH STRATEGY:**

Rural* OR agricultur* OR wilderness* OR frontier* OR (native AND reservation*) OR farm OR farmer* OR farmers OR farming OR farm OR farms OR nonurban* OR "non-urban" OR remote* OR isolated OR "small town" OR "small towns" OR village* OR settlement* OR "Rural Population" OR "Rural Health Services" OR "Rural Health" OR "Hospitals, Rural"

AND

"Health Personnel" OR physician* OR nurses OR nursing OR hospitalist* OR hospital staff* OR healthcare professional* OR health care professional* OR doctor OR doctors OR manpower OR workforce OR medical staff, hospital OR "family medicine" OR "internal medicine" OR pediatrician* OR pediatric* OR general surgeon* OR psychiatrist* OR geriatric* OR obstetric* OR gynecologis* OR nurse practitioner* OR physician assistant*

AND

recruit* OR retention OR retain* OR "Personnel Selection" OR turnover OR turn over* OR burnout OR "burned out"

**OR**

personnel retention evaluation

AND

rural areas

**DATABASE SEARCHED & TIME PERIOD COVERED:**

Web of Science– 1/1/2005-2/17/2015

**LANGUAGE:**

English

**SEARCH STRATEGY:**

ts=(Rural* OR agricultur* OR wilderness* OR frontier* OR (native AND reservation*) OR farm OR farmer* OR farmers OR farming OR farm OR farms OR nonurban* OR "non-urban" OR remote* OR isolated OR "small town" OR "small towns" OR village* OR settlement*)

AND

ts=("Health Personnel" OR physician* OR nurses OR nursing OR hospitalist* OR "hospital staff" OR healthcare professional* OR health care professional* OR doctor OR doctors OR "health manpower" OR "medical staff" OR "family medicine" OR "internal medicine" OR pediatrician* OR pediatric* OR general surgeon* OR psychiatrist* OR geriatric* OR obstetric* OR gynecologis* OR nurse practitioner* OR physician assistant*)

AND

ts=(recruit* OR retention OR retain* OR "Personnel Selection" OR turnover OR turn over* OR burnout OR "burned out")

**DATABASE SEARCHED & TIME PERIOD COVERED:**

WorldCat– 1/1/2005-2/18/2015

**LANGUAGE:**

English

**SEARCH STRATEGY:**

kw: Rural* OR kw: agricultur* OR kw: wilderness* OR kw: frontier* OR (kw: native AND kw: reservation*) OR kw: farmer OR kw: farmers OR kw: farming OR kw: farm OR kw: farms OR kw: nonurban* OR kw: non-urban OR kw: remote* OR kw: isolate* OR kw: small w town OR kw: small w towns OR kw: village* OR kw: settlement*

AND

kw: Health w Personnel OR kw: physician* OR kw: nurses OR kw: nurse OR kw: nursing OR kw: hospitalist* OR (kw: hospital and kw: staff) OR (kw: healthcare and kw: professional*) OR (kw: health and kw: care and kw: professional*) OR kw: doctor OR kw: doctors OR kw: family w medicine OR kw: internal w medicine OR kw: pediatrician* OR kw: pediatric* OR (kw: general and kw: surgeon*) OR kw: psychiatrist* OR kw: geriatric* OR kw: obstetric* OR kw: gynecologis* OR (kw: nurse and kw: practitioner*) OR (kw: physician and kw: assistant*)

AND

kw: recruit* OR kw: retention OR kw: retain* OR kw: Personnel w Selection OR kw: turnover OR (kw: turn and kw: over*) OR kw: burnout OR kw: burned w out

**==========================================================================**

#### EDUCATION AND TRAINING

**DATABASE SEARCHED & TIME PERIOD COVERED:**

PubMed– 1/1/2005-2/20/2015

**LANGUAGE:**

English

**SEARCH STRATEGY:**

Rural*[tiab] OR agricultur*[tiab] OR wilderness* OR frontier* OR (native AND reservation*) OR farmer OR farmers OR farming OR farm OR farms OR nonurban* OR "non-urban" OR remote*[tiab] OR outback* OR isolated[tiab] OR "small town" OR "small towns" OR village*[tiab] OR settlement* OR "Rural Population"[Mesh] OR "Rural Nursing"[Mesh] OR "Rural Health Services"[Mesh] OR "Rural Health"[Mesh] OR "Hospitals, Rural"[Mesh]

AND

"Health Personnel"[Mesh] OR physician*[tiab] OR nurses[tiab] OR nursing[tiab] OR hospitalist*[tiab] OR hospital staff*[tiab] OR healthcare professional*[tiab] OR health care professional*[tiab] OR doctor[tiab] OR doctors[tiab] OR health manpower[mh] OR manpower[tiab] OR workforce OR medical staff, hospital OR "family medicine" OR "internal medicine" OR pediatrician* OR pediatric* OR general surgeon* OR psychiatrist* OR geriatric* OR obstetric* OR gynecologis* OR nurse practitioner* OR physician assistant*

AND

"Schools, Health Occupations"[Mesh] OR "Students, Health Occupations"[Mesh] OR internship and residency[mh] OR graduate[tiab] OR post-graduate[tiab] OR postgraduate[tiab] OR college[tiab] OR training OR train[ti] OR educat*[tiab] OR medical education OR education, professional

AND

program OR programs[tiab] OR programme*[tiab] OR project[tiab] OR projects[tiab] OR effort[tiab] OR efforts[tiab] OR evaluat*[tiab] OR improv*[tiab] OR success*[tiab] OR efficacy OR survey* OR questionnaire*

**DATABASE SEARCHED & TIME PERIOD COVERED:**

CINAHL– 1/1/2005-2/20/2015

**LANGUAGE:**

English

**SEARCH STRATEGY:**

Rural* OR agricultur* OR wilderness* OR frontier* OR (native AND reservation*) OR farm OR farmer* OR farmers OR farming OR farm OR farms OR nonurban* OR "non-urban" OR remote* OR isolated OR "small town" OR "small towns" OR village* OR settlement* OR "Rural Population" OR "Rural Health Services" OR "Rural Health" OR "Hospitals, Rural"

AND

"Health Personnel" OR physician* OR nurses OR nursing OR hospitalist* OR hospital staff* OR healthcare professional* OR health care professional* OR doctor OR doctors OR manpower OR workforce OR medical staff, hospital OR "family medicine" OR "internal medicine" OR pediatrician* OR pediatric* OR general surgeon* OR psychiatrist* OR geriatric* OR obstetric* OR gynecologis* OR nurse practitioner* OR physician assistant*

AND

training OR train OR trained OR educat* OR graduat* OR post-graduate OR postgraduate OR college

AND

NON-MEDLINE

AND
GEOGRAPHY – USA

**DATABASE SEARCHED & TIME PERIOD COVERED:**

ERIC– 1/1/2005-2/20/2015

**LANGUAGE:**

English

**SEARCH STRATEGY:**

Rural* OR agricultur* OR wilderness* OR frontier* OR (native AND reservation*) OR farm OR farmer* OR farmers OR farming OR farm OR farms OR nonurban* OR "non-urban" OR remote* OR isolated OR "small town" OR "small towns" OR village* OR settlement* OR "Rural Population" OR "Rural Health Services" OR "Rural Health" OR "Hospitals, Rural"

AND

"Health Personnel" OR physician* OR nurses OR nursing OR hospitalist* OR hospital staff* OR healthcare professional* OR health care professional* OR doctor OR doctors OR manpower OR workforce OR medical staff, hospital OR "family medicine" OR "internal medicine" OR pediatrician* OR pediatric* OR general surgeon* OR psychiatrist* OR geriatric* OR obstetric* OR gynecologis* OR nurse practitioner* OR physician assistant*

AND

training OR train OR trained OR educat* OR graduat* OR post-graduate OR postgraduate OR college

**DATABASE SEARCHED & TIME PERIOD COVERED:**

Web of Science– 1/1/2005-2/20/2015

**LANGUAGE:**

English

**SEARCH STRATEGY:**

ts=(Rural* OR agricultur* OR wilderness* OR frontier* OR (native AND reservation*) OR farm OR farmer* OR farmers OR farming OR farm OR farms OR nonurban* OR "non-urban" OR remote* OR isolated OR "small town" OR "small towns" OR village* OR settlement*)

AND

ts=("Health Personnel" OR physician* OR nurses OR nursing OR hospitalist* OR "hospital staff" OR healthcare professional* OR health care professional* OR doctor OR doctors OR "health manpower" OR "medical staff" OR "family medicine" OR "internal medicine" OR pediatrician* OR pediatric* OR general surgeon* OR psychiatrist* OR geriatric* OR obstetric* OR gynecologis* OR nurse practitioner* OR physician assistant*)

AND

ts=(training OR train OR trained OR educat* OR graduat* OR post-graduate OR postgraduate OR college)

AND

ts=(program OR programs OR project OR projects OR effort OR efforts OR evaluat* OR improv* OR success* OR efficacy OR survey* OR questionnaire*)

**==========================================================================**

#### GENERAL:

**DATABASE SEARCHED & TIME PERIOD COVERED:**

Grey Literature Report– 1/1/2005-1/16/2015

**SEARCH STRATEGY:**

rural

## Reasons for exclusion

### Not a participant group of interest

1. Amerson, R.M.G.L.S. and a. thesis. *Understanding the occupational choices of rural white southern males*. [Book; Computer File] 2012; 1 online resource (208 p.) Dissertation: Ed. D.; Georgia Southern University; 2012.]. Available from: Host: http://digitalcommons.georgiasouthern.edu/etd/570/.

2. Bradbury, G.B., *Retention of staff nurses in a rural hospital setting in the eastern part of North Carolina: The impact of leadership style.* Capella University, 2013. **152**.

3. Bratt, M.M., *Retaining the next generation of nurses: the Wisconsin nurse residency program provides a continuum of support.* J Contin Educ Nurs, 2009. **40**(9): p. 416-25.

4. Cramer, M., et al., *Comparative analysis of urban and rural nursing workforce shortages in Nebraska hospitals.* Policy Polit Nurs Pract, 2006. **7**(4): p. 248-60.

5. Crump, W.J., et al., *A Rural Pathways Program for High School Students: Reinforcing a Sense of Place.* Family Medicine, 2014. **46**(9): p. 713-717.

6. DeOnna, J., *Rural healthcare manager academy: a best practice in Pennsylvania workforce development.* Pa Nurse, 2008. **63**(4): p. 14-6.

7. deValpine, M.G., *Extreme nursing: a qualitative assessment of nurse retention in a remote setting.* Rural Remote Health, 2014. **14**(3): p. 2859.

8. Freeman, V.A., R.T. Slifkin, and P.D. Patterson, *Recruitment and retention in rural and urban EMS: results from a national survey of local EMS directors.* J Public Health Manag Pract, 2009. **15**(3): p. 246-52.

9. Fu, M.C., et al., *Longitudinal urban-rural discrepancies in the US orthopaedic surgeon workforce.* Clin Orthop Relat Res, 2013. **471**(10): p. 3074-81.

10. Gifford, V.M., *Factors that contribute to rural provider retention, service utilization, and engagement in mentorship by cultural experts*. 2012. p. xiii, 208 leaves ; 28 cm. Dissertation: Thesis (Ph.D.)--University of Alaska Anchorage, 2012.; Thesis (Ph.D.)--University of Alaska Fairbanks, 2012.

11. Gossler, H.E. *Reducing the rural physician shortage in the Pacific Northwest incentivizing rural physicians through financial reimbursement*. [Internet Resource; Computer File] 2011 [cited 2012 April 13]; 1 online resource (vi, 37 p.) Dissertation: Thesis (M.P.A.)--Washington State University, December 2011.; M.P.A.; Washington State University; 2011.]. Available from: http://www.dissertations.wsu.edu/Thesis/Fall2011/h_gossler_112211.pdf

12. Grauer, J.N. and M.C. Fu, *Editor's spotlight/take 5: longitudinal urban-rural discrepancies in the US orthopaedic surgeon workforce. Interview by Seth S. Leopold.* Clin Orthop Relat Res, 2013. **471**(10): p. 3071-3.

13. Greenwood, J.E., *The impact of geography, training, and experience on scope of practice among Certified Registered Nurse Anesthetists.* Virginia Commonwealth University, 2014. **176**.

14. Hauenstein, E.J., et al., *A Model to Develop Nurse Leaders for Rural Practice.* Journal of Professional Nursing, 2014. **30**(6): p. 463-73.

15. Herrmann, J.A. and R.C. Hershow, *One Medicine, one university: The DVM/MPH program at the University of Illinois.* Journal of Veterinary Medical Education, 2008. **35**(2): p. 194-198.

16. Hughes, J., et al., *Disparities in how parents are learning about the human papillomavirus vaccine.* Cancer Epidemiol Biomarkers Prev, 2009. **18**(2): p. 363-72.

17. Kruger, G.J., *Perceived factors affecting nurse recruitment in northwest Minnesota.* University of North Dakota, 2009. **102**.

18. Meyer, D., et al., *Recruiting and retaining mental health professionals to rural communities: an interdisciplinary course in Appalachia.* J Rural Health, 2005. **21**(1): p. 86-91.

19. Molanari, D.L., A. Jaiswal, and T. Hollinger-Forrest, *Rural Nurses: Lifestyle Preferences and Education Perceptions.* Online Journal of Rural Nursing & Health Care, 2011. **11**(2): p. 16-26.

20. Murray, M.F., et al., *The rural pipeline: building a strong nursing workforce through academic and service partnerships.* Nurs Clin North Am, 2011. **46**(1): p. 107-21.

21. Nayar, P., et al., *Preventable hospitalizations: does rurality or non-physician clinician supply matter?* J Community Health, 2012. **37**(2): p. 487-94.

22. Nelson, W., et al., *New Hampshire critical access hospitals: CEOs' report on ethical challenges.* J Healthc Manag, 2009. **54**(4): p. 273-83; discussion 283-4.

23. Pruthi, R.S., et al., *Recent trends in the urology workforce in the United States.* Urology, 2013. **82**(5): p. 987-93.

24. Rhyne, R.L., et al., *Interdisciplinary health education and career choice in rural and underserved areas.* Medical Education, 2006. **40**(6): p. 504-513.

25. Rohatinsky, N.K. and S. Jahner, *Supporting nurses' transition to rural healthcare environments through mentorship.* Rural Remote Health, 2016. **16**(1): p. 3637.

26. Skillman, S.M., *Changes in the rural registered nurse workforce from 1980 to 2004*. Final report. 2007, Seattle: WWAMI Center for Health Workforce Studies, University of Washington, School of Medicine, Dept. of Family Medicine. 16 p.

27. Slagle, D.R. *Rural Versus Urban: Tennessee Health Administratorsâ Strategies on Recruitment and Retention for Allied Health Professionals*. [Internet Resource] 2010; Available from: http://etd-submit.etsu.edu/etd/theses/available/etd-0702110-121920/

28. Sullivan, D. and L. Rohlfsen, *Professional Politics and the Challenge of Anesthesia Availability in Rural Hospitals*, in *Inequalities and Disparities in Health Care and Health: Concerns of Patients, Providers and Insurers*. 2007, Emerald Group Publishing Limited: Bingley. p. 149-167.

29. Thaker, S.I., et al., *Service-linked scholarships, loans, and loan repayment programs for nurses in the Southeast.* Journal of Professional Nursing, 2008. **24**(2): p. 122-30.

30. Trepanier, A., et al., *Factors associated with intended and effective settlement of nursing students and newly graduated nurses in a rural setting after graduation: a mixed-methods review.* Int J Nurs Stud, 2013. **50**(3): p. 314-25.

31. Wachtel, R.E. and F. Dexter, *Training rotations at hospitals as a recruitment tool for Certified Registered Nurse Anesthetists.* Aana j, 2012. **80**(4 Suppl): p. S45-8.

32. Weichel, D., *Orthopedic surgery in rural American hospitals: a survey of rural hospital administrators.* J Rural Health, 2012. **28**(2): p. 137-41.

### Not a training program of interest or exposure factor predicting practicing in rural care

1. *Bringing health care to the heartland : an evaluation of Minnesota's loan forgiveness programs for select health care occupations*. 2007, [Minnesota]: Minnesota Dept. of Health, Office of Rural Health and Primary Care. 47 p.

2. Anderson, D.M., et al., *Increasing the medical school applicant pool: a key to training more rural physicians.* J Ky Med Assoc, 2009. **107**(9): p. 355-60.

3. Branch, K., *Alaska’s Health Workforce Vacancy Study: 2012 Findings Report. August 2014*. 2014, Alaska Center for Rural Health, Alaska’s Area Health Education Center, University of Alaska Anchorage: Anchorage, AK.

4. Camargo, C.A., Jr., et al., *Assessment of emergency physician workforce needs in the United States, 2005.* Acad Emerg Med, 2008. **15**(12): p. 1317-20.

5. Casey, M.M., et al., *The Use of Hospitalists by Small Rural Hospitals: Results of a National Survey.* Medical Care Research and Review, 2014. **71**(4): p. 356-366.

6. Ghosh, D., et al., *Geospatial study of psychiatric mental health-advanced practice registered nurses (PMH-APRNs) in the United States.* Psychiatr Serv, 2011. **62**(12): p. 1506-9.

7. Hendryx, M., *Mental health professional shortage areas in rural Appalachia.* Journal of Rural Health, 2008. **24**(2): p. 179-182.

8. Kahn, T.R., A. Hagopian, and K. Johnson, *Retention of J-1 visa waiver program physicians in Washington State's health professional shortage areas.* Acad Med, 2010. **85**(4): p. 614-21.

9. Kippenbrock, T., et al., *The Southern states: NPs made an impact in rural and healthcare shortage areas.* Journal of the American Association of Nurse Practitioners, 2015. **27**(12): p. 707-713.

10. Maizel, S.E., *Maryland's surgical workforce-2007: an in-depth analysis and implications for the future.* J Am Coll Surg, 2009. **208**(3): p. 454-61.

11. Rayburn, W.F., et al., *Distribution of American Congress of Obstetricians and Gynecologists Fellows and Junior Fellows in Practice in the United States.* Obstetrics and Gynecology, 2012. **119**(5): p. 1017-1022.

12. Reese, V.F., et al., *Residency footprints: assessing the impact of training programs on the local physician workforce and communities.* Fam Med, 2008. **40**(5): p. 339-44.

13. Rodney, W.M., et al., *OB Fellowship Outcomes 1992-2010: Where Do They Go, Who Stops Delivering, and Why?* Family Medicine, 2010. **42**(10): p. 712-716.

14. Rosenblatt, R.A., et al., *The Future of Family Medicine and Implications for Rural Primary Care Physician Supply. Final Report #125, August 2010.* 2010, WWAMI Rural Health Research Center, University of Washington School of Medicine Department of Family Medicine.: Seattle, WA.

15. Singh, R., et al., *Sustainable Rural Telehealth Innovation: A Public Health Case Study.* Health Services Research, 2010. **45**(4): p. 985-1004.

16. Stewart, R.M., et al., *The general surgery workforce shortage is worse when assessed at county level.* American Journal of Surgery, 2013. **206**(6): p. 1016-1022.

17. Thomas, K.C., et al., *County-level estimates of mental health professional shortage in the United States.* Psychiatr Serv, 2009. **60**(10): p. 1323-8.

18. Watanabe-Galloway, S., et al., *Recruitment and retention of mental health care providers in rural Nebraska: perceptions of providers and administrators.* Rural Remote Health, 2015. **15**(4): p. 3392.

19. Wheeler, D.L.L.C.E. *Oklahoma State University Center for Rural Health report to the Senate interim study on comprehensive assessment of healthcare workforce issues*. [Internet Resource; Computer File] 2009; 1 online resource (20 p. : 1.41 MB) : col. ill., col. maps.]. Available from: http://digitalprairie.ok.gov/u?/stgovpub,19719

20. Wheeler, M., et al., *Dr. Who? Providing stability to recruiting and retaining health care professionals in West Virginia.* W V Med J, 2013. **109**(4): p. 62-4.

21. Williams, T.E., Jr., B. Satiani, and E.C. Ellison, *A comparison of future recruitment needs in urban and rural hospitals: the rural imperative.* Surgery, 2011. **150**(4): p. 617-25.

22. Wilson, E.A., E.T. Whitler, and L.M. Asher, *Meeting the Need of a Rural State for Primary Care Physicians: A Health Care Reform Challenge.* Journal of Public Health Management and Practice, 2011. **17**(2): p. 147-153.

### Not a study design of interest

1. *Shortages of rural generalist physicians may be due to poor recruitment rather than retention problems.* AHRQ Research Activities, 2005. **293**: p. 19.

2. Barr, P., *Looking for an oasis: large sections of rural America continue to suffer from a drought of general surgeons.* Mod Healthc, 2012. **42**(13): p. 28-31.

3. Basco, W.T. and M.E. Rimsza, *Pediatrician workforce policy statement.* Pediatrics, 2013. **132**(2): p. 390-7.

4. Behringer, B. and G.H. Friedell, *Appalachia: where place matters in health.* Prev Chronic Dis, 2006. **3**(4): p. A113.

5. Bellinger, N., *Predictors of primary care physicians practicing in medically underserved and rural areas of Indiana*, in *Variation: IUPUI theses and dissertations.* 2009.

6. Berens, D., *Recruitment or retention: J-1 visa lessons.* Wmj, 2006. **105**(7): p. 11.

7. Brooks, K.D., et al., *Primary care in Minnesota: an academic health center's perspective.* Minn Med, 2008. **91**(5): p. 40-4.

8. Calman, N.S., et al., *New York State physicians: characteristics and distribution in health professional shortage areas.* J Urban Health, 2007. **84**(2): p. 307-9.

9. Casaletto, J.J., et al., *Emergency Medicine Rural Rotations: A Program Director's Guide.* Annals of Emergency Medicine, 2013. **61**(5): p. 578-83.

10. Casey, B.R., et al., *Rural Kentucky's physician shortage: strategies for producing, recruiting, and retaining primary care providers within a medically underserved region.* J Ky Med Assoc, 2005. **103**(10): p. 505-13.

11. Chen, F. and L.G. Hart, *Another look at rural health.* Health Aff (Millwood), 2006. **25**(2): p. 570-1; author reply 571.

12. Chewning, L.H. and J. Spade, *Rural hospitals and rural physicians: understanding the physician workforce challenges that affect rural communities and providers.* N C Med J, 2007. **68**(3): p. 191-3.

13. Click, I.A., *Practice characteristics of graduates of east tennessee state university quillen college of medicine: Factors related to career choices in primary care.* Dissertation Abstracts International Section A: Humanities and Social Sciences, 2014. **74**(9-A(E)).

14. Cofer, J.B. and R.P. Burns, *The developing crisis in the national general surgery workforce.* J Am Coll Surg, 2008. **206**(5): p. 790-5; discussion 795-7.

15. Cofer, J.B., et al., *General Surgery at Rural Tennessee Hospitals: A Survey of Rural Tennessee Hospital Administrators.* American Surgeon, 2011. **77**(7): p. 820-825.

16. Colgan, R., et al., *University of Maryland School of Medicine increases medical student education in primary care.* Md Med, 2011. **12**(4): p. 26-7, 30.

17. Collins, S., *Primary care shortages: strengthening this sector is urgently needed, now and in preparation for healthcare reform.* Am Health Drug Benefits, 2012. **5**(1): p. 40-7.

18. Danto, L.A., *National rural health service.* Bull Am Coll Surg, 2010. **95**(8): p. 43.

19. Edgerly, D., *Rural Colorado care. West Routt Fire Protection District provides BLS & ALS.* Jems, 2013. **38**(6): p. 23.

20. Emery, J.G., *Comment on: Medical students on long-term regional and rural placements: what is the financial cost to supervisors?* Rural Remote Health, 2012. **12**: p. 2247; discussion 2287.

21. Farmer, D., *Rural surgery is global surgery: seeking solutions to the growing surgical workforce crisis.* JAMA Surg, 2013. **148**(9): p. 821-2.

22. Farrell, P.M., *Plan to address physician shortage requires proper support.* Wmj, 2005. **104**(6): p. 73-4.

23. Ferguson, W.J., et al., *Family Medicine Residency Characteristics Associated With Practice in a Health Professions Shortage Area.* Family Medicine, 2009. **41**(6): p. 405-410.

24. Galloro, V., *Hard recruitment for smaller markets. Report shows importance of physicians to rural hospitals' financial health.* Mod Healthc, 2008. **38**(22): p. 30-1.

25. Garpestad, B., *Funding for frontier home care: an anatomy of Montana's rural health care.* Caring, 2009. **28**(4): p. 38-41.

26. Glasser, M., K. Peters, and M. Macdowell, *Rural Illinois hospital chief executive officers' perceptions of provider shortages and issues in rural recruitment and retention.* J Rural Health, 2006. **22**(1): p. 59-62.

27. Greenhill, J. and A.N. Poncelet, *Transformative learning through longitudinal integrated clerkships.* Med Educ, 2013. **47**(4): p. 336-9.

28. Gunn, J., *Minnesota's rural health workforce shortages.* Minn Med, 2013. **96**(12): p. 41-5.

29. Halaas, G.W., *The Rural Physician Associate Program: new directions in education for competency.* Rural Remote Health, 2005. **5**(4): p. 359.

30. Handel, D.A. and J.R. Hedges, *Improving rural access to emergency physicians.* Acad Emerg Med, 2007. **14**(6): p. 562-5.

31. Heady, H.R., D. Phillips, and E. Briggs, *Rural healthcare: how to recruit and retain health professionals.* J Med Pract Manage, 2006. **21**(4): p. 219-24.

32. Hooker, R.S., D.J. Klocko, and G.L. Larkin, *Physician Assistants in Emergency Medicine: The Impact of Their Role.* Academic Emergency Medicine, 2011. **18**(1): p. 72-77.

33. Huff, C., *Give docs a reason to set up shop.* H&HN: Hospitals & Health Networks, 2012. **86**(2): p. 20.

34. Huff, C., *You got the docs, now keep them! 5 strategies for rural hospitals to help retain their hard-won physicians.* Hosp Health Netw, 2014. **88**(4): p. 32-5, 2.

35. Huffstutter, P.J., *Rural surgeons--we must grow our own.* Bull Am Coll Surg, 2010. **95**(4): p. 16-8; discussion 19.

36. Johna, S., *The rural surgeon: an endangered species.* World J Surg, 2006. **30**(3): p. 267-8.

37. Johnson, C.A. and B.E. Johnson, *Family medicine practice in rural any-country.* Fam Med, 2008. **40**(1): p. 55-6.

38. Kane, K.Y., et al., *Summer in the country: changes in medical students' perceptions following an innovative rural community experience.* Acad Med, 2013. **88**(8): p. 1157-63.

39. Kirschling, J.M., L. Horvey-McPherson, and D. Curley, *Maine's nursing workforce legislation: Lessons from a rural state.* Nursing Outlook, 2008. **56**(2): p. 63-69.

40. Kutscher, B., *The rural route. Hospitals in underserved areas taking different roads to recruit, retain physicians.* Mod Healthc, 2013. **43**(18): p. 30-1.

41. Lasher, W.F. and S.B. Silverman. *Relationship between residency training and practice location in primary care residency programs in Texas*. [Internet Resource; Archival Material] 2008; Available from: http://hdl.handle.net/2152/3687

42. Lauver, L.S., et al., *Kids Into Health Careers: A Rural Initiative.* Journal of Rural Health, 2011. **27**(1): p. 114-121.

43. Longo, W.E., et al., *Early specialization in surgery: the new frontier.* Yale J Biol Med, 2008. **81**(4): p. 187-91.

44. Lynge, D.C. and E.H. Larson, *Workforce issues in rural surgery.* Surg Clin North Am, 2009. **89**(6): p. 1285-91, vii.

45. Mantone, J., *Protective moves. Caps may lure docs to rural communities: study.* Mod Healthc, 2005. **35**(23): p. 8-9.

46. Margolis, S.A., *Retaining rural medical practitioners: time for a new paradigm?* Rural Remote Health, 2005. **5**(1): p. 393.

47. Margolis, S.A., *Letter to the editor Retaining rural medical practitioners: time for a new paradigm?* Rural & Remote Health, 2005. **5**(1).

48. McHugh, G. and G. Stewart, *Regional and rural specialists.* Anaesth Intensive Care, 2010. **38**(4): p. 778-9.

49. Mobley, J. and M.A. Zuniga, *Training primary care physicians for local health authority duties in Texas.* Am J Public Health, 2012. **102**(7): p. e21-6.

50. Morley, C.P., *Supporting Physicians Who Work in Challenging Contexts: A Role for the Academic Health Center.* Journal of the American Board of Family Medicine, 2012. **25**(6): p. 756-758.

51. Nelson, B.V. and G.E. Talboy, Jr., *Acute care surgery: redefining the general surgeon.* Mo Med, 2010. **107**(5): p. 313-5.

52. Nocella, I.C., *Recruitment of family physicians into rural California : predictors and possibilities*. 2005. p. x, 144 leaves.

53. Nusbaum, N., *Commentary: Physician retirement and physician shortages.* Journal of Community Health, 2009. **34**(5): p. 353-6.

54. O'Brien, M.L., *Perception of rural caregiving*. 2008. p. vii, 70 leaves ; 28 cm Dissertation: Thesis (M.S.)--University of Vermont, 2008.

55. Oklahoma. Physician Manpower Training, C. *Oklahoma Medical Loan Repayment Program*. [Internet Resource; Archival Material] 2012; Medium: Fact Sheet;]. Available from: http://digitalprairie.ok.gov/cdm/ref/collection/stgovpub/id/229002

56. Page, S. and H. Birden, *Twelve tips on rural medical placements: what has worked to make them successful.* Med Teach, 2008. **30**(6): p. 592-6.

57. Penn, C.L., *Now hiring! Arkansas, like the nation, faces imminent health care worker shortages.* J Ark Med Soc, 2006. **103**(4): p. 84-7.

58. Potter, A.J., et al., *Effect of tele-emergency services on recruitment and retention of US rural physicians.* Rural Remote Health, 2014. **14**(3): p. 2787.

59. Prengaman, M.P., et al., *Development of the Nursing Community Apgar Questionnaire (NCAQ): a rural nurse recruitment and retention tool.* Rural and Remote Health, 2014. **14**(1).

60. Quinn, K.J., *Experiences influencing physician rural practice and retention: A phenomenological study.* Dissertation Abstracts International Section A: Humanities and Social Sciences, 2012. **72**(8-A): p. 2697.

61. Rabinowitz, H.K., *Addressing physician specialty maldistribution.* Jama, 2009. **302**(12): p. 1270; author reply 1270.

62. Ricketts, T.C., 3rd and E. Walker, *Health reform and workforce: the North Carolina connection.* N C Med J, 2010. **71**(3): p. 250-3.

63. Robertson, J.E., Jr., et al., *Strategies for increasing the physician workforce: the Oregon model for expansion.* Acad Med, 2007. **82**(12): p. 1158-62.

64. Rosenblatt, R.A., *Commentary: do medical schools have a responsibility to train physicians to meet the needs of the public? The case of persistent rural physician shortages.* Acad Med, 2010. **85**(4): p. 572-4.

65. Rourke, J., *How can medical schools contribute to the education, recruitment and retention of rural physicians in their region?* Bull World Health Organ, 2010. **88**(5): p. 395-6.

66. Schein, M., *Editorial comment: Surgery in rural America.* World J Surg, 2006. **30**(3): p. 271-2.

67. Scutchfield, F.D., *Increasing the medical school application pool: a key to training more rural physicians.* J Ky Med Assoc, 2009. **107**(11): p. 464.

68. Sharp, D.B., *Factors related to the recruitment and retention of nurse practitioners in rural areas.* University of Texas at El Paso, 2010. **108**.

69. Sheldon, G.F., *Surgical practice in rural areas. Introduction.* Surg Clin North Am, 2009. **89**(6): p. xvii-xix.

70. Smith, R.J. *Frontier residents' perception of health care access*. [Internet Resource] 2008; vii, 75 leaves : ill. Dissertation: Thesis (M Nursing)--Montana State University--Bozeman, 2008.]. Available from: http://etd.lib.montana.edu/etd/2008/smith/SmithR0508.pdf.

71. Stempniak, M., *Rural hospitals band together to lure physicians.* H&HN: Hospitals & Health Networks, 2012. **86**(12): p. 18-9.

72. Steptoe, A.P., et al., *Factors In Recruiting and Retaining Emergency Physicians to Rural Emergency Departments.* Annals of Emergency Medicine, 2010. **56**(3): p. S72-S72.

73. Stevens, C.B., *The dilemma of the rural psychiatrist.* Journal of Psychiatric Practice, 2012. **18**(5): p. 369-72.

74. Sumaya, C.V., C.M. Opara, and D.V. Espino, *The Geriatrician and Geriatric Psychiatrist Workforce in Texas: Characteristics, Challenges, and Policy Implications.* Journal of Aging and Health, 2013. **25**(6): p. 1050-1064.

75. Szabo, J., *Rural hospitals look near and far for allied health professionals.* Hosp Health Netw, 2011. **85**(5): p. 30-2, 2.

76. Thrall, T.H., *Physician recruitment. Encouraging med students to return to rural areas.* Hosp Health Netw, 2007. **81**(6): p. 20.

77. Thrall, T.H., *Physician recruitment. Garrison Keillor: doctor magnet.* Hosp Health Netw, 2008. **82**(2): p. 24.

78. Tong, S.T.C., et al., *Proportion of Family Physicians Providing Maternity Care Continues to Decline.* Journal of the American Board of Family Medicine, 2012. **25**(3): p. 270-271.

79. Turlington, A.R., *Barriers to rural mental health care: Perspectives from those who live there.* Dissertation Abstracts International: Section B: The Sciences and Engineering, 2008. **69**(1-B): p. 703.

80. Wakefield, M., et al., *Policy brief: North Dakota Health Care Workforce: planning together to meet future health care needs.* Prairie Rose, 2007. **76**(2): p. 9-13.

81. Welch, J.P., *Whither goest general surgery?* Arch Surg, 2008. **143**(5): p. 444-50.

82. Westergaard, R.P., et al., *Provider Workforce Assessment in a Rural Hepatitis C Epidemic: Implications for Scale-up of Antiviral Therapy.* J Prim Care Community Health, 2014.

83. Wheat, J.R., et al., *Medical Education to Improve Rural Population Health: A Chain of Evidence From Alabama.* J Rural Health, 2015. **31**(4): p. 354-64.

84. Zastrow, A.W., *Dilemma of general surgery recruitment in rural America.* World J Surg, 2006. **30**(3): p. 269-70.

85. Zigmond, J., *Help wanted. Benefits include an idyllic rural setting, a friendly community and some assistance paying off those hefty medical school loans.* Mod Healthc, 2006. **36**(31): p. 30-1.

86. Zuckerman, R.S., *Rural surgery and surgical education.* Surg Endosc, 2008. **22**(7): p. 1592.

87. Zukowsky, K., et al., *Implementing an MSN nursing program at a distance through an urban-rural partnership.* Adv Neonatal Care, 2011. **11**(2): p. 114-8.

### Not reporting on an outcome of interest

1. Alicata, D., et al., *Telemental Health Training, Team Building, and Workforce Development in Cultural Context: The Hawaii Experience.* Journal of Child and Adolescent Psychopharmacology, 2016. **26**(3): p. 260-265.

2. Allen, J.E.t., et al., *Residents' Willingness-to-Pay for Attributes of Rural Health Care Facilities.* J Rural Health, 2015. **31**(1): p. 7-18.

3. Allen, S.M., et al., *Challenges and opportunities in building a sustainable rural primary care workforce in alignment with the Affordable Care Act: the WWAMI program as a case study.* Acad Med, 2013. **88**(12): p. 1862-9.

4. Arora, S., et al., *Partnering Urban Academic Medical Centers And Rural Primary Care Clinicians To Provide Complex Chronic Disease Care.* Health Affairs, 2011. **30**(6): p. 1176-1184.

5. Beverly, E.A., et al., *Changing first-year medical students' attitudes toward primary care.* Fam Med, 2014. **46**(9): p. 707-12.

6. Bing-You, R.G., et al., *Using decentralized medical education to address the workforce needs of a rural state: a partnership between Maine Medical Center and Tufts University school of medicine.* Rural Remote Health, 2010. **10**(2): p. 1494.

7. Borgstrom, D.C. and S.J. Heneghan, *Bassett healthcare rural surgery experience.* Surg Clin North Am, 2009. **89**(6): p. 1321-3, viii-ix.

8. Breedlove, G., B. Lamping, and J.A. Smith, *The Kansas Health Education Training Center: caring for the underserved.* Kansas Nurse, 2006. **81**(3): p. 1-3.

9. Breon, T.A., *Rural surgical practice: an Iowa group model.* Surg Clin North Am, 2009. **89**(6): p. 1359-66, x.

10. Brooks, K.D., D.S. Eley, and T. Zink, *Profiles of rural longitudinal integrated clerkship students: a descriptive study of six consecutive student cohorts *.* Med Teach, 2014. **36**(2): p. 148-54.

11. Buerhaus, P.I., et al., *Practice characteristics of primary care nurse practitioners and physicians.* Nurs Outlook, 2015. **63**(2): p. 144-53.

12. Bushardt, R.L., F.K. Whitt, and T. Gregory, *Training physician assistants for rural Appalachia: an academic partnership for interprofessional collaboration.* N C Med J, 2014. **75**(1): p. 53-5.

13. Canon, S., et al., *A pilot study of telemedicine for post-operative urological care in children.* J Telemed Telecare, 2014. **20**(8): p. 427-30.

14. Cathcart-Rake, W., M. Robinson, and A. Paolo, *From Infancy to Adolescence: The Kansas University School of Medicine-Salina: A Rural Medical Campus Story.* Acad Med, 2016.

15. Cheng, D. and J. Fernandez, *Emergency medicine residency underrepresentation in rural states.* Am J Emerg Med, 2005. **23**(1): p. 92-3.

16. Chung-Do, J., et al., *Rural mental health: implications for telepsychiatry in clinical service, workforce development, and organizational capacity.* Telemed J E Health, 2012. **18**(3): p. 244-6.

17. Clark, D.B., et al., *Screening for Underage Drinking and Diagnostic and Statistical Manual of Mental Disorders, 5th Edition Alcohol Use Disorder in Rural Primary Care Practice.* Journal of Pediatrics, 2016. **173**: p. 214-220.

18. Cogbill, T.H. and B.T. Jarman, *Rural general surgery training: the Gundersen Lutheran approach.* Surg Clin North Am, 2009. **89**(6): p. 1309-12, viii.

19. Colegrove, D.J. and B.E. Whitacre, *Interest in rural medicine among osteopathic residents and medical students.* Rural Remote Health, 2009. **9**(3): p. 1192.

20. Colon-Gonzalez, M.C., et al., *"Someone's got to do it" - Primary care providers (PCPs) describe caring for rural women with mental health problems.* Ment Health Fam Med, 2013. **10**(4): p. 191-202.

21. Colwill, J.M., J.M. Cultice, and R.L. Kruse, *Will generalist physician supply meet demands of an increasing and aging population?* Health Aff (Millwood), 2008. **27**(3): p. w232-41.

22. Conger, M.M. and K.A. Plager, *Advanced nursing practice in rural areas: connectedness versus disconnectedness.* Online Journal of Rural Nursing & Health Care, 2008. **8**(1): p. 24-38.

23. Cook, A.F. and H. Hoas, *Ethics and rural healthcare: what really happens? What might help?* American Journal of Bioethics, 2008. **8**(4): p. 52-6.

24. Cook, M.R., et al., *A nonmetropolitan surgery clerkship increases interest in a surgical career.* American Journal of Surgery, 2015. **209**(1): p. 21-25.

25. Cosgrove, E.M., et al., *Addressing physician shortages in New Mexico through a combined BA/MD program.* Acad Med, 2007. **82**(12): p. 1152-7.

26. Cowley, D.S., et al., *Educating Psychiatry Residents to Practice in Smaller Communities: A Regional Residency Track Model.* Acad Psychiatry, 2016. **40**(5): p. 846-9.

27. Crump, W.J. and R.S. Fricker, *A medical school prematriculation program for rural students: Staying connected with place, cultivating a special connection with people.* Teaching and Learning in Medicine, 2015. **27**(4): p. 422-430.

28. Crump, W.J., R.S. Fricker, and A.M. Crump, *Just what are rural premedical students thinking? A report of the first 6 years of a pathways program.* J Rural Health, 2010. **26**(1): p. 97-9.

29. Decker, M.R., et al., *The General Surgery Job Market: Analysis of Current Demand for General Surgeons and Their Specialized Skills.* Journal of the American College of Surgeons, 2013. **217**(6): p. 1133-1139.

30. Dehn, R.W., *Workforce issues impacting PAs.* JAAPA: Journal of the American Academy of Physician Assistants, 2008. **21**(1): p. 56-7.

31. DeKastle, R., *Telesurgery: providing remote surgical observations for students.* AORN Journal, 2009. **90**(1): p. 93-101.

32. Dennison, D.A., et al., *Training health care professionals to manage overweight adolescents: experience in rural Georgia communities.* J Rural Health, 2008. **24**(1): p. 55-9.

33. Dent, M.M., et al., *Chronic disease management: teaching medical students to incorporate community.* Fam Med, 2010. **42**(10): p. 736-40.

34. Deutchman, M.E., et al., *Interdisciplinary rural immersion week.* Rural Remote Health, 2012. **12**: p. 2045.

35. Dick, J.F., 3rd, et al., *The effect of rural training experiences during residency on the selection of primary care careers: a retrospective cohort study from a single large internal medicine residency program.* Teach Learn Med, 2011. **23**(1): p. 53-7.

36. Donnon, T., W. Woloschuk, and D. Myhre, *Issues related to medical students' engagement in integrated rural placements: an exploratory factor analysis.* Can J Rural Med, 2009. **14**(3): p. 105-10.

37. Downey, L.H., et al., *Undergraduate rural medical education program development: focus group consultation with the NRHA Rural Medical Educators Group.* J Rural Health, 2011. **27**(2): p. 230-8.

38. Eidson-Ton, W.S., et al., *Training Medical Students for Rural, Underserved Areas: A Rural Medical Education Program in California.* Journal of Health Care for the Poor and Underserved, 2016. **27**(4): p. 1674-1688.

39. Elam, C.L., et al., *Discerning applicants' interests in rural medicine: a textual analysis of admission essays.* Med Educ Online, 2015. **20**(1): p. 27081.

40. Eley, D.S., et al., *Personality Profiles of Rural Longitudinal Integrated Clerkship Students Who Choose Family Medicine.* Family Medicine, 2015. **47**(3): p. 194-203.

41. Evans, D.V., et al., *Do Residencies That Aim to Produce Rural Family Physicians Offer Relevant Training?* Fam Med, 2016. **48**(8): p. 596-602.

42. Fannin, J.M. and J.N. Barnes, *Recruitment of physicians to rural America: a view through the lens of Transaction Cost Theory.* J Rural Health, 2007. **23**(2): p. 141-9.

43. Felix, H.C., E.B. Wootten, and M.K. Stewart, *The Arkansas Southern Rural Access Program: strategies for improving health care in rural areas of the state.* J Ark Med Soc, 2005. **101**(12): p. 366-8.

44. Fogarty, J.P., et al., *Florida State University College of Medicine: from ideas to outcomes.* Acad Med, 2012. **87**(12): p. 1699-704.

45. Foster, P.P., et al., *Recruitment of rural physicians in a diabetes internet intervention study: overcoming challenges and barriers.* J Natl Med Assoc, 2010. **102**(2): p. 101-7.

46. Freed, G.L., et al., *Family Nurse Practitioners: Roles and Scope of Practice in the Care of Pediatric Patients.* Pediatrics, 2010. **126**(5): p. 861-864.

47. Frellick, M., *The nurse practitioner will see you now. Advanced practice providers fill the physician gap.* Hosp Health Netw, 2011. **85**(7): p. 44-6, 48-9.

48. Frintner, M.P. and W.L. Cull, *Pediatric training and career intentions, 2003–2009.* Pediatrics, 2012. **129**(3): p. 522-528.

49. Frintner, M.P., et al., *Pediatric resident debt and career intentions.* Pediatrics, 2013. **131**(2): p. 312-318.

50. Gazewood, J.D., L.K. Rollins, and S.S. Galazka, *Beyond the horizon: the role of academic health centers in improving the health of rural communities.* Acad Med, 2006. **81**(9): p. 793-7.

51. Geske, J.A., et al., *Influence of a rural family medicine rotation on residency selection: MS3 versus MS4.* Fam Med, 2011. **43**(8): p. 556-9.

52. Giles, W.H., et al., *Education of the rural surgeon: experience from Tennessee.* Surg Clin North Am, 2009. **89**(6): p. 1313-9, viii.

53. Gillman, L.M. and A. Vergis, *General surgery graduates may be ill prepared to enter rural or community surgical practice.* Am J Surg, 2013. **205**(6): p. 752-7.

54. Greer, T., et al., *The WWAMI Targeted Rural Underserved Track (TRUST) Program: An Innovative Response to Rural Physician Workforce Shortages.* Acad Med, 2016. **91**(1): p. 65-9.

55. Groth, H., et al., *Board-certified emergency physicians comprise a minority of the emergency department workforce in iowa.* West J Emerg Med, 2013. **14**(2): p. 186-90.

56. Halaas, G.W., et al., *Clinical skills day: preparing third year medical students for their rural rotation.* Rural Remote Health, 2007. **7**(4): p. 788.

57. Heidelbaugh, J., J. Cooke, and L. Wimsatt, *Opportunities for medical student engagement with family medicine.* Fam Med, 2013. **45**(7): p. 484-91.

58. Hilty, D.M., et al., *A randomized, controlled trial of disease management modules, including telepsychiatric care, for depression in rural primary care.* Psychiatry (Edgmont), 2007. **4**(2): p. 58-65.

59. Hixon, A.L., L.E. Buenconsejo-Lum, and C.P. Racsa, *GIS residency footprinting: analyzing the impact of family medicine graduate medical education in Hawai'i.* Hawaii J Med Public Health, 2012. **71**(4 Suppl 1): p. 31-9.

60. Hughes, S., et al., *Trends in rural and urban deliveries and vaginal births: California 1998-2002.* J Rural Health, 2008. **24**(4): p. 416-22.

61. Kaplan, L., et al., *Rural-urban practice patterns of nurse practitioners in Washington State.* Journal for Nurse Practitioners, 2009. **5**(3): p. 169-75.

62. Keenum, A.J., et al., *Tennessee emergency medicine workforce, 2009.* Tenn Med, 2013. **106**(3): p. 41-3.

63. Kemper, L., et al., *2012 rural Medicare Advantage quality ratings and bonus payments.* Rural Policy Brief, 2014(2014 1): p. 1-4.

64. Kippenbrock, T., et al., *Minimal Changes and Missed Opportunities: A Decade Look at Nurse Practitioners in the Lower Mississippi River Delta States.* Journal of Professional Nursing, 2014. **30**(3): p. 266-272.

65. Kirby, B.A., *The rural rotation in a medical technology program: a ten-year retrospective study.* Clin Lab Sci, 2007. **20**(4): p. 202-9.

66. Kost, A., et al., *Medical Student Participation in Family Medicine Department Extracurricular Experiences and Choosing to Become a Family Physician.* Fam Med, 2015. **47**(10): p. 763-9.

67. Kosteniuk, J., et al., *Who steers the ship? Rural family physicians' views on collaborative care models for patients with dementia.* Prim Health Care Res Dev, 2014. **15**(1): p. 104-10.

68. Kozhimannil, K.B., et al., *The Rural Obstetric Workforce in US Hospitals: Challenges and Opportunities.* Journal of Rural Health, 2015. **31**(4): p. 365-372.

69. Laditka, J.N., S.B. Laditka, and J.C. Probst, *More may be better: evidence of a negative relationship between physician supply and hospitalization for ambulatory care sensitive conditions.* Health Serv Res, 2005. **40**(4): p. 1148-66.

70. LeBlanc, K.E., *Fostering rural physicians: the early success of the rural scholars track at Louisiana State University Health Sciences Center School of Medicine--New Orleans.* J La State Med Soc, 2008. **160**(3): p. 142, 144-6, 148.

71. Li, P., M.M. Ward, and J.E. Schneider, *Factors associated with Iowa rural hospitals' decision to convert to critical access hospital status.* J Rural Health, 2009. **25**(1): p. 70-6.

72. Lindeke, L., A. Jukkala, and M. Tanner, *Perceived barriers to nurse practitioner practice in rural settings.* J Rural Health, 2005. **21**(2): p. 178-81.

73. Lipkin, M., et al., *Two decades of Title VII support of a primary care residency: process and outcomes.* Acad Med, 2008. **83**(11): p. 1064-70.

74. Liu, J.J., et al., *Bypass of local primary care in rural counties: effect of patient and community characteristics.* Annals of Family Medicine, 2008. **6**(2): p. 124-30.

75. Longo, D.R., R.J. Gorman, and B. Ge, *Rural medical school applicants: do their academic credentials and admission decisions differ from those of nonrural applicants?* J Rural Health, 2005. **21**(4): p. 346-50.

76. Lorenzetti, R.C., et al., *The integration of clerkships: WVU's innovative approach to curriculum delivery at a regional campus.* W V Med J, 2011. **107**(6): p. 18-20, 22.

77. Macy, M.L., et al., *Looking Back on Rear-Facing Car Seats: Surveying US Parents in 2011 and 2013.* Acad Pediatr, 2014.

78. Mader, E.M., et al., *Clinical training in the rural setting: using photovoice to understand student experiences.* Rural Remote Health, 2016. **16**(2): p. 3877.

79. Makaroff, L.A., et al., *Factors Influencing Family Physicians' Contribution to the Child Health Care Workforce.* Annals of Family Medicine, 2014. **12**(5): p. 427-431.

80. Marth, N.J., *Advanced practice registered nurse (APRN) supply in a rural state : trends to inform policy*. 2010. p. vii, 74 leaves.

81. McCollister, H.M., et al., *Building and maintaining a successful surgery program in rural Minnesota.* Surg Clin North Am, 2009. **89**(6): p. 1349-57, ix.

82. Miller, B.F., et al., *Primary care, behavioral health, provider colocation, and rurality.* J Am Board Fam Med, 2014. **27**(3): p. 367-74.

83. Mistretta, M.J., *Differential effects of economic factors on specialist and family physician distribution in Illinois: a county-level analysis.* J Rural Health, 2007. **23**(3): p. 215-21.

84. Moesinger, R. and B. Hill, *Establishing a rural surgery training program: a large community hospital, expert subspecialty faculty, specific goals and objectives in each subspecialty, and an academic environment lay a foundation.* J Surg Educ, 2009. **66**(2): p. 106-12.

85. Mohr, N.M., et al., *Characterizing critical care physician staffing in rural America: a description of Iowa intensive care unit staffing.* J Crit Care, 2014. **29**(2): p. 194-8.

86. Mueller, K.J., et al., *Lessons From Tele-Emergency: Improving Care Quality And Health Outcomes By Expanding Support For Rural Care Systems.* Health Affairs, 2014. **33**(2): p. 228-34.

87. Murphy, K.L., et al., *Rural and nonrural differences in providing care for children with complex chronic conditions.* Clin Pediatr (Phila), 2012. **51**(5): p. 498-503.

88. Nation, C.L., A. Gerstenberger, and D. Bullard, *Preparing for change: the plan, the promise, and the parachute.* Acad Med, 2007. **82**(12): p. 1139-44.

89. Nelson, R.E., et al., *Cost effectiveness of training rural providers to perform joint injections.* Arthritis Care Res (Hoboken), 2014. **66**(4): p. 559-66.

90. Nuss, M.A., B. Robinson, and P.F. Buckley, *A Statewide Strategy for Expanding Graduate Medical Education by Establishing New Teaching Hospitals and Residency Programs.* Acad Med, 2015. **90**(9): p. 1264-8.

91. Opoku, S.T., et al., *A Comparison of the J-1 Visa Waiver and Loan Repayment Programs in the Recruitment and Retention of Physicians in Rural Nebraska.* J Rural Health, 2015. **31**(3): p. 300-9.

92. Overman, B.A., L. Petri, and U. Knoki-Wilson, *A view from inside Arizona and New Mexico Indian country: pursuing a health career path.* Rural Remote Health, 2007. **7**(2): p. 682.

93. Owen, J.A., et al., *Predicting rural practice using different definitions to classify medical school applicants as having a rural upbringing.* J Rural Health, 2007. **23**(2): p. 133-40.

94. Pathman, D.E., et al., *States' Experiences With Loan Repayment Programs for Health Care Professionals in a Time of State Budget Cuts and NHSC Expansion.* Journal of Rural Health, 2012. **28**(4): p. 408-415.

95. Pathman, D.E. and T.C. Ricketts, 3rd, *Interdependence of general surgeons and primary care physicians in rural communities.* Surg Clin North Am, 2009. **89**(6): p. 1293-302, vii-viii.

96. Petelin, J.B., M.E. Nelson, and J. Goodman, *Deployment and early experience with remote-presence patient care in a community hospital.* Surg Endosc, 2007. **21**(1): p. 53-6.

97. Phillips, R.L., Jr., et al., *Family physicians in the child health care workforce: opportunities for collaboration in improving the health of children.* Pediatrics, 2006. **118**(3): p. 1200-6.

98. Porter, J., et al., *How we incorporated service learning into a medical student rural clinical training experience.* Med Teach, 2016. **38**(4): p. 353-7.

99. Pretorius, R.W., D.A. Milling, and D. McGuigan, *Influence of a rural background on a medical student's decision to specialize in family medicine.* Rural Remote Health, 2008. **8**(3): p. 928.

100. Ray, T.E., *Perceptions of Nurse Practitioners on Rural and Remote Health Care Practice.* Walden University, 2014. **183**.

101. Rayman, K.M. and J. Edwards, *Rural primary care providers' perceptions of their role in the breast cancer care continuum.* J Rural Health, 2010. **26**(2): p. 189-95.

102. Reed, A.J., et al., *Assessment of Factors for Recruiting and Retaining Medical Students to Rural Communities Using the Community Apgar Questionnaire.* Fam Med, 2017. **49**(2): p. 132-136.

103. Reed, A.J., et al., *Association of "Grit" and Satisfaction in Rural and Nonrural Doctors.* Journal of the American Board of Family Medicine, 2012. **25**(6): p. 832-839.

104. Reschovsky, J.D. and A.B. Staiti, *Access and quality: does rural America lag behind?* Health Aff (Millwood), 2005. **24**(4): p. 1128-39.

105. Rhoads Kinder, S.J., A. Cherepski, and K. Freeman, *E-Learning in Obstetrics and HIV: Utilizing Online Interactive Scenarios to Replicate Critical Decision Making Moments.* JOGNN: Journal of Obstetric, Gynecologic & Neonatal Nursing, 2014. **43**(Supp 1): p. S5-6.

106. Richardson, I., et al., *A rural-urban comparison of allied health professionals' average hourly wage.* J Allied Health, 2010. **39**(3): p. e91-6.

107. Ricketts, T.C., *The migration of physicians and the local supply of practitioners: a five-year comparison.* Acad Med, 2013. **88**(12): p. 1913-8.

108. Ricketts, T.C. and R. Randolph, *Urban-rural flows of physicians.* Journal of Rural Health, 2007. **23**(4): p. 277-285.

109. Ricketts, T.C. and R. Randolph, *The diffusion of physicians.* Health Affairs, 2008. **27**(5): p. 1409-15.

110. Rittenhouse, D.R., et al., *Impact of Title VII training programs on community health center staffing and national health service corps participation.* Ann Fam Med, 2008. **6**(5): p. 397-405.

111. Roh, C.Y., K.H. Lee, and M.D. Fottler, *Determinants of hospital choice of rural hospital patients: the impact of networks, service scopes, and market competition.* J Med Syst, 2008. **32**(4): p. 343-53.

112. Roseamelia, C., et al., *A Qualitative Study of Medical Students in a Rural Track: Views on Eventual Rural Practice.* Family Medicine, 2014. **46**(4): p. 259-266.

113. Rosenblatt, R.A., et al., *Will rural family medicine residency training survive?* Fam Med, 2006. **38**(10): p. 706-11.

114. Rossi, A., et al., *Continuity of care in a rural critical access hospital: Surgeons as primary care providers.* American Journal of Surgery, 2011. **201**(3): p. 359-362.

115. Royston, P.J., et al., *Medical student characteristics predictive of intent for rural practice.* Rural and Remote Health, 2012. **12**(3).

116. Sawyer, B.T. and A.A. Ginde, *Scope of practice and autonomy of physician assistants in rural versus urban emergency departments.* Acad Emerg Med, 2014. **21**(5): p. 520-5.

117. Schwartz, M.R., *The physician pipeline to rural and underserved areas in Pennsylvania.* J Rural Health, 2008. **24**(4): p. 384-9.

118. Seidel, R.W. and M.D. Kilgus, *Agreement between telepsychiatry assessment and face-to-face assessment for Emergency Department psychiatry patients.* Journal of Telemedicine and Telecare, 2014. **20**(2): p. 59-62.

119. Shannon, C.K., *A gender-based study of attitudes and practice characteristics of rural physicians in West Virginia.* W V Med J, 2006. **102**(6): p. 22-5.

120. Shannon, C.K., et al., *Evaluation of a required statewide interdisciplinary Rural Health Education Program: student attitudes, career intents and perceived quality.* Rural Remote Health, 2005. **5**(4): p. 405.

121. Shapiro, J. and R. Longenecker, *Country doctors in literature: helping medical students understand what rural practice is all about.* Acad Med, 2005. **80**(8): p. 724-7.

122. Silverman, S.B. *Relationship between residency training and practice location in primary care residency programs in Texas*. [Internet Resource; Computer File] 2007; 1 online resource (xv, 219 leaves) : ill., maps Dissertation: Thesis (Ph. D.)--University of Texas at Austin, 2007.]. Available from: https://repositories.lib.utexas.edu/bitstream/handle/2152/3687/silvermans06643.pdf.

123. Sirinek, K.R., R. Willis, and R.M. Stewart, *Geographic maldistribution of general surgery PGYI residents: another US surgical desert.* American Journal of Surgery, 2014. **208**(6): p. 1023-1027.

124. Slagle, D.R., R.L. Byington, and E.L. Verhovsek, *Rural Versus Urban: Tennessee Health Administrators' Strategies on Recruitment and Retention for Allied Health Professionals.* Journal of Health Care Finance, 2012. **38**(4): p. 91-104.

125. Smoot, R.L. and D.R. Farley, *Minnesota general surgeons. Where do they come from?* Minn Med, 2006. **89**(12): p. 46-8.

126. Snyder, C.L., *Temporal geomapping of pediatric surgeons in the United States.* J Pediatr Surg, 2008. **43**(3): p. 424-9.

127. Stingley, S. and H. Schultz, *Helmsley Trust Support For Telehealth Improves Access To Care In Rural And Frontier Areas.* Health Affairs, 2014. **33**(2): p. 336-341.

128. Tatkon-coker, A.L., *The relationship of medicare and medicaid reimbursement on job dissatisfaction and spousal influence on the retention of rural health practitioners in North Central Iowa and South Central Minnesota*. 2007. p. xiv, 279 leaves ; 29 cm. Dissertation: Thesis (DBA) -- Nova Southeastern University, 2007.

129. Taylor, J.D. and S.E. Goletz, *Using area health education centers to promote interest in rural practice.* Rural Remote Health, 2016. **16**(3): p. 3934.

130. Taylor, J.D., et al., *Does an AHEC-Sponsored Clerkship Experience Strengthen Medical Students' Intent to Provide Care for Medically Underserved Patients?* J Community Health, 2015. **40**(6): p. 1173-7.

131. Toner, J.A., K.D. Ferguson, and R.D. Sokal, *Continuing interprofessional education in geriatrics and gerontology in medically underserved areas.* J Contin Educ Health Prof, 2009. **29**(3): p. 157-60.

132. Tong, S.T., et al., *Family physicians in the maternity care workforce: factors influencing declining trends.* Matern Child Health J, 2013. **17**(9): p. 1576-81.

133. Vanleit, B. and J. Cubra, *Student-developed problem-based learning cases: preparing for rural healthcare practice.* Rural Remote Health, 2005. **5**(4): p. 399.

134. Vickery, K.D., et al., *Preparing the Next Generation of Family Physicians to Improve Population Health: A CERA Study.* Fam Med, 2015. **47**(10): p. 782-8.

135. Waddimba, A.C., et al., *Validation of Single-Item Screening Measures for Provider Burnout in a Rural Health Care Network.* Eval Health Prof, 2016. **39**(2): p. 215-25.

136. Wadman, M.C., et al., *The impact of a rural emergency department rotation on applicant ranking of a US emergency medicine residency program.* Rural & Remote Health, 2007. **7**(4): p. 686.

137. Wayne, S.J., et al., *Early predictors of physicians' practice in medically underserved communities: a 12-year follow-up study of University of New Mexico School of Medicine graduates.* Acad Med, 2010. **85**(10 Suppl): p. S13-6.

138. Weeks, W.B. and A.E. Wallace, *Rural-urban differences in primary care physicians' practice patterns, characteristics, and incomes.* Journal of Rural Health, 2008. **24**(2): p. 161-170.

139. Wheat, J.R., et al., *The Rural Medical Scholars Program Study: Data to Inform Rural Health Policy.* Journal of the American Board of Family Medicine, 2011. **24**(1): p. 93-101.

140. Williams, D., et al., *Perspectives of Behavioral Health Clinicians in a Rural Integrated Primary Care/Mental Health Program.* Journal of Rural Health, 2015. **31**(4): p. 346-353.

141. Wimmer, P. *Professionalism among medical practitioners a case study of rural physicians*. [Internet Resource; Computer File] 2007; Available from: http://scholar.lib.vt.edu/theses/available/etd-02232007-083630

142. Wolf, L. and A.M. Delao, *Identifying the Educational Needs of Emergency Nurses in Rural and Critical Access Hospitals.* Journal of Continuing Education in Nursing, 2013. **44**(9): p. 424-8.

143. Wright, D.B., *Care in the country: a historical case study of long-term sustainability in 4 rural health centers.* American Journal of Public Health, 2009. **99**(9): p. 1612-8.

144. Wright, K.M., et al., *Finding the Perfect Match: Factors That Influence Family Medicine Residency Selection.* Fam Med, 2016. **48**(4): p. 279-85.

145. Xu, X., et al., *Malpractice burden, rural location, and discontinuation of obstetric care: a study of obstetric providers in Michigan.* J Rural Health, 2009. **25**(1): p. 33-42.

146. Zayas, L.E. and D. McGuigan, *Experiences promoting healthcare career interest among high-school students from underserved communities.* Journal of the National Medical Association, 2006. **98**(9): p. 1523-1531.

147. Zink, T., G.W. Halaas, and K.D. Brooks, *Learning professionalism during the third year of medical school in a 9-month-clinical rotation in rural Minnesota.* Med Teach, 2009. **31**(11): p. 1001-6.

148. Zink, T., et al., *The rural physician associate program: the value of immersion learning for third-year medical students.* J Rural Health, 2008. **24**(4): p. 353-9.

149. Zink, T., et al., *Is there equivalency between students in a longitudinal, rural clerkship and a traditional urban-based program?* Fam Med, 2010. **42**(10): p. 702-6.

### Timing – outcome data are too old (practicing in rural care before 2005)

1. Backer, E.L., et al., *The characteristics of successful family physicians in rural Nebraska: A qualitative study of physician interviews.* Journal of Rural Health, 2006. **22**(2): p. 189-191.

2. Baldwin, L., et al., *Modeling the mental health workforce in Washington State: using state licensing data to examine provider supply in rural and urban areas.* Journal of Rural Health, 2003. **22**(1): p. 50-8.

3. Brown, S.R. and B. Birnbaum, *Student and resident education and rural practice in the Southwest Indian Health Service: a physician survey.* Fam Med, 2005. **37**(10): p. 701-5.

4. Daniels, Z.M., et al., *Factors in recruiting and retaining health professionals for rural practice.* J Rural Health, 2007. **23**(1): p. 62-71.

5. Doty, B., et al., *Is a broadly based surgical residency program more likely to place graduates in rural practice?* World Journal of Surgery, 2006. **30**(12): p. 2089-2093.

6. Edwards, J.B., et al., *Education for Rural Practice--Practice Locations of Graduates of Family Physician Residency and Nurse Practitioner Programs: Considerations within the Context of Institutional Culture and Curricular Innovation through Titles VII and VIII.* Journal of Rural Health, 2006. **22**(1): p. 69-77.

7. Evans, T.C., et al., *Academic degrees and clinical practice characteristics: the University of Washington physician assistant program: 1969-2000.* J Rural Health, 2006. **22**(3): p. 212-9.

8. Florence, J.A., et al., *Rural health professions education at East Tennessee State University: survey of graduates from the first decade of the community partnership program.* J Rural Health, 2007. **23**(1): p. 77-83.

9. Hughes, S., et al., *High school census tract information predicts practice in rural and minority communities.* J Rural Health, 2005. **21**(3): p. 228-32.

10. Krist, A.H., et al., *Title VII funding and physician practice in rural or low-income areas.* J Rural Health, 2005. **21**(1): p. 3-11.

11. Lang, F., et al., *The Appalachian Preceptorship: over two decades of an integrated clinical-classroom experience of rural medicine and Appalachian culture.* Acad Med, 2005. **80**(8): p. 717-23.

12. Maudlin, R.K. and G.R. Newkirk, *Family Medicine Spokane Rural Training Track: 24 years of rural-based graduate medical education.* Fam Med, 2010. **42**(10): p. 723-8.

13. Miller, T., R.S. Hooker, and D.A. Mains, *Characteristics of osteopathic physicians choosing to practice rural primary care.* J Am Osteopath Assoc, 2006. **106**(5): p. 274-9.

14. Morris, A.L., et al., *International medical graduates in family medicine in the United States of America: an exploration of professional characteristics and attitudes.* Hum Resour Health, 2006. **4**: p. 17.

15. Pacheco, M., et al., *The impact on rural New Mexico of a family medicine residency.* Acad Med, 2005. **80**(8): p. 739-44.

16. Peterson, L.E., et al., *Nonemergency medicine-trained physician coverage in rural emergency departments.* J Rural Health, 2008. **24**(2): p. 183-8.

17. Ruff, C.C., et al., *Development and outcomes of a rural track within a primary care physician assistant program.* Journal of Physician Assistant Education, 2006. **17**(4): p. 37-41.

18. Smucny, J., et al., *An evaluation of the Rural Medical Education Program of the State University Of New York Upstate Medical University, 1990-2003.* Acad Med, 2005. **80**(8): p. 733-8.

19. Thomas, C.R. and C.E. Holzer, 3rd, *The continuing shortage of child and adolescent psychiatrists.* J Am Acad Child Adolesc Psychiatry, 2006. **45**(9): p. 1023-31.

20. Wheat, J.R., et al., *Rural health leaders pipeline, 1990-2005: case study of a second-generation rural medical education program.* J Agromedicine, 2007. **12**(4): p. 51-61.

### Not US setting

1. *Rural Retention Program (RRP) policy framework for health authorities*. [Internet Resource; Computer File] 2012; 1 online resource (17 p.)]. Available from: http://www.llbc.leg.bc.ca/public/pubdocs/bcdocs2013/526533/retentionprogram.pdf.

2. Bardella, I.J., *Solutions for recruitment and retention of rural and remote health workforce.* Fam Med, 2010. **42**(4): p. 288-9.

3. Bragard, I., et al., *Quality of work life of rural emergency department nurses and physicians: a pilot study.* BMC Res Notes, 2015. **8**: p. 116.

4. Cayley, W., Jr., *Patients as allies in student education and rural recruitment.* Fam Med, 2010. **42**(4): p. 288.

5. Chen, L.C., *Striking the right balance: health workforce retention in remote and rural areas.* Bull World Health Organ, 2010. **88**(5): p. 323, a.

6. Couper, I.D., et al., *Influences on the choice of health professionals to practice in rural areas.* S Afr Med J, 2007. **97**(11): p. 1082-6.

7. Deirdre, J., M. Florence, and Y. Olive. *Putting the (R) Ural in Preceptorship*. [Internet Resource; Archival Material] 2012; Available from: http://dx.doi.org/10.1155/2012/528580

8. Eley, D., L. Young, and T.R. Przybeck, *Exploring temperament and character traits in medical students; a new approach to increase the rural workforce.* Med Teach, 2009. **31**(3): p. e79-84.

9. Eley, D., L. Young, and T.R. Przybeck, *Exploring the Temperament and Character Traits of Rural and Urban Doctors.* Journal of Rural Health, 2009. **25**(1): p. 43-49.

10. Haji, M., et al., *Emerging opportunities for recruiting and retaining a rural health workforce through decentralized health financing systems.* Bull World Health Organ, 2010. **88**(5): p. 397-9.

11. Igumbor, E.U. and E.N. Kwizera, *The positive impact of rural medical schools on rural intern choices.* Rural Remote Health, 2005. **5**(2): p. 417.

12. Kuhn, M.K.M. and C. Ochsen. *Demographic and geographic determinants of regional physician supply*. [Internet Resource; Archival Material] 2009; Available from: http://hdl.handle.net/10419/39775

13. Lee, J., et al., *The role of distributed education in recruitment and retention of family physicians.* Postgraduate Medical Journal, 2016. **92**(1090): p. 436-440.

14. Peach, H.G., *Rural placement programs.* Rural Remote Health, 2011. **11**(3): p. 1844.

15. Peake, S.L. and N. Judd, *Supporting rural community-based critical care.* Current Opinion in Critical Care, 2007. **13**(6): p. 720-724.

16. Roberts, C., et al., *A longitudinal integrated placement and medical students' intentions to practise rurally.* Med Educ, 2012. **46**(2): p. 179-91.

17. Schneider, H.B., *Attracting medical students to rural areas.* Cmaj, 2008. **179**(8): p. 801.

18. Sen Gupta, T.K., et al., *Are medical student results affected by allocation to different sites in a dispersed rural medical school?* Rural Remote Health, 2011. **11**(1): p. 1511.

19. Singer, J., *Urban and rural health care environments: factors affecting new graduates' choice for employment.* Journal of Medical Imaging & Radiation Sciences, 2008. **39**(3): p. 173-4.

20. Solowiej, K., et al., *A scheme to support the recruitment and retention of allied health professionals to hard to fill posts in rural areas including commentaries by Kevin O'Toole; Matthew J Leach; Leonie Segal; Ana Manzano-Santaella and Crispin Coombs.* International Journal of Therapy & Rehabilitation, 2010. **17**(10): p. 545-55.

21. Stone, N., *The Rural Interprofessional Education Project (RIPE).* J Interprof Care, 2006. **20**(1): p. 79-81.

22. Strasser, R. and A.J. Neusy, *Context counts: training health workers in and for rural and remote areas.* Bull World Health Organ, 2010. **88**(10): p. 777-82.

23. Tolhurst, H.M., J. Adams, and S.M. Stewart, *An exploration of when urban background medical students become interested in rural practice.* Rural Remote Health, 2006. **6**(1): p. 452.

24. Vogel, L., *Rural premedicine program aims to tackle doctor shortages.* Cmaj, 2014. **186**(3): p. 177.

25. Whalley, D.S.B. *Why do practitioners work in deprived areas? : identifying affinity factors for urban deprived general practice*. [Internet Resource; Computer File] 2012; 1 online resource. Dissertation: Thesis (Ph.D.)--University of Manchester, 2012.]. Available from: http://www.manchester.ac.uk/escholar/uk-ac-man-scw:160829.

26. Whitelaw, A.S., et al., *Establishing a rural Emergency Medical Retrieval Service.* Emerg Med J, 2006. **23**(1): p. 76-8.

27. Woloschuk, W., et al., *Comparing the performance in family medicine residencies of graduates from longitudinal integrated clerkships and rotation-based clerkships.* Acad Med, 2014. **89**(2): p. 296-300.

28. Yonge, O., F. Myrick, and L. Ferguson, *The Challenge of Evaluation in Rural Preceptorship.* Online Journal of Rural Nursing & Health Care, 2011. **11**(2): p. 3-15.

29. Ypinazar, V.A. and S.A. Margolis, *Clinical simulators: applications and implications for rural medical education.* Rural Remote Health, 2006. **6**(2): p. 527.

### Retained as background, reviews, and more information on included studies (multiple publications)

1. *Challenges, solutions & opportunities : affordable housing, workforce training, recruitment & retention of health care professionals*. 2007, Northern Arizona University, the W.A. Franke College of Business.

2. *Shortage of general surgeons coming?* OR Manager, 2008. **24**(6).

3. *Young physicians not keen on rural areas.* Manag Care, 2012. **21**(10): p. 16.

4. Aseltine, R.H., Jr., M.C. Katz, and A.H. Geragosian, *Connecticut 2009 Primary Care Survey: physician satisfaction, physician supply and patient access to medical care.* Conn Med, 2010. **74**(5): p. 281-91.

5. Avery, D.M., Jr., et al., *Admission factors predicting family medicine specialty choice: a literature review and exploratory study among students in the Rural Medical Scholars Program.* J Rural Health, 2012. **28**(2): p. 128-36.

6. Baker, E., et al., *Rural Idaho Family Physicians' Scope of Practice.* Journal of Rural Health, 2010. **26**(1): p. 85-89.

7. Ballance, D., D. Kornegay, and P. Evans, *Factors that influence physicians to practice in rural locations: a review and commentary.* J Rural Health, 2009. **25**(3): p. 276-81.

8. Balshem, H., et al., *GRADE guidelines: 3. Rating the quality of evidence.* J Clin Epidemiol, 2011. **64**(4): p. 401-6.

9. Barnighausen, T. and D.E. Bloom, *Financial incentives for return of service in underserved areas: a systematic review.* BMC Health Serv Res, 2009. **9**: p. 86.

10. Barrett, F.A., M.S. Lipsky, and M.N. Lutfiyya, *The impact of rural training experiences on medical students: a critical review.* Acad Med, 2011. **86**(2): p. 259-63.

11. Bowman, R.C., *Measuring primary care: the standard primary care year.* Rural and Remote Health, 2008. **8**(3).

12. Bridgham, R.G., *Final report : HB 1615, (Chapter 367:3, Laws of 2008), establishing a Commission to Recommend Policies and Programs to Increase the Number of New Hampshire Individuals in Health Professions Servicing New Hampshire's Rural and Underserved Areas with a Focus on Primary Care*. 2009, Concord, N.H.: N.H. General Court. [6] p.

13. Broughan, T.A., *SAGES 2007 rural surgery panel.* Surg Endosc, 2008. **22**(7): p. 1579-81.

14. Carlton, E.L. and L.A. Simmons, *Health decision-making among rural women: physician access and prescription adherence.* Rural and Remote Health, 2011. **11**(1).

15. Chen, C., et al., *The redistribution of graduate medical education positions in 2005 failed to boost primary care or rural training.* Health Aff (Millwood), 2013. **32**(1): p. 102-10.

16. Chipp, C., et al., *"If only someone had told me...": lessons from rural providers.* J Rural Health, 2011. **27**(1): p. 122-30.

17. Cogbill, T.H., J.B. Cofer, and B.T. Jarman, *Contemporary issues in rural surgery.* Curr Probl Surg, 2012. **49**(5): p. 263-318.

18. Collier, D., *Rural Michigan physician retention study reveals motivators.* Mich Med, 2010. **109**(5): p. 21.

19. Cook, A.F. and H. Hoas, *Hide and seek: The elusive rural psychiatrist.* Academic Psychiatry, 2007. **31**(6): p. 419-422.

20. Corbett, C.D., *Recruitment and retention of physicians in rural North Dakota : testing for congruency between current policies and physician motivation*. 2012. p. ii, 37 leaves ; 29 cm. Dissertation: Paper (M.S.)--Minnesota State University Moorhead, 2012.

21. Crouse, B.J. and R.L. Munson, *The effect of the physician J-1 visa waiver on rural Wisconsin.* Wmj, 2006. **105**(7): p. 16-20.

22. Crump, W.J., et al., *Rural track training based at a small regional campus: equivalency of training, residency choice, and practice location of graduates.* Acad Med, 2013. **88**(8): p. 1122-8.

23. Curran, V., L. Rourke, and P. Snow, *A framework for enhancing continuing medical education for rural physicians: A summary of the literature.* Med Teach, 2010. **32**(11): p. e501-8.

24. Deveney, K., et al., *Association between dedicated rural training year and the likelihood of becoming a general surgeon in a small town.* JAMA Surg, 2013. **148**(9): p. 817-21.

25. Dill, M.J. and E.S. Salsberg, *The Complexities of Physician Supply and Demand: Projections Through 2025. November 2008*. 2008, Association of American Medical Colleges, Center for Workforce Studies.: Washington, DC.

26. Doescher, M.P., et al., *The contribution of physicians, physician assistants, and nurse practitioners toward rural primary care: findings from a 13-state survey.* Med Care, 2014. **52**(6): p. 549-56.

27. Dolea, C., L. Stormont, and J.M. Braichet, *Evaluated strategies to increase attraction and retention of health workers in remote and rural areas.* Bull World Health Organ, 2010. **88**(5): p. 379-85.

28. Dornan, T., et al., *How can experience in clinical and community settings contribute to early medical education? A BEME systematic review.* Medical Teacher, 2006. **28**(1): p. 3-18.

29. Dorsey, E.R., S. Nicholson, and W.H. Frist, *Commentary: improving the supply and distribution of primary care physicians.* Acad Med, 2011. **86**(5): p. 541-3.

30. Doty, B., et al., *Use of Locum Tenens Surgeons to Provide Surgical Care in Small Rural Hospitals.* World Journal of Surgery, 2009. **33**(2): p. 228-232.

31. Doty, B., et al., *General surgery at rural hospitals: a national survey of rural hospital administrators.* Surgery, 2008. **143**(5): p. 599-606.

32. Edwards, J.B., et al., *Practice locations of graduates of family physician residency and nurse practitioner programs: considerations within the context of institutional culture and curricular innovation through Titles VII and VIII.* J Rural Health, 2006. **22**(1): p. 69-77.

33. Escarce, J.J. and K. Kapur, *Do patients bypass rural hospitals? Determinants of inpatient hospital choice in rural California.* Journal of Health Care for the Poor and Underserved, 2009. **20**(3): p. 625-644.

34. Everitt-Deering, P. *The adoption of information and communication technologies by rural general practitioners a socio technical analysis*. [Internet Resource; Computer File; Archival Material] 2008; Available from: http://eprints.vu.edu.au/1412.

35. Farmer, J., et al., *A scoping review of the association between rural medical education and rural practice location.* Hum Resour Health, 2015. **13**: p. 27.

36. Filipova, A.A., *Factors influencing the satisfaction of rural physician assistants: a cross-sectional study.* J Allied Health, 2014. **43**(1): p. 22-31.

37. Fordyce, M.A., et al., *2005 physician supply and distribution in rural areas of the United States [Internet]*. 2007, Rural Health Research and Policy Centers: Seattle, WA.

38. Fournier, G.M. and C. Henderson, *Incentives and physician specialty choice: a case study of Florida's Program in Medical Sciences.* Inquiry, 2005. **42**(2): p. 160-70.

39. Fraher, E.P., et al., *Projecting surgeon supply using a dynamic model.* Ann Surg, 2013. **257**(5): p. 867-72.

40. Gagnon, M.P., et al., *Supporting health professionals through information and communication technologies: a systematic review of the effects of information and communication technologies on recruitment and retention.* Telemed J E Health, 2011. **17**(4): p. 269-74.

41. Garrison-Jakel, J., *Patching the rural workforce pipeline--why don't we do more?* J Rural Health, 2011. **27**(2): p. 239-40.

42. Getson, D.S., *Rural practice realities.* W V Med J, 2013. **109**(4): p. 34-7.

43. Glasser, M., et al., *A comprehensive medical education program response to rural primary care needs.* Acad Med, 2008. **83**(10): p. 952-61.

44. Goodfellow, A., et al., *Predictors of Primary Care Physician Practice Location in Underserved Urban or Rural Areas in the United States: A Systematic Literature Review.* Acad Med, 2016. **91**(9): p. 1313-21.

45. Grobler, L., et al., *Interventions for increasing the proportion of health professionals practising in rural and other underserved areas.* Cochrane Database Syst Rev, 2009(1): p. Cd005314.

46. Haggerty, T.S., et al., *Physician Wellness in Rural America: A Review.* International Journal of Psychiatry in Medicine, 2013. **46**(3): p. 303-13.

47. Halaas, G.W., *The Rural Physician Associate Program: successful outcomes in primary care and rural practice.* Rural Remote Health, 2005. **5**(2): p. 453.

48. Halaas, G.W., et al., *Recruitment and retention of rural physicians: outcomes from the rural physician associate program of Minnesota.* J Rural Health, 2008. **24**(4): p. 345-52.

49. Henry, L.R., R.S. Hooker, and K.L. Yates, *The role of physician assistants in rural health care: a systematic review of the literature.* J Rural Health, 2011. **27**(2): p. 220-9.

50. Hilty, D.M., et al., *Models of telepsychiatric consultation-liaison service to rural primary care.* Psychosomatics, 2006. **47**(2): p. 152-157.

51. Hirsh, D., L. Walters, and A.N. Poncelet, *Better learning, better doctors, better delivery system: possibilities from a case study of longitudinal integrated clerkships.* Med Teach, 2012. **34**(7): p. 548-54.

52. Holmes, G.M., *Increasing physician supply in medically underserved areas.* Labour Economics, 2005. **12**(5): p. 697-725.

53. Huff, C., *Where are the specialists?* Hosp Health Netw, 2011. **85**(12): p. 26-8, 31, 1.

54. Huff, C., *Please doc, stay. Give docs a reason to set up shop.* Hosp Health Netw, 2012. **86**(2): p. 20.

55. Huff, C., *Done recruiting? Start retaining.* Trustee, 2014. **67**(1): p. 8-12, 1.

56. Kochar, M.S., *The J-1 visa waiver program for rural Wisconsin.* Wmj, 2006. **105**(7): p. 13.

57. Larson, E.H. and L.G. Hart, *Growth and change in the physician assistant workforce in the United States, 1967-2000.* J Allied Health, 2007. **36**(3): p. 121-30.

58. Lee, D.M. and T. Nichols, *Physician recruitment and retention in rural and underserved areas.* Int J Health Care Qual Assur, 2014. **27**(7): p. 642-52.

59. Lindsay, S., *Gender differences in rural and urban practice location among mid-level health care providers.* J Rural Health, 2007. **23**(1): p. 72-6.

60. Lynch, S., *Hospice and palliative care access issues in rural areas.* Am J Hosp Palliat Care, 2013. **30**(2): p. 172-7.

61. Lynge, D.C., *Rural general surgeons: manpower and demographics.* Surg Endosc, 2008. **22**(7): p. 1593-4.

62. Lynge, D.C., et al., *A longitudinal analysis of the general surgery workforce in the United States, 1981-2005.* Archives of Surgery, 2008. **143**(4): p. 345-350.

63. MacDowell, M., et al., *Perspectives on rural health workforce issues: Illinois-Arkansas comparison.* J Rural Health, 2009. **25**(2): p. 135-40.

64. MacDowell, M., et al., *A national view of rural health workforce issues in the USA.* Rural Remote Health, 2010. **10**(3): p. 1531.

65. MacDowell, M., M. Glasser, and M. Hunsaker, *A decade of rural physician workforce outcomes for the Rockford Rural Medical Education (RMED) Program, University of Illinois.* Acad Med, 2013. **88**(12): p. 1941-7.

66. Maley, M., P. Worley, and J. Dent, *Using rural and remote settings in the undergraduate medical curriculum: AMEE Guide No. 47.* Medical Teacher, 2009. **31**(11): p. 969-983.

67. Mareck, D.G., *Federal and state initiatives to recruit physicians to rural areas.* Virtual Mentor, 2011. **13**(5): p. 304-9.

68. Mbemba, G., et al., *Interventions for supporting nurse retention in rural and remote areas: an umbrella review.* Hum Resour Health, 2013. **11**: p. 44.

69. Meyer, D., *Technology, job satisfaction, and retention: rural mental health practitioners.* J Rural Health, 2006. **22**(2): p. 158-63.

70. Mullan, F., S. Frehywot, and L.J. Jolley, *Aging, primary care, and self-sufficiency: health care workforce challenges ahead.* J Law Med Ethics, 2008. **36**(4): p. 703-8, 608.

71. Murphy, K.L., *Alaska health care workforce shortages : impact of state legislation*. 2011. p. ix, 74 leaves ; 28 cm. Dissertation: Thesis (M.Public Health)--University of Alaska Anchorage, 2011.

72. Myhre, D.L., S. Bajaj, and W. Jackson, *Determinants of an urban origin student choosing rural practice: a scoping review.* Rural Remote Health, 2015. **15**(3): p. 3483.

73. Nakayama, D.K. and T.G. Hughes, *Issues That Face Rural Surgery in the United States.* Journal of the American College of Surgeons, 2014. **219**(4): p. 814-818.

74. Nance, M.L., B.G. Carr, and C.C. Branas, *Access to pediatric trauma care in the United States.* Arch Pediatr Adolesc Med, 2009. **163**(6): p. 512-8.

75. Ortiz, J., et al., *Accountable care organizations: benefits and barriers as perceived by Rural Health Clinic management.* Rural Remote Health, 2013. **13**(2): p. 2417.

76. Palmer, R.T., *Exploring online community among rural medical education students: A case study.* Dissertation Abstracts International Section A: Humanities and Social Sciences, 2014. **75**(1-A(E)).

77. Pathman, D.E., *What outcomes should we expect from programs that pay physicians'training expenses in exchange for service?* N C Med J, 2006. **67**(1): p. 77-82.

78. Pathman, D.E., et al., *National Health Service Corps staffing and the growth of the local rural non-NHSC primary care physician workforce.* J Rural Health, 2006. **22**(4): p. 285-93.

79. Patterson, D.G., et al., *Recruitment of Non-U.S. Citizen Physicians to Rural and Underserved Areas through Conrad State 30 J-1 Visa Waiver Programs. Final Report #148, April 2015.* 2015, WWAMI Rural Health Research Center, University of Washington: Seattle, WA.

80. Patterson, D.G., et al. *January 2011 Policy Brief: Training Physicians for Rural Practice: Capitalizing on Local Expertise to Strengthen Rural Primary Care*. 2011 8/20/2015]; Available from: https://www.raconline.org/rtt/pdf/policybrief_jan11.pdf.

81. Patterson, D.G., et al. *January 2012 Policy Brief: Rural Residency Training for Family Medicine Physicians: Graduate Early-Career Outcomes*. 2012 8/20/2015]; Available from: https://www.raconline.org/rtt/pdf/rural-family-medicine-training-early-career-outcomes-2012.pdf.

82. Peterson, L.E., et al., *Rural-urban distribution of the U.S. Geriatrics physician workforce.* J Am Geriatr Soc, 2011. **59**(4): p. 699-703.

83. Pfarrwaller, E., et al., *Impact of Interventions to Increase the Proportion of Medical Students Choosing a Primary Care Career: A Systematic Review.* J Gen Intern Med, 2015. **30**(9): p. 1349-58.

84. Philipp, D.L. and D.L. Wright, *Recruiting healthcare professionals to rural areas.* Radiol Manage, 2005. **27**(6): p. 44-50.

85. Price, J., *The National Health Service Corps--a critical component of provider recruitment in North Carolina's rural and underserved communities.* N C Med J, 2010. **71**(3): p. 251.

86. Quarry, W.A., *A research study outlining the key issues and strategies needed to improve recruitment and retention among primary care physicians in rural communities*. 2012. iv, [55 leaves].

87. Rabinowitz, H.K., *AM last page. Truths about the rural physician supply.* Acad Med, 2011. **86**(2): p. 272.

88. Rabinowitz, H.K., et al., *Long-term retention of graduates from a program to increase the supply of rural family physicians.* Acad Med, 2005. **80**(8): p. 728-32.

89. Rabinowitz, H.K., et al., *Increasing the supply of rural family physicians: recent outcomes from Jefferson Medical College's Physician Shortage Area Program (PSAP).* Acad Med, 2011. **86**(2): p. 264-9.

90. Rabinowitz, H.K., et al., *Increasing the supply of women physicians in rural areas: outcomes of a medical school rural program.* J Am Board Fam Med, 2011. **24**(6): p. 740-4.

91. Rabinowitz, H.K., et al., *The relationship between matriculating medical students' planned specialties and eventual rural practice outcomes.* Acad Med, 2012. **87**(8): p. 1086-90.

92. Rabinowitz, H.K., et al., *Retention of rural family physicians after 20-25 years: outcomes of a comprehensive medical school rural program.* J Am Board Fam Med, 2013. **26**(1): p. 24-7.

93. Rabinowitz, H.K., et al., *Medical school programs to increase the rural physician supply: a systematic review and projected impact of widespread replication.* Acad Med, 2008. **83**(3): p. 235-43.

94. Rabinowitz, H.K., et al., *Medical school rural programs: a comparison with international medical graduates in addressing state-level rural family physician and primary care supply.* Acad Med, 2012. **87**(4): p. 488-92.

95. Ricketts, T.C., *Workforce issues in rural areas: A focus on policy equity.* American Journal of Public Health, 2005. **95**(1): p. 42-48.

96. Roh, C.Y. and M.J. Moon, *Nearby, but not wanted? The bypassing of rural hospitals and policy implications for rural health care systems.* Policy Studies Journal, 2005. **33**(3): p. 377-394.

97. Rosenblatt, R.A., et al., *Shortages of medical personnel at community health centers: implications for planned expansion.* Jama, 2006. **295**(9): p. 1042-9.

98. Scarbrough, A.W., et al., *Improving Primary Care Retention in Medically Underserved Areas What's a Clinic to Do?* Health Care Manager, 2016. **35**(4): p. 368-372.

99. Schmitz, D.F., et al., *Idaho rural family physician workforce study: the Community Apgar Questionnaire.* Rural Remote Health, 2011. **11**(3): p. 1769.

100. Seligson, R.W. and P.P. Highsmith, *North Carolina Medical Society Foundation's Community Practitioner Program.* N C Med J, 2006. **67**(1): p. 83-5.

101. Staton, F.S., et al., *How PAs improve access to care for the underserved.* Jaapa, 2007. **20**(6): p. 32, 34, 36 passim.

102. Stempniak, M., *The hiring headache. Rural hospitals band together to lure physicians.* Hosp Health Netw, 2012. **86**(12): p. 18-9.

103. Thompson, M.J., et al., *Do international medical graduates (IMGs) "fill the gap" in rural primary care in the United States? A national study.* J Rural Health, 2009. **25**(2): p. 124-34.

104. Thompson, M.J., et al., *Characterizing the general surgery workforce in rural America.* Arch Surg, 2005. **140**(1): p. 74-9.

105. Toner, J.A., K.D. Ferguson, and R.D. Sokal, *Continuing interprofessional education in geriatrics and gerontology in medically underserved areas ��.* Journal of Continuing Education in the Health Professions, 2009. **29**(3): p. 157-60.

106. Traverso, G. and G.T. McMahon, *Residency training and international medical graduates: Coming to America no more.* JAMA: Journal of the American Medical Association, 2012. **308**(21): p. 2193-2194.

107. Tumosa, N., et al., *Health care workforce development in rural america: when geriatrics expertise is 100 miles away.* Gerontol Geriatr Educ, 2012. **33**(2): p. 133-51.

108. Van Vleet, A. and J. Paradise, *Issue Brief: Tapping Nurse Practitioners to Meet Rising Demand for Primary Care*. 2015, The Henry J. Kaiser Family Foundation.

109. Vogt, H.B., *South Dakota's rural physician shortage: how might it be addressed?* S D Med, 2008. **61**(4): p. 125, 127.

110. Wade, M.E., et al., *Influence of hometown on family physicians' choice to practice in rural settings.* Fam Med, 2007. **39**(4): p. 248-54.

111. Walters, L., et al., *Outcomes of longitudinal integrated clinical placements for students, clinicians and society.* Medical Education, 2012. **46**(11): p. 1028-1041.

112. Weeks, W.B., et al., *Research on rural veterans: An analysis of the literature.* Journal of Rural Health, 2008. **24**(4): p. 337-344.

113. Weigel, P.A., et al., *Variation in Primary Care Service Patterns by Rural-Urban Location.* J Rural Health, 2016. **32**(2): p. 196-203.

114. Weldon, T., *Physician shortages and the medically underserved*, in *Trends in America; Variation: Trends in America.* 2008, Council of State Governments.

115. Wheat, J.R., et al., *Physicians for rural America: the role of institutional commitment within academic medical centers.* J Rural Health, 2005. **21**(3): p. 221-7.

116. Whitcomb, M.E., *The challenge of providing doctors for rural America.* Acad Med, 2005. **80**(8): p. 715-6.

117. Wilson, N.W., et al., *A critical review of interventions to redress the inequitable distribution of healthcare professionals to rural and remote areas.* Rural Remote Health, 2009. **9**(2): p. 1060.

118. Xierali, I.M., et al., *Increasing graduate medical education (GME) in critical access hospitals (CAH) could enhance physician recruitment and retention in rural America.* J Am Board Fam Med, 2012. **25**(1): p. 7-8.

119. Ziegler, C., *The association of medical student debt on choice of primary care specialty and rural practice location*. 2015.

120. Zuckerman, R., et al., *General surgery programs in small rural New York state hospitals: A pilot survey of hospital administrators.* Journal of Rural Health, 2006. **22**(4): p. 339-342.

121. Zurn, P., et al., *A technical framework for costing health workforce retention schemes in remote and rural areas.* Hum Resour Health, 2011. **9**: p. 8.

## Evidence Tables

### Geographic Choice Studies

| **ID** | **Participants** | **N, study design, outcome** | **Results – demographic background** | **Results – training** | **Results – financial aspects** | **Results – rural environment** | **Other results** | **Authors’ conclusions** |
| --- | --- | --- | --- | --- | --- | --- | --- | --- |
| Baker, 2012([1](#_ENREF_1)) | US medical schools  Physicians | N=44,894  2009 AMA Masterfile | N/A | 10 schools produced 51% of all US graduates who practiced primary care in rural Appalachian counties: the West Virginia School of Osteopathic Medicine ranked 1st, followed by the University of Pikeville Kentucky College of Osteopathic Medicine and the University of Alabama School of Medicine; 6 are located in Appalachian counties | N/A | N/A | N/A | Physicians practicing in Appalachia are largely graduates of schools in or near the region. |
| Chen, 2010([2](#_ENREF_2)) | Clinically active MDs, DOs, and international medical graduates (IMGs) who graduated from medical school between 1987-1997  Practicing physicians | N=175,649  Analytic study  Rural: RUCA and county designations  Outcome: Practicing in rural location in 2005 | In 2005, 18% of DOs, 11% of MDs, and 13% of IMGs are practicing in a rural location; 31% of rural physicians were women (37% of MDs and 31% of DOs); overall, 94% of physicians were MDs and 6% DOs, but 18% of DOs and 11% of MDs practice in rural care | Of the 1.4% MDs trained in rural residency, 36% were in rural practice; of the 3.6% DOs, 50% were in rural practice. Rural residents were 3x more likely to practice in rural areas (RR 3.4, p<.001). Rural residents account for 5% of MDs and 10% of DOs in rural areas. 60% of rural family medicine residents were 3x more likely to practice in rural care (RR 2.8, p<.001). Only 9% of rural family providers trained in a rural residency | N/A | N/A | N/A | The proportion and number of physicians entering rural practice has remained stable compared with earlier analyses. However, recent trends such as declining primary care interest are not yet reflected in these data and may portend worsening shortages of rural physicians. |
| Crump, 2016([3](#_ENREF_3)) ([4](#_ENREF_4)) | Louiseville medical students graduating 2001-2008  Medical students | N=1,120  Analytic study  Rural: RUCC  Outcome: Location of practice | Rural practice location was significantliiy associated with having a rural upbringing (OR 2.67) in a multivariate analysis; gender, age, and race were not associated | Choosinga rural practice location was significantly higher for graduates participating in the rural campus (OR 5.46) and for those choosing a family medicine residency (OR 5.08); the type of degree was not associated | No association for any level of dept with rural practice cites | N/A | Adding rural training to rural upbringing and specialty choice improved the model) | Investment of resources in the rural campus may add an increment to rural practice choice beyond rural upbringing and family medicine residency. |
| Diemer, 2012([5](#_ENREF_5)) | Texas Academy of Physician Assistants  Physician assistants | N=206 respondents (out of 1,997 surveys sent)  Survey  Rural: <50,000 people  Outcome: Rural location as first practice choice after graduation | Significant relationship between rural background and rural practice (p<.003); participants who lived >20 years in a rural community before physician assistant school more frequently chose rural practice compared to those that did not; proximity to friends/colleagues and proximity to relatives was not a particularly influential factor | Significant relationship between rural clerkship and rural practice (p<.001); participation in rural training was not rated as particularly influential | Loan forgiveness/repayment program was not a particularly influential factor | High agreement with serving the health needs of the community, type of practice, supervising physician characteristics; cultural and recreational activities, educational facilities in the community, and community recruitment effort were not a particularly influential factor | N/A | Physician assistants with a rural background and those that completed rural clerkships have a greater propensity toward rural practice. |
| DHHS, 2006([6](#_ENREF_6)) | US, 18 specialties  Physicians | N=N/A  Analytic study  Rural: N/A  Outcome: Working in rural area | Female physicians are less likely to work in rural areas | N/A | N/A | N/A | N/A | The growth and aging of the US population will cause a surge in demand for physician services; if current healthcare utilization and delivery patterns continue, the overall supply of physicians should be sufficient to meet the expected demand through the next 10 years. |
| Duffrin, 2014([7](#_ENREF_7)) | Current members of the North Carolina Medical Board who are listed as primary care physicians  Primary care physicians practicing in family medicine, internal medicine, ob-gyn, general practice, and pediatrics | N=975  Survey  Rural: County of <50,000 people  Outcome: Practicing in rural area and practicing physicians in 2012 | Population of hometown <11,000 was associated with working in non-metro area (p =.007) | N/A | Pay as a factor in choosing a work site, financial support from a hospital, and medical school loan repayment were correlated with rural practice (effect size not reported) | N/A | N/A | Federal and state incentives should continue; having been raised in an area of 11,000 or less was highly predictive of future rural medical practice and could be used in the recruitment of physicians and residents to increase the ultimate yield for rural areas. |
| Fordyce, 2012([8](#_ENREF_8), [9](#_ENREF_9)) | MDs and DOs from 2005 AMA and AOA Masterfiles  Primary care physicians | N=231,660  Analytic study  Rural: RUCA classification (urban, large rural, small rural, or isolated small rural)  Outcome: Practicing in rural areas in 2005 | IMGs comprised 22.2% of total clinically active workforce, but contributed 19.3% to the rural PCP workforce; the proportion of rural PCP workforce represented by IMGs decreased with increasing rurality | DOs comprised 4.9% of clinically active workforce but contributed 10.4% to rural PCP workforce, some geographic variation. DO PCPs were more likely than allopathic PCPs to practice in rural places (20.5% vs 14.9%). Proportion of rural PCP workforce represented by DOs increased with increasing rurality | N/A | N/A | IMG PCPs were more likely than other PCPs to practice in rural persistent poverty locations (12.4% vs 9.1%) | DO and IMG PCPs constitute a vital portion of the rural healthcare workforce; their ongoing participation is necessary in addressing existing rural PCP shortages and handling the influx of newly insured residents as the ACA comes into effect. |
| Glasser, 2010([10](#_ENREF_10)) | Recently located rural physicians and graduates of a rural medical education program in rural Illinois  Physicians | N=107  Interviews  Rural: Illinois Department of Public Health designations of rural areas  Outcome: Practicing in rural county | Major reason for practicing in a rural location was family ties to the community (50%) | N/A | 2nd major reason for practicing in a rural location was a loan or scholarship obligation (30%) | N/A | N/A | Keys to success in rural physician retention seem to include identifying and recruiting medical students of rural origin and focusing on a healthy practice environment; policy makers need to work with local government, schools and employers to offer programs to identify local youth for induction in rural healthcare. |
| Hancock, 2009([11](#_ENREF_11)) | Physicians practicing in rural northeastern California and northwestern Nevada  Primary care physicians | N=22  Interviews  California OSHPD Rural Medical Service Study Areas (density <250 persons per square mile, no census-defined place >50,000)  Outcome: practicing in rural areas in northeastern California and northwestern Nevada (2006-2007) | Rural exposure via upbringing and recreation, and a history of strong community or geographic ties facilitates future rural practice | Rural exposure via education facilitates future rural practice | N/A | N/A | Exposure facilitates through desires for familiarity, sense of place, community involvement, and self-actualization | Results support a focus on recruitment of rural-raised and community-oriented applicants to medical school, residency, and rural practice. Local mentorship and ‘‘place-specific education’’ can support the integration of new rural physicians. |
| Helland, 2010([12](#_ENREF_12)) | Emergency medicine residents who graduated from 2006 to 2008 and practice in rural EDs, and a random sample practicing in urban EDs.  Emergency medicine physicians | N=197  Survey  Rural: Based on US Department of Agriculture county-based Urban Influence Codes  Outcome: Practicing in rural EDs (graduated 2006-2008) | Rural practice location was associated with 18 childhood years in a rural area (42% rural vs 24% urban); important factors reported for choosing practice location included family/spouse (81% rural vs 72% urban) and previous time spent in similar area (61% rural vs 53% urban) | Emergency medicine board certification was associated with rural practice; practice location was not significantly correlated with rural residency rotation | Cost of living, salary signing bonus, and loan repayment were not rated as important. There was a significant difference in ratings between urban and rural providers for the importance of loan repayment | Important factors for choosing practice location included lifestyle (78%), but not access to CME, service to the underserved, autonomy/scope of practice, or access to specialists. 43% vs 56% of rural vs urban providers rated ED volume as very important; 53% vs 68% access to amenities/recreation | Rural and urban physicians reported similar plans for duration of practicing in their type of area for >10 years (57% vs 60%) and for <2 years (6% vs 5%) | Promising strategies for recruiting new residency graduates to rural EDs are selection of individuals with a rural upbringing and higher salaries; increasing the availability of rural rotations during emergency medicine residency also may help to motivate and prepare some new graduates to practice in rural EDs. |
| Heneghan, 2005([13](#_ENREF_13)) | General surgeons practicing in the US  General surgeons | N=421  Survey  Rural: OMB designation, Goldsmith modification  Outcome: Difference in ratings between rural and urban surgeons | N/A | N/A | Reporting income as having a high impact on practice location was lower among rural surgeons (19.8% rural vs 36.1% urban, p = .0002) | The impact of potential for professional growth, availability of hospital facilities, quality of surgical community, and quality of medical community (all factors p<.001) on location preference were rated differently between rural and urban groups Quality of life was not rated as an important factor | N/A | Although rural and urban surgeons do not differ in age or the importance of lifestyle in deciding career location, different factors do impact their choice of location; practice pattern and educational needs varied markedly between rural and urban general surgeons. |
| Henry, 2007([14](#_ENREF_14)) | Physicians assistants (PAs) in Texas who work autonomously in a rural health clinic, sole PCP in community for >24 months. Town with <5,000 persons, no other primary care within 25 miles.  Physican assistants | N=8  Survey  Rural: Town with < 5,000 people  Outcome: Important factor influencing to work in a rural satellite clinic in 2005 | Majority (7/8) did not grow up in small town; confidence to practice without physician was an important factor influencing to work in a rural clinic | N/A | N/A | Desire for small-town life, importance of knowing patients on a personal level, and spouse value of small-town life influenced work in rural clinic | N/A | In order to increase retention rates, PAs committed to autonomous, rural primary care would benefit from additional training, particularly in emergency medicine, the benefits of community involvement, and adaptation to the local culture. |
| Jarman, 2009([15](#_ENREF_15)) | Surgery residents graduates of all 4 Wisconsin programs from 1994-2008  General surgeons and subspecialty surgeons | N=45  Survey  Rural: Rural = population of <50,000 people  Outcome: General surgery practice in rural location 1994-2008 | Factors associated with rural vs urban practice included attending a nonurban high school (p=.001) or college (p=.001), location spouse/ partner grew up (p=.022), and having a child before/during medical school (p=.043). Graduates in an urban setting were more likely to have a parent with a medical occupation (p=.03). Practice location was not associated with sex, birthplace (US-only, rural vs non-rural), parental occupation, having a parent who grew up on a farm, participation in high school or college sports, participation in high school academic club, playing a musical instrument, listening to all types of music, being married, being married during college or before, or having children | Factors associated with rural practice included completing a rural clerkship (p=.001) and having chosen a surgical residency program committed to rural training (p=.046). Factors negatively associated with rural practice included completion of a fellowship (p<.001) and teaching surgical residents (p<.001). Practice location was not associated with clinical research during residency or bench research during residency | N/A | Factors positively associated with rural practice included interest in hunting birds (p=.010) or large game (p=.001). Graduates in rural practice more often cited "broad scope of practice" as an important reason. Practice location was not associated with current hobbies, fishing, hunting small game, happiness with location, spouse's happiness with location, or satisfaction with scope of practice | N/A | General surgery residency graduates and their spouses who choose rural practices are more likely than those selecting urban practices to have rural backgrounds and interests; completing a rural clerkship during medical school and choosing a residency program committed to rural general surgery preparation are strongly correlated with rural practice. |
| Kimball, 2007([16](#_ENREF_16)) | Female physicians practicing in rural Wisconsin  Physicians | N=10  Interviews  Rural: Wisconsin communities <16,000 people  Outcome: Motivation to enter rural practice, reasons for choosing practice location | 70% had a rural background; 60% stated they were always interested in rural practice; Reasons for choosing location included proximity to family (60%) or personal connection to area (60%); 60% stated that family obligations did not influence decision to practice in current community | 20% stated that their medical school had encouraged rural practice, 40% stated that it was discouraged, 40% stated that no specific practice location had been emphasized | N/A | Reasons for choosing practice included liking the community (60%). Good access to specialist backup was listed by only 20%, and full scope of practice by 10% | N/A | The participants provided insight into motivating woman to enter rural practice, finding a balance between the challenges and benefits of rural medicine, and promoting the future of rural healthcare. |
| Mason, 2012([17](#_ENREF_17)) | 1990-1999 UMC graduates practicing in Mississippi (MS) from 2004 MS Board of Medical Licensure  Physicians | N=927  Analytic study  Rural: N/A  Outcome: Practicing in small town in 2009 | Factors not associated: attended high school in MS, attending college in MS, internship in MS, began practice in MS, moved practice to MS, age, sex, race, or marital status | UMC graduates were not more likely to practice in rural areas in MS than physicians who graduated elsewhere. PCPs were 2.4 times (p<.001) more likely to practice in small town areas than specialists (controlling for all other factors) | Salary or student loan debt were not predictors of practicing in small towns (multivariate analysis) | N/A | N/A | Health educators and policy makers should consider broadening the enrollment policies and greater emphasis should be placed on recruiting physicians. |
| Mertz, 2007([18](#_ENREF_18)) | Licenced physicians of South Asian ethnicity in California  Physicians | N=3,862 South Asian respondents (out of 109,763 distributed questionnaires)  Survey  Rural: Geocoded ZIP codes to state-defined medical service study area, population density <250 residents per square mile and no city with >50,000 residents | Odds of South Asian international medical graduates working in a rural community are 1.6 times the odds of South Asian US medical graduates in a multivariate analysis controlling for gender, age, and specialty choice | N/A | N/A | N/A | N/A | Continuing central importance to addressing the needs of medically underserved populations of training physiciants from unter-represented minority groups in US medical schools. |
| Pepper, 2010([19](#_ENREF_19)) | Physicians in Wyoming  All MDs and DOs | N=693  Survey  Rural: 2003 Department of Agriculture RUCC codes 3 to 9  Outcome: Practicing in less-populated county in 2007 | Being raised in a rural area was associated with practicing in a less populated county (p<.05) in a multivariate analysis. There was no association with gender, being raised in a bordering state, completing an internship or residency in a bordering state, or plans to move out of state | There was no association with medical school location | N/A | N/A | N/A | Rural backgrounds and training independently predict practice location decisions. |
| Petrany, 2013([20](#_ENREF_20)) | Marshall University Family Medicine Residency  Physicians | N=106  Rural: N/A  Outcome: Practicing in rural area after completing residency | N/A | The difference rural practice between rural track and traditional track graduates remained significant in a multivariate analysis (OR 7.54, CI 1.5, 37.9) after adjusting for age at entry, gender, West Virginia hometown and academic scores | N/A | N/A | N/A | Rural track graduates are more likelty to practice in rural areas. |
| Phillips, 2009([21](#_ENREF_21)) | US allopathic medical students  Physicians | N=322,131  Analytic study  Rural: RUCA codes  Outcome: Rural practice in Rural Health Clinic (2001-2005) | Rural practice was associated with being born in rural county (OR 2.35), being male (OR 1.49), married (OR 1.47), age at graduation (OR 1.03). Rural practice was associated with plans to serve in underserved areas (RR 3.40) | Rural practice was associated with attending medical school in rural area (OR 2.93), career in family medicine (OR 2.65), as well as attending a public medical school (OR 1.66), community related medical school (OR 1.20), and experience in Title VII funded school (OR 1.11). Rural practice was associated with practice taking a rural (RR 1.9) or community health (RR 1.63) elective, family medicine clerkship (RR 1.44), experience with a Title VII school (RR 1.31), primary care residency (RR 1.22) | Rural practice was associated with NHSC loan repayment (OR 2.06), NHSC scholarship (OR 1.88), medical school debt $200-250K (OR 1.34), medical school debt $150-200K (OR 1.24), medical school debt $100-150K (OR 1.29), medical school debt $50-100K (OR 1.19), and medical school debt $1-50K (OR 1.06) | N/A | N/A | If rural–born students interested in serving the underserved also have rural training experience, it may have “multiples of effect”. Schools, residency programs, and medical education funders should consider this. Schools should institute a series of interview questions about rural and other underserved patients and should give these weight in acceptance. They could also become markers for targeted mentoring and training experiences. |
| Phillips, 2016([22](#_ENREF_22)) | US Female family physicians from snowball sample  Family physicians | N=25  Interviews  Rural: RUCA 7 or higher  Outcome: Rural practice in 2012 | 60% of interviewees had lived in a rural community before age 18; many interviewees had sought out life partners who were willing to live in a rural community | Many interviewees had developed an interest in rural medicine before or during medical school | N/A | Most participants had chosen to practice in a rural community, in part, because they could maintain a broad scope of practice | N/A | Women family physicians can build successful careers in rural communities, but suportive employers, relationships, and patient approaches rpovide a foundation for this success |
| Rabinowitz, 2012([23-26](#_ENREF_23)) | MD graduates from Jefferson Medical College  Physicians | N=3,006  Analytic study  Rural: Rural county = 2007 Rural-Urban Density Typology (RUDT)  Outcome: Practicing in rural area in 2007 | 3 predictors of rural practice (p<.001): growing up in a rural area, entering medical school with plans for rural practice, and entering medical school with plans to be a family physician. Of graduates with all predictors, 45% practiced in rural areas; of those with 2, 33%; of those with 1, 1%; and of those with 0, 12% practiced in rural areas. The RR for rural practice was 3.9 (CI 2.7-5.7, p<.001) for those with 3 predictors, 2.9 (CI 2.0-4.2, p<.001) for those with 2 predictors, and 1.8 (CI 1.2-2.8, p<.01) for those with 1 predictor. Medical students' specialty plans were strongly related to rural practice (p<.001) | N/A | N/A | N/A | N/A | Three factors known at the time of medical school matriculation have a powerful relationship with rural practice 3 decades later; relatively few students without predictors practice in rural areas, which is particularly significant given subsequent factors known to be related to rural practice – for instance, rural curriculum, residency location or spouse. |
| Renner, 2010([27](#_ENREF_27)) | Colorado healthcare providers who participated in loan repayment program  Healthcare providers (e.g. physicians, nurse practitioners) | N=93  Survey  Rural: ZIP code with RUCA designation above and including 4.0  Outcome: Practicing in rural care in 2007 | Rural providers were more likely to have gone to a rural high school than urban providers (38% vs 9%, p=.007) | N/A | 58% reported that salary was an important factor. Signing bonus, amount of loan repayment, and other incentives were less important. 42% reported the loan program had an important influence on the specific community they chose to practice | Rural providers rated location (83%), scope of practice (79%), and family fit with community (73%) as the most important factors. School opportunity for children were rated less important for rural providers | N/A | Loan repayment programs targeting rural Colorado usually enroll providers who would have worked in a rural area regardless of loan repayment opportunities, but are likely to play a role in provider's choice of specific rural community for practice. |
| Schiff, 2012([28](#_ENREF_28)) | Physicians practicing in Hawaii who graduated from the University of Hawaii School of Medicine from 1993-2006  Physicians (all specialties) | N=177  Analytic study  Rural: O‘ahu considered urban; all other islands rural  Outcome: Practicing in rural settings in Hawaii (1993-2006) | Hawaii-schooled physicians who attended rural high schools were 9x more likely to practice in a rural location than those who went to high school on a neighbor island (p<.0001) | No significant association between rural practice and primary care specialty (p=.09) | N/A | N/A | N/A | If the State of Hawaii wants to expand the physician workforce in the rural areas of Hawaii, recruiting more students from rural areas is an excellent path to take. |
| Shannon, 2011([29](#_ENREF_29)) | Physician assistants in West Virginia who completed a rural rotation during clinical education  Physician assistants | N=168  Analytic study  Rural: ZIP code approximation of the RUCA code classification  Outcome: Rural practice in West Virginia in 2005-2010 | Using gender, school, student evaluation of rural field experience, change in interest in rural health, confidence in community activities, confidence in meeting the needs of rural populations, rural high school hometown, rural practice intent, likelihood of WV practice, only the variables rural high school hometown and likelihood of WV practice correctly predicted rural practice; 77% of students who predicted rural practice were in rural practice, 63% who did not predict it were in rural practice (p<.04) | N/A | N/A | Confidence in community activities, and confidence in meeting the needs of rural populations, did not predict rural practice | N/A | This study suggests moderate predictive validity of PA student reporting on rural practice and on West Virginia practice intent; such methods may have potential in prediction of the future rural PA workforce. |
| Shannon, 2015([30](#_ENREF_30)) | West Virginia medical student questionnaires 2001-2009  Medical students | N=1,517  Survey  Rural: 2006 Zip code approximations RUCA 2.0 codes  Outcome: 2013 rural practice site | Rural hometown was a predictor of rural practice in a multivariate analysis OR 4.02 (CI 2.17, 7.74) | N/A | N/A | N/A | Significant associaton between prerotation (p<.01) and postrotation (p<.01) questionnaire prediction | The study demonstrates the utility of medical students questionnaires for projections of numbers of future rural physicians |
| Smith, 2012([31](#_ENREF_31)) | US  Physician assistants | N=312  Survey  Rural: < = 50,000 people  Outcome: Rural setting for first practice in 2009 | Respondents who graduated from a rural high school and singles were significantly more likely to practice in a rural setting (both p<.05). No significant difference between rural practice and degree, race, gender, and age at graduation. Support of and for significant other was the most important factor for first practice location | Specialty distribution (primary care, specialty, other) was significantly different between urban and rural groups (p<.05) | N/A | N/A | Six factors emerged from factor analysis: hours of work/ compensation, support of/for significant other, community and job amenities, educational resources /access to care, practice opportunities, and location | Respondents felt that support of and for the significant other was the most important factor in their first practice-location choice; recruiters may wish to pay closer attention to spousal opportunities and should not underestimate the impact of family in the decision about work location. |
| Snyder, 2014([32](#_ENREF_32)) | PAs actively practicing in Indiana who graduated from 2000-2010, and had email addresses available.  Physician assistants | N=157  Survey  Rural: Respondent-defined  Outcome: Location of initial job, location of current job (2000-2010) | N/A | 71% indicated educational dept had no influence on location of initial job | 72% indicated dept had no influence on initial location decision | N/A | 34% of urban PAs would have practiced in a rural area if they had received federal or state loan forgiveness for educational debt, 30% would have reconsidered | This study provides evidence that debt may influence practice specialty and location choice. Further studies are needed to determine how gender might account for decisions to practice in certain specialties and location. |
| Stenger, 2008([33](#_ENREF_33)) | Massachusetts physicians practicing in areas of the state designated as rural from 2004-2005  Practicing physicians | N=160  Survey  Rural: Non-metropolitan county designated non-urban by RUCA or population < 10,000 and population density < 500 people per square mile  Outcome: Practicing in rural area in 2004-2005 | Most rural physicians (73.2%) had grown up in larger towns with populations of >10,000 | N/A | N/A | Responses to why remaining in rural practice included feeling established with a strong sense of connection to patients and place, overall satisfaction with practice, and being in a great place to live | Factors associated with higher satisfaction included not feeling overworked (p = .043), or professionally isolated (p = .004), and being involved in practice (p = .045) and community (p = .036) | The findings reaffirm the importance of rural medical education opportunities in physician recruitment, retention, and practice satisfaction and indicate that a major source of physicians for rural and small town communities is physicians who have been raised in urban/suburban communities and who were trained outside of the region but who were prepared to live and to practice in rural and small town communities. |
| Terhune, 2010([34](#_ENREF_34)) | Active nonresident surgeons, in AMA masterfile 2008-2009  General surgeons | N=17,727  Analytic study  Rural: RUCA  Outcome: 2009 practice location | 15.1% of international medical graduates (IMG) worked in rural areas compared to 17% of non-IMGs (p<0.001) | N/A | N/A | N/A | N/A | Current numbers of IMGs in practice are declining. |
| Wendling, 2016([35](#_ENREF_35)) | Michigan State University College of Human Medicine graduates 1978-2006; AMA masterfile data  Practicing physicians | N=2,382  Analytic study  Rural: Geocoded locations to ZIP code, RUCA  Outcome: Practicing in rural area in 2011 | Rural origin (OR 2.80 (CI 2.09, 3.74),and male gender (OR 1.39 (CI 1.10, 1.75) were significant predictors of rural practice in a multivariate analysis | A rural campus (OR 3.09 (CI 2.12, 4.50) and primary care specialty (OR 1.65 (CI 1.31, 2.08) were significant predictors of rural practice in a multivariate analysis | N/A | N/A | Percentage of graduates practicing rurally has increased each decade | Program characteristics, including targeting rurally interested students, primary care focus, and substantial clincial training within a rural region, can successfully foster rural physician careers. |
| Whitacre, 2010([36](#_ENREF_36)) | Medical students between 1997-2002 at Oklahoma State University College of Osteopathic Medicine  Medical students | N=190  Analytic study  Rural: population < 50,000  Outcome: Rural practice location in 2008 | Being raised in a rural area was associated with rural practice (p<.05) | Rural practice was associated with summer rural externship (p<.05), but not with all early clinical experiences | N/A | N/A | N/A | Programs implemented by an osteopathic school can influence where graduates choose to practice; programs taking place in both the 1st and 4th year of training have an impact on rural practice location, implying that students can be influenced both early and late in their medical school careers. |
| Zink, 2010([37](#_ENREF_37)) | Medical students at 2 University of Minnesota campuses that did or did not participate in a rural training program  Medical students | N=3,365  Analytic study  Rural: OMB’s definitions; communities not listed were considered metropolitan if within 50 miles of an urban center, otherwise designated as rural  Outcome: Rural practice in 2008 | In a multivariate analysis, rural practice was associated with being raised in a rural community (OR 2.82, CI 2.1-3.79) | In multivariate analysis, rural practice was associated with participating in a rural program (OR 4.62, CI 3.01, 7.07) and training at Duluth campus (rural focus) (OR 4.09, CI 2.81, 5.96) | N/A | N/A | N/A | RPAP and UMN-Duluth provide significant, complementary educational programs that lead more graduates to choose rural and primary care practices; efforts across the nation to address the crisis in rural primary care should build on these successful efforts. |

Note: AMA = American Medical Association, AOA = American Osteopathic Association, CI = 95% confidence interval, CME = Continuing medical education, DHHS = Department of Health and Human Services, DO = Doctor of osteopathic medicine, ED = emergency department, IMG = International medical graduates, MD = allopathic medical doctor, MS = Mississippi, NHSC = National Health Service Corps, Ob-gyn = obstetricians and gynecologists, OR = odds ratio, OSHPD = Office of Statewide Health Planning and Development, PA = physician’s assistant, PCP = primary care provider, OMB = Office of Management and Budget, RR = risk ratio, RUCA = Rural-Urban Commuting Area, RUCC = Rural-Urban Continuum Codes, vs = versus, WV = West Virginia

### Evidence Table Training Success

| **Study** | **Institution/Location**  **Graduation period** | **Providers in training**  **N**  **Data source** | **Program description**  **Capacity/Avg participation** | **Study design**  **Definition of rural** | **Recruitment results** | **Retention results** |
| --- | --- | --- | --- | --- | --- | --- |
|  | **Evaluations of programs at a single institution** |  |  |  |  |  |
| Antonenko, 2009([38](#_ENREF_38)) | North Dakota University School of Medicine and Health Sciences  1986 - 2008 | General surgery residents  N=44  Internal records | Rotation on Indian reservation  Capacity: 2-3 residents/year  Duration; 1 month per year for 2 years (total 2 months)  Selection: All residents participate | Post-only  Rural: N/A | 41% of graduates continue to practice in rural areas | NA |
| Bonham, 2014([39](#_ENREF_39)) | University of New Mexico Rural Psychiatry Residency Program (RPRP)  1991-2010 | Psychiatry residents  N=60 RPRP graduates, 131 traditional program graduates  Internal records | 8-week seminar, rural site visits, 2 days/week in rural setting during PGY-4 year  Participation: 3 residents/year  Duration: 2 months  Selection: All residents participate | Comparative  Rural: Large rural town (pop. 10,000–49,999), small rural town (pop. 2,500–9,999) | 37% of RPRP graduates practice in rural communities compared to 10% of traditional program graduates | NA |
| Crane, 2014([40](#_ENREF_40)) | North Carolina Hendersonville Family Medicine Residency Program  1999 - 2013 | Family medicine residents  N=37  Internal records | Rural-track training program in rural North Carolina  Capacity: 2-4 residents/year  Duration: NR, residency program  Selection: NR | Post-only  Rural: N/A | 65% graduates practice in rural communities | NA |
| Crump, 2010([41](#_ENREF_41)) | Kentucky University of Louisville School of Medicine Trover Campus  2003-2006 | Medical students  N=24  Internal records | Preclinical rural medicine elective  Capacity: N/A  Duration: 16 contact hour elective, met for once per month for 2.5 hours for 9 months  Selection: None (self-select), 98% from rural towns | Post-only  Rural: N/A | 29% of students practice in rural Kentucky | N/A |
| Crump, 2016([3](#_ENREF_3), [42](#_ENREF_42)) | Kentucky University of Louisville School of Medicine Trover Campus  2001-2008 | Medical students  N=1,120  AMA Master File | Rural clinical campus  Capacity: N/A  Duration: 24 months (unclear)  Selection: None (self-select) | Comparative  Rural: Nonmetro RUCC codes | 45% of rural campus graduates practice in rural locations compared to 7% of standard campus graduates | N/A |
| Deveney, 2009([43](#_ENREF_43), [44](#_ENREF_44)) | Oregon Health Sciences University  2002-2012 | General surgery residents  N=11 rural track graduates, 59 traditional track graduates  Internal records | Year-long 4^th^ year rotation in rural hospital  Capacity: 2 residents/year  Duration: 12 months  Selection: N/A | Comparative  Rural: site with population <50,000 | 36% of graduates who completed the rural rotation subsequently practice in rural communities, compared to 7% of all graduates | NA |
| Gillig, 2009([45](#_ENREF_45)) | Ohio Wright State University Dayton, Psychiatry | Psychiatry residents  N=50  Internal records | Community psychiatry curriculum for 2^nd^ year students, attending rural clinic for 1 day every other week  Capacity: N/A  Duration: 1 day every other week for 12 months  Selection: None (all residents participate) | Pre-post  Rural: N/A | 28% of residents selected jobs at rural sites in the state; before the program, no graduates had selected careers in rural sites | N/a |
| Hulme, 2014([46](#_ENREF_46)) | South Dakota State University | Nurse practitioner students  N=9 respondents (out of 12)  Survey | Integrated family / psychiatric-mental health care nurse practitioner program with emphasis on rural practice  Capacity: 2-7 students  Duration: 36 months of full time study (855 clinical hours)  Selection: marketed program to nurses, assessed on their commitment to rural and underserved populations | Post-only  Rural: N/A | 44% of respondents practice in rural areas | N/A |
| Kallail, 2010([47](#_ENREF_47)) | University of Kansas School of Medicine  1997-N/A | Premedical students with assured admission to medical school  N=19  Internal database | Scholars in rural health program is designed to attract and retain rural Kansans  Capacity: 14 students per year  Duration: 24 months pre-medical curriculum  Selection: Scholars from rural Kansas or underserved urban areas | Post-only  Rural: Based on population density (either frontier, rural, or dense rural) | 63% of graduates practice in rural, non-metropolitan communities | N/A |
| Nash, 2008([48](#_ENREF_48)) | University of Texas Medical Branch at Galveston  2000-2007 | Family medicine residents  N=7  Internal records | 28-week longitudinal rural rotation, optional rural electives  Capacity: 2-3 residents/year  Duration: 7 months training in rural community  Selection: N/A | Post-only  Rural: N/A | 86% of graduates enter practice in rural areas | NA |
| Petrany, 2013([20](#_ENREF_20)) | West Virginia Marshall University Family Medicine Residency  1994-2006 | Family medicine residents  N=106 (12 rural track students)  Internal records | Rural track, residents provide care at a rural community health center  Capacity: N/A  Duration: 36 months (starting with 0.5 to then 4.5 half days)  Selection: N/A | Comparative  Rural: N/A | 83% of rural track graduates practiced in a rural area compared with 40% of the traditional track (p<.01) | N/A |
| Quinn, 2011([49](#_ENREF_49)) | University of Missouri School of Medicine  2002-2009 | Medical students  N=344  Internal records, National Residency Match Program, American Board of Medical Specialties | Rural Track Pipeline Program: preadmission program; summer community program; rural track clerkship; rural track elective  Capacity: Preadmission program: 15; summer community program: 20-30; rural track clerkship: 20-35; rural track elective: 5-7 students/year  Duration: 6 months rural track clerkship  Selection Interview assessed students’ rural identity and intent to practice rural medicine | Post-only  Rural: Population density thresholds of the US Census Bureau | Preadmission program: 50%, clerkship only: 64%; clerkship + summer program or rural track elective: 56%, any pipeline program component: 57% report first practice location to be rural or mixed rural | N/A |
| Ross, 2013([50](#_ENREF_50)) | Cascades East Family Medicine Residency,  Klamath Falls, Oregon  1994 - 2009 | Family medicine residents  N=62 (82% response rate)  Survey | Residency training program in Klamath Falls (population ~42,000)  Mean participation: 4.8 residents/year  Duration: 36 month residency training program  Selection: NR | Post-only  Rural: Population <25,000, >25 miles from major centers | 60% of graduates enter practice in communities of < 25,000 residents and 45% enter practice in towns of less than 10,000 | 50% of graduates remained in rural practice in 2009 |
| Wendling, 2016([35](#_ENREF_35)) | Michigan State University College of Human Medicine  1978-2006 | Medical students  N=2778 (168 rural track)  AMA Masterfile 2011 | Rural physician program, all clinical training in nonmetropolitan area  Capacity: 12 students/year  Duration: 24 months  Selection: Based on interest in rural health, rural life experience, and program fit | Comparative  Rural: Geocoded locations to ZIP code, RUCA | 45% of rural training program graduates practice in a rural community compared to 14% of other clinical campuses (p<0.001) | N/A |
| Whitacre, 2010([36](#_ENREF_36)) | Oklahoma State University  1997-2002 | Osteopathic medical students  N=190  Source N/A | Mandatory and optional programs: summer rural externship, early clinical experience, community clinic clerkship (mandatory), rural clinc clerkship (mandatory) community hospital rotations (mandatory)  Capacity: N/A  Duration: Optional: summer rural externship: 30-35 hrs per week for 1 month; early clinical experience: 5 day tour. Mandatory: community clinic clerkship: 1 month rotation; rural clinic clerkship: 1 month rotation; community hospital rotations: 2 1 month rotations (total 2 months).  Selection: NR, some programs mandatory | Post-only  Rural: Community with population <50,000 | 30% of graduates chose to practice in a rural location in 2008; the optional summer program increased the probability by 24% | N/A |
| Wick, 2015([51](#_ENREF_51)) | University of Washington  1991-2013 | Physician assistants, international medical graduates  N=39  Internal records | Physician assistant program for international medical graduates to prepare for primary care, midically underserved areas, and rural locations  Capacity: N/A  Duration: Unclear  Selection: (International medical graduates analyzed) | Post-only  Rural: RUCA ZIP codes | 8% of the international medical graduates practice in rural settings | N/A |
| Zink, 2010([37](#_ENREF_37), [52](#_ENREF_52), [53](#_ENREF_53)) | University of Minnesota  1990-2004 | Medical students  N=3365  Internal records, AMA Masterfile | Rural Physician Associate Program, Twin City campus  Capacity: 33 students per class  Duration: 9 months  Selection: NR for rural program, for rural medical school, students from rural areas and those who are likely to practice in rural areas are given preference | Comparative study  Rural: Office of Management and Budget’s definitions of metropolitan and nonmetropolitan populations. Communities that were not listed were considered metropolitan if within 50 miles of an urban center, otherwise designated as rural | 31% of the program graduates practice in rural care in 2007 compared to 9% of non-participants at the same campus | N/A |
|  | **Evaluations across multiple training institutions** |  |  |  |  |  |
| Deutchman, 2013([54](#_ENREF_54)) | 35 US medical schools with established rural tracks or rural tracks in development, | Medical students  N=N/A  Information collected and provided by each Rural Track program | 38 rural tracks  Capacity: The number of students participating in each RT ranges from 4-60, with the majority representing 5% to 10% of each class at that institution  Duration: NR  Selection: Of 34 rural tracks with data on selection criteria, 26 preferred students planning on practicing primary care and 20 preferred students planning on practicing family medicine | Post-only  Rural: Defined by presence of Rural Track site | The 18 programs that have been able to track students' practice locations report that an average of 44% of their graduates practice in a rural area (range: 20-73%) | N/A |
| Patterson, 2013([55](#_ENREF_55)) | Multiple family medicine residencies with Rural Training Tracks supported by Technichal Assistance Program  2008-2012 | Family medicine residents  N=123 (location data available for 64)  Survey of rural training track Programs, AMA Master file, ABFM, and CMS. | Rural training tracks combine one year of urban training with two years of rural training.  Capacity: 1.5 residents/year per program  Duration: NR  Selection: NR | Post-only  Rural: ZIP codes | 72% began clinical practice in rural areas | At 3 years there was migration away from rural areas; 61% remained in rural practice |
| Phillips, 2009([21](#_ENREF_21)) | US allopathic medical students  1978-2005 | Medical students  N=322,131  AAMC medical school graduation questionnaire, AMA physician masterfile | Since 1976, Title VII funding has provided substantial support for Family Medicine educational programs at medical schools in many states. These curricula focus on development of primary care physicians who would care for urban and rural underserved populations  Capacity: N/A  Duration: NR  Selection: NR | Post-only  Rural: RUCA codes | Title VII-funded school experience increases likelihood of rural practice (OR 1.11 sign.) but not Title VII-funded residency (OR 0.90, sign.); relative likelihood of association between Title VII funding and practice in rural area (RR 1.31, sign.) and practice in rural health center (RR 1.20, sign.). Practice in rural area was associated with predoc Title VII training funding (RR 1.39, sign.) and matriculated in Title VII funded school (RR 1.31, sign.) | N/A |
| Phillips, 2013([56](#_ENREF_56)) | Critical Access Hospitals (CAHs), Rural Health Centers (RHCs), and Federally Qualified Health Centers)  2001-2005 | Residents who trained, at least in part, at a CAH, RHC, or FQHC  N=3,430  Medicare claims 2001-2005, 2009 & 2011 AMA Masterfile | CAHs, RHCs and FQHCs are safety net settings. CAHs and RHCs are typically located in rural areas  Capacity: N/A  Duration: N/A  Selection: N/A | Post-only  Rural: CAH or RHC | 48% of the residents who trained at a CAH, 35.6% who trained at an RHC, and 18.6% who trained in an FQHC later practiced in either a CAH or an RHC | NA |
| Rabinowitz, 2011([10](#_ENREF_10), [57-62](#_ENREF_57)) | Jefferson Medical College Physician Shortage Area Program; University of Minnesota Medical School, Duluth; and University of Illinois College of Medicine Rockford Rural Medical Education Program  1976-2005 | Rural medical school graduates  N=1,551  AMA Masterfile 2010 | Comprehensive medical school rural programs  Capacity: NA  Duration: Range from 1.5 to 24 months  Selection: Preferential admission of students likely to practice primary care in rural areas | Post-only  Rural: Rural Urban Density Typology | 61% of graduates were practicing in a rural area in 2010 | 70% of Jefferson graduates who initially (in 1986 or 1991) practiced in rural family medicine, were still practicing family medicine in the same rural area. 82% of Rockford graduates have stayed at their original practice site, 8 graduates have relocated to rural communities |
| Shipman, 2013([63](#_ENREF_63)) | US MD granting medical schools  1999-2011 | Medical students  N=135 medical schools  AMA Masterfile and AMCAS | Comparison of graduates prior to expansion (1999-2001) to 10 years after expansion (2009-2011); in 2006, AAMC had called for a 30% increase in MD-granting medical school enrollment  Capacity: 3,500 additional students yearly  Duration: NR  Selection: NR | Pre-post  Rural: RUCC to classify the county as nonrural (codes 0-5) or rural (codes 6-9) | Schools with higher growth rates were more likely than schools with lower or no growth to produce graduates practicing in rural areas, range between 5.6 and 8.6% (p<.001) | N/A |
| Talley, 2011([64](#_ENREF_64)) | Rural rotation sites nationwide  2006-2009 | Emergency medicine residents  N=197 completed a rural rotation  111/126 residency programs responded (88% response rate) | Required or elective rural rotations  Capacity: NA  Duration: NR  Selection: NR | Post-only  Rural: Defined by individual programs | 22% of students in required rural rotation programs chose an initial job in rural locations, 7% of programs with elective rotations and predesignated sites, 6% with other elective rotations, and 7% without rural rotations | NA |

Notes: AAMCM = American Association of Medical Colleges, AMA = American Medical Association, AMCAS = American Meical College Application Service

## Risk of Bias

| **ID** | **Selection bias** | **Attrition bias** | **Confounding variables** | **Detection bias (data source) or other limitations** |
| --- | --- | --- | --- | --- |
| Antonenko, 2009([38](#_ENREF_38)) | High risk | N/A | N/A |  |
| Baker, 2012([1](#_ENREF_1)) | N/A | Low risk | Low risk |  |
| Bonham, 2014([39](#_ENREF_39)) | High risk | N/A | N/A |  |
| Chen, 2010([2](#_ENREF_2)) | N/A | Unclear | High risk |  |
| Crane, 2014([40](#_ENREF_40)) | High risk | N/A | N/A |  |
| Crump, 2010([41](#_ENREF_41)) | Low risk | N/A | N/A |  |
| Crump, 2016([3](#_ENREF_3)) | Low risk | Low risk | Low risk |  |
| Deutchman, 2013([54](#_ENREF_54)) | High risk | N/A | N/A |  |
| Deveney, 2009([43](#_ENREF_43), [44](#_ENREF_44)) | High risk | N/A | N/A |  |
| Diemer, 2012([5](#_ENREF_5)) | N/A | High risk | High risk |  |
| DHHS, 2006([6](#_ENREF_6)) | N/A | Unclear | High risk |  |
| Duffrin, 2014([7](#_ENREF_7)) | N/A | High risk | High risk |  |
| Fordyce, 2012([8](#_ENREF_8)) | N/A | Unclear | High risk |  |
| Gillig, 2009([45](#_ENREF_45)) | High risk | N/A | N/A | Imprecision (“of about 50”) |
| Glasser, 2010([10](#_ENREF_10)) | High risk | Low risk | High risk |  |
| Hancock, 2009([11](#_ENREF_11)) | High risk | High risk | High risk | Recall bias and small sample size with likely selection bias |
| Helland, 2010([12](#_ENREF_12)) | N/A | Low risk | High risk |  |
| Heneghan, 2005([13](#_ENREF_13)) | High risk | High risk | High risk | Selection bias, response bias, did not address non-responders |
| Henry, 2007([14](#_ENREF_14)) | N/A | High risk | High risk | Qualitative results only |
| Hulme, 2015([46](#_ENREF_46)) | High risk | High risk | N/A |  |
| Jarman, 2009([15](#_ENREF_15)) | N/A | High risk | High risk |  |
| Kallail, 2010([47](#_ENREF_47)) | High risk | N/A | N/A | Pre-med efforts described in detail but components for accepted studies unclear |
| Kimball, 2007([16](#_ENREF_16)) | High risk | Low risk | High risk | Qualitative study with selection bias |
| Mason, 2012([17](#_ENREF_17)) | High risk | Unclear | Unclear |  |
| Mertz, 2007([18](#_ENREF_18)) | N/A | High risk | Low risk |  |
| Nash, 2008([48](#_ENREF_48)) | High risk | N/A | N/A |  |
| Patterson, 2013([55](#_ENREF_55)) | Unclear | N/A | N/A |  |
| Pepper, 2010([19](#_ENREF_19)) | N/A | Low risk | High risk |  |
| Petrany, 2013([20](#_ENREF_20)) | Unclear | Unclear | Low risk |  |
| Phillips,2009([21](#_ENREF_21)) | Low risk | Unclear | Low risk |  |
| Phillips, 2013([56](#_ENREF_56)) | Unclear | N/A | N/A |  |
| Phillips, 2016([22](#_ENREF_22)) | N/A | High risk | High risk |  |
| Quinn, 2011([49](#_ENREF_49)) | Low risk | N/A | N/A |  |
| Rabinowitz, 2012([23](#_ENREF_23)) | N/A | Low risk | High risk |  |
| Rabinowitz, 2011([57](#_ENREF_57)) | Low risk | N/A | N/A |  |
| Renner, 2010([27](#_ENREF_27)) | N/A | Low risk | Unclear |  |
| Ross, 2013([50](#_ENREF_50)) | High risk | N/A | N/A |  |
| Schiff, 2012([28](#_ENREF_28)) | N/A | Low risk | High risk |  |
| Shannon, 2011([29](#_ENREF_29)) | N/A | Unclear | Low risk | Use of self-reported data, limited sample size, and limited external validity |
| Shannon, 2015([30](#_ENREF_30)) | N/A | Unclear | Low risk |  |
| Shipman, 2013([63](#_ENREF_63)) | Low risk | N/A | N/A |  |
| Smith, 2012([31](#_ENREF_31)) | N/A | High risk | High risk |  |
| Snyder, 2014([32](#_ENREF_32)) | N/A | High risk | High risk |  |
| Stenger, 2008([33](#_ENREF_33)) | N/A | Low risk | Unclear | Self-reported data, selection bias |
| Talley, 2011([64](#_ENREF_64)) | Low risk | N/A | N/A |  |
| Terhune, 2010([34](#_ENREF_34)) | Low risk | Low risk | N/A | Reports statistically significant difference but analysis model not described |
| Wendling, 2016([35](#_ENREF_35)) | High risk | Low risk | Low risk | Not ITT (“we removed 11 graduates”) |
| Whitacre, 2010([36](#_ENREF_36)) | N/A | Unclear | Low risk |  |
| Wick, 2015([51](#_ENREF_51)) | High risk | N/A | N/A | Reports on a subgroup only |
| Zink, 2010([37](#_ENREF_37)) | Unclear | Unclear | Unclear |  |

### References of Included Studies (main publication and companion papers)

1. Baker HH, Pathman DE, Nemitz JW, Boisvert CS, Schwartz RJ, Ridpath LC. Which U.S. medical schools are providing the most physicians for the Appalachian region of the United States? Academic medicine : journal of the Association of American Medical Colleges. 2012;87(4):498-505.

2. Chen F, Fordyce M, Andes S, Hart LG. Which medical schools produce rural physicians? A 15-year update. Academic medicine : journal of the Association of American Medical Colleges. 2010;85(4):594-8.

3. Crump WJ, Fricker RS, Ziegler CH, Wiegman DL. Increasing the Rural Physician Workforce: A Potential Role for Small Rural Medical School Campuses. The Journal of rural health : official journal of the American Rural Health Association and the National Rural Health Care Association. 2016;32(3):254-9.

4. Ziegler C. The association of medical student debt on choice of primary care specialty and rural practice location 2015. Available from: <http://dx.doi.org/10.18297/etd/2024>.

5. Diemer D, Leafman J, Nehrenz GM, Sr., Larsen HS. Factors that influence physician assistant program graduates to choose rural medicine practice. The journal of physician assistant education : the official journal of the Physician Assistant Education Association. 2012;23(1):28-32.

6. US Department of Health and Human Services. Physician supply and demand: Projections to 2020 2006. Available from: <http://bhpr.hrsa.gov/healthworkforce/supplydemand/medicine/physician2020projections.pdf>.

7. Duffrin C, Diaz S, Cashion M, Watson R, Cummings D, Jackson N. Factors associated with placement of rural primary care physicians in North Carolina. Southern medical journal. 2014;107(11):728-33.

8. Fordyce MA, Doescher MP, Chen FM, Hart LG. Osteopathic physicians and international medical graduates in the rural primary care physician workforce. Family medicine. 2012;44(6):396-403.

9. Fordyce MA, Chen FM, Doescher MP, Hart LG. 2005 physician supply and distribution in rural areas of the United States [Internet]. Seattle, WA: Rural Health Research and Policy Centers, 2007.

10. Glasser M, MacDowell M, Hunsaker M, Salafsky S, Nielsen K, Peters K, et al. Factors and outcomes in primary care physician retention in rural areas. 2010.

11. Hancock C, Steinbach A, Nesbitt TS, Adler SR, Auerswald CL. Why doctors choose small towns: a developmental model of rural physician recruitment and retention. Social science & medicine (1982). 2009;69(9):1368-76.

12. Helland LC, Westfall JM, Camargo CA, Jr., Rogers J, Ginde AA. Motivations and barriers for recruitment of new emergency medicine residency graduates to rural emergency departments. Annals of emergency medicine. 2010;56(6):668-73.

13. Heneghan SJ, Bordley Jt, Dietz PA, Gold MS, Jenkins PL, Zuckerman RJ. Comparison of urban and rural general surgeons: motivations for practice location, practice patterns, and education requirements. Journal of the American College of Surgeons. 2005;201(5):732-6.

14. Henry LR, Hooker RS. Retention of physician assistants in rural health clinics. The Journal of rural health : official journal of the American Rural Health Association and the National Rural Health Care Association. 2007;23(3):207-14.

15. Jarman BT, Cogbill TH, Mathiason MA, O'Heron CT, Foley EF, Martin RF, et al. Factors correlated with surgery resident choice to practice general surgery in a rural area. Journal of surgical education. 2009;66(6):319-24.

16. Kimball EB, Crouse BJ. Perspectives of female physicians practicing in rural Wisconsin. WMJ : official publication of the State Medical Society of Wisconsin. 2007;106(5):256-9.

17. Mason PB, Cossman JS. Does one medical school's admission policy help a rural state "grow their own" physicians? Journal of the Mississippi State Medical Association. 2012;53(9):284-6, 8-92.

18. Mertz E, Jain R, Breckler J, Chen E, Grumbach K. Foreign versus domestic education of physicians for the United States: a case study of physicians of South Asian ethnicity in California. Journal of health care for the poor and underserved. 2007;18(4):984-93.

19. Pepper CM, Sandefer RH, Gray MJ. Recruiting and retaining physicians in very rural areas. The Journal of rural health : official journal of the American Rural Health Association and the National Rural Health Care Association. 2010;26(2):196-200.

20. Petrany SM, Gress T. Comparison of academic and practice outcomes of rural and traditional track graduates of a family medicine residency program. Academic medicine : journal of the Association of American Medical Colleges. 2013;88(6):819-23.

21. Phillips RL, Dodoo MS, Petterson S, Xierali I, Bazemore A, Teevan B, et al. Specialty and geographic distribution of the physician workforce: what influences medical student & resident choices? March 2, 2009. Washington, DC: The Robert Graham Center. Available at <http://www.graham-center.org/online/etc/medialib/graham/documents/publications/mongraphs-books/2009/rgcmo-specialty-geographic.Par.0001.File.tmp/Specialty-geography-compressed.pdf>, 2009.

22. Phillips J, Hustedde C, Bjorkman S, Prasad R, Sola O, Wendling A, et al. Rural Women Family Physicians: Strategies for Successful Work-Life Balance. Ann Fam Med. 2016;14(3):244-51.

23. Rabinowitz HK, Diamond JJ, Markham FW, Santana AJ. The relationship between entering medical students' backgrounds and career plans and their rural practice outcomes three decades later. Academic medicine : journal of the Association of American Medical Colleges. 2012;87(4):493-7.

24. Rabinowitz HK, Diamond JJ, Markham FW, Santana AJ. The relationship between matriculating medical students' planned specialties and eventual rural practice outcomes. Academic medicine : journal of the Association of American Medical Colleges. 2012;87(8):1086-90.

25. Rabinowitz HK, Diamond JJ, Markham FW, Santana AJ. Increasing the supply of rural family physicians: recent outcomes from Jefferson Medical College's Physician Shortage Area Program (PSAP). Academic medicine : journal of the Association of American Medical Colleges. 2011;86(2):264-9.

26. Rabinowitz HK, Diamond JJ, Markham FW, Santana AJ. Increasing the supply of women physicians in rural areas: outcomes of a medical school rural program. Journal of the American Board of Family Medicine : JABFM. 2011;24(6):740-4.

27. Renner DM, Westfall JM, Wilroy LA, Ginde AA. The influence of loan repayment on rural healthcare provider recruitment and retention in Colorado. Rural and remote health. 2010;10(4):1605.

28. Schiff T, Felsing-Watkins J, Small C, Takayesu A, Withy K. Addressing the physician shortage in Hawai'i: recruiting medical students who meet the needs of Hawai'i's rural communities. Hawai'i journal of medicine & public health : a journal of Asia Pacific Medicine & Public Health. 2012;71(4 Suppl 1):21-5.

29. Shannon CK, Jackson J. A study of predictive validity of physician assistant students' reported practice site intent. The journal of physician assistant education : the official journal of the Physician Assistant Education Association. 2011;22(3):29-32.

30. Shannon CK, Jackson J. Validity of Medical Student Questionnaire Data in Prediction of Rural Practice Choice and Its Association With Service Orientation. The Journal of rural health : official journal of the American Rural Health Association and the National Rural Health Care Association. 2015;31(4):373-81.

31. Smith B, Muma RD, Burks L, Lavoie MM. Factors that influence physician assistant choice of practice location. JAAPA : official journal of the American Academy of Physician Assistants. 2012;25(3):46-51.

32. Snyder J, Nehrenz G, Danielsen R, Pedersen D. Educational debt: does it have an influence on initial job location and specialty choice? The journal of physician assistant education : the official journal of the Physician Assistant Education Association. 2014;25(4):39-42.

33. Stenger J, Cashman SB, Savageau JA. The primary care physician workforce in Massachusetts: implications for the workforce in rural, small town America. The Journal of rural health : official journal of the American Rural Health Association and the National Rural Health Care Association. 2008;24(4):375-83.

34. Terhune KP, Zaydfudim V, Abumrad NN. International medical graduates in general surgery: increasing needs, decreasing numbers. Journal of the American College of Surgeons. 2010;210(6):990-6.

35. Wendling AL, Phillips J, Short W, Fahey C, Mavis B. Thirty Years Training Rural Physicians: Outcomes From the Michigan State University College of Human Medicine Rural Physician Program. Academic medicine : journal of the Association of American Medical Colleges. 2016;91(1):113-9.

36. Whitacre BE, Pace V, Hackler JB, Janey M, Landgraf CE, Pettit WJ. An evaluation of osteopathic school programs designed to promote rural location by graduates. International Journal of Osteopathic Medicine. 2011;14(1):17-23.

37. Zink T, Center B, Finstad D, Boulger JG, Repesh LA, Westra R, et al. Efforts to graduate more primary care physicians and physicians who will practice in rural areas: examining outcomes from the university of Minnesota-duluth and the rural physician associate program. Academic medicine : journal of the Association of American Medical Colleges. 2010;85(4):599-604.

38. Antonenko DR. Rural surgery: the North Dakota experience. The Surgical clinics of North America. 2009;89(6):1367-72, x.

39. Bonham C, Salvador M, Altschul D, Silverblatt H. Training Psychiatrists for Rural Practice: A 20-Year Follow-up. Academic Psychiatry. 2014;38(5):623-6.

40. Crane S, Jones G. Innovation in rural family medicine training: the Mountain Area Health Education Center's rural-track residency program. North Carolina medical journal. 2014;75(1):29-30.

41. Crump WJ, Fricker RS, Ziegler CH. Outcomes of a preclinical rural medicine elective at an urban medical school. Family medicine. 2010;42(10):717-22.

42. Crump WJ, Fricker RS, Ziegler C, Wiegman DL, Rowland ML. Rural track training based at a small regional campus: equivalency of training, residency choice, and practice location of graduates. Academic medicine : journal of the Association of American Medical Colleges. 2013;88(8):1122-8.

43. Deveney K, Deatherage M, Oehling D, Hunter J. Association between dedicated rural training year and the likelihood of becoming a general surgeon in a small town. JAMA surgery. 2013;148(9):817-21.

44. Deveney K, Hunter J. Education for rural surgical practice: the Oregon Health & Science University model. The Surgical clinics of North America. 2009;89(6):1303-8, viii.

45. Gillig PM, Comer EA. A residency training in rural psychiatry. Academic psychiatry : the journal of the American Association of Directors of Psychiatric Residency Training and the Association for Academic Psychiatry. 2009;33(5):410-2.

46. Hulme PA, Houfek JF, Fiandt K, Barron C, Muhlbauer S. Educating Integrated Family/Psychiatric-Mental Health Nurse Practitioners: Program Development and Evaluation. J Nurs Educ. 2015;54(9):493-9.

47. Kallail KJ, McCurdy S. Scholars in Rural Health: Outcomes From an Assured Admissions Program. Fam Med. 2010;42(10):729-31.

48. Nash LR, Olson MM, Caskey JW, Thompson BL. Outcomes of a Texas family medicine residency rural training track: 2000 through 2007. Texas medicine. 2008;104(9):59-63.

49. Quinn KJ, Kane KY, Stevermer JJ, Webb WD, Porter JL, Williamson HA, Jr., et al. Influencing residency choice and practice location through a longitudinal rural pipeline program. Academic medicine : journal of the Association of American Medical Colleges. 2011;86(11):1397-406.

50. Ross R. Fifteen-year outcomes of a rural residency: aligning policy with national needs. Family medicine. 2013;45(2):122-7.

51. Wick KH. International medical graduates as physician assistants. JAAPA-J Am Acad Physician Assist. 2015;28(7):43-6.

52. Halaas GW, Zink T, Finstad D, Bolin K, Center B. Recruitment and retention of rural physicians: outcomes from the rural physician associate program of Minnesota. The Journal of rural health : official journal of the American Rural Health Association and the National Rural Health Care Association. 2008;24(4):345-52.

53. Halaas GW. The Rural Physician Associate Program: successful outcomes in primary care and rural practice. Rural and remote health. 2005;5(2):453.

54. Deutchman M. Medical School Rural Tracks in the US. Policy brief: September 2013. Washington, DC: National Rural Health Association. Available at: <http://www.ruralhealthweb.org/index.cfm?objectid=28B352C5-3048-651A-FE2D53C27202BAF6>, 2013.

55. Patterson DG, Longenecker R, Schmitz D, Phillips RL, Skillman SM, Doescher MP. January 2013 Policy Brief: Rural Residency Training for Family Medicine Physicians: Graduate Early-Career Outcomes, 2008-2012: Rural Training Track, technical assistance program; 2013 [8/20/2015]. Available from: https://[www.raconline.org/rtt/pdf/rural-family-medicine-training-early-career-outcomes-2013.pdf](http://www.raconline.org/rtt/pdf/rural-family-medicine-training-early-career-outcomes-2013.pdf).

56. Phillips RL, Petterson S, Bazemore A. Do residents who train in safety net settings return for practice? Academic medicine : journal of the Association of American Medical Colleges. 2013;88(12):1934-40.

57. Rabinowitz HK, Petterson SM, Boulger JG, Hunsaker ML, Markham FW, Diamond JJ, et al. Comprehensive medical school rural programs produce rural family physicians. American family physician. 2011;84(12):1350.

58. Rabinowitz HK, Petterson S, Boulger JG, Hunsaker ML, Diamond JJ, Markham FW, et al. Medical school rural programs: a comparison with international medical graduates in addressing state-level rural family physician and primary care supply. Academic medicine : journal of the Association of American Medical Colleges. 2012;87(4):488-92.

59. Rabinowitz HK, Diamond JJ, Markham FW, Santana AJ. Retention of rural family physicians after 20-25 years: outcomes of a comprehensive medical school rural program. Journal of the American Board of Family Medicine : JABFM. 2013;26(1):24-7.

60. Rabinowitz HK, Diamond JJ, Markham FW, Rabinowitz C. Long-term retention of graduates from a program to increase the supply of rural family physicians. Academic medicine : journal of the Association of American Medical Colleges. 2005;80(8):728-32.

61. Glasser M, Hunsaker M, Sweet K, MacDowell M, Meurer M. A comprehensive medical education program response to rural primary care needs. Academic medicine : journal of the Association of American Medical Colleges. 2008;83(10):952-61.

62. MacDowell M, Glasser M, Hunsaker M. A decade of rural physician workforce outcomes for the Rockford Rural Medical Education (RMED) Program, University of Illinois. Academic medicine : journal of the Association of American Medical Colleges. 2013;88(12):1941-7.

63. Shipman SA, Jones KC, Erikson CE, Sandberg SF. Exploring the workforce implications of a decade of medical school expansion: variations in medical school growth and changes in student characteristics and career plans. Academic medicine : journal of the Association of American Medical Colleges. 2013;88(12):1904-12.

64. Talley BE, Ann Moore S, Camargo CA, Jr., Rogers J, Ginde AA. Availability and potential effect of rural rotations in emergency medicine residency programs. Academic emergency medicine : official journal of the Society for Academic Emergency Medicine. 2011;18(3):297-300.
